# An empirical model for solvation based on surface site interaction points†

**DOI:** 10.1039/d1sc03392a

**Published:** 2021-09-16

**Authors:** Derek P. Reynolds, Maria Chiara Storer, Christopher A. Hunter

**Affiliations:** Yusuf Hamied Department of Chemistry, University of Cambridge Lensfield Road Cambridge CB2 1EW UK herchelsmith.orgchem@ch.cam.ac.uk

## Abstract

Surface site interaction points (SSIP) provide a quantitative description of the non-covalent interactions a molecule makes with the environment based on specific intermolecular contacts, such as H-bonds. Summation of the free energy of interaction of each SSIP across the surface of a molecule allows calculation of solvation energies and partition coefficients. A rule-based approach to the assignment of SSIPs based on chemical structure has been developed, and a combination of experimental data on the formation of 1 : 1 H-bonded complexes in non-polar solvents and partition of solutes between different solvents was used to parameterise the method. The resulting model is simple to implement using just a spreadsheet and accurately describes the transfer of a wide range of different solutes from water to a wide range of different organic solvents (overall rmsd is 1.4 kJ mol^−1^ for 1713 data points). The hydrophobic effect as well as the properties of perfluorocarbon solvents are described well by the model, and new descriptors have been determined for range of organic solvents that were not accessible by direct investigation of H-bond formation in non-polar solvents.

## Abstract 2

A rule-based approach for assignment of Surface Site Interaction Points (SSIP) based on the chemical structure of a molecule provides a straightforward spreadsheet method for accurate calculation of solvation free energies.

## Graphical abstract

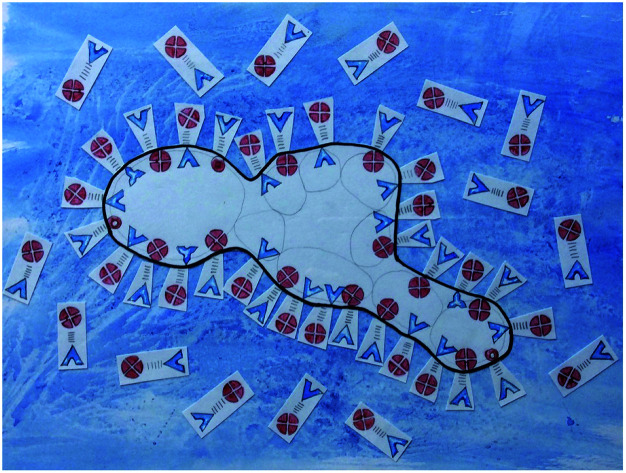

## Introduction

The solubility of an organic molecule in different chemical environments is a fundamentally important property that has implications in the petrochemical industry, in synthesis, and for the bioavailability of drug candidates. Although numerous approaches have been developed for the prediction of solubility directly from chemical structure, the accuracy and generality of these methods are still limited. The most pragmatic approaches are based on empirical parameterisation of functional group contributions, but these methods are limited to solvents where a large body of experimental data is available.^1–4^ The most sophisticated approaches are based on atomistic simulation using molecular dynamics, but these methods require significant computational resource.^5,6^ There are some promising approaches that marry the predictive power of *ab initio* calculation with empirical modelling.^7–15^

Abraham developed a different approach by using experimental data on 1 : 1 complexation to develop summation solvation parameters to describe the total H-bond donor or acceptor capacity of a molecule.^16–18^ Here we show that the description of a molecule as a set of specific interaction points, which are associated with individual functional groups, can be used to sum interactions across the molecular surface and accurately predict solvation properties directly from chemical structure. The approach integrates the treatment of intermolecular interactions between solutes and phase transfer equilibria, which means that diverse experimental data can be used for parameterisation. The description of molecules as a collection of the functional groups allows extrapolation to compounds for which experimental data is not available.

**Fig. 1:**
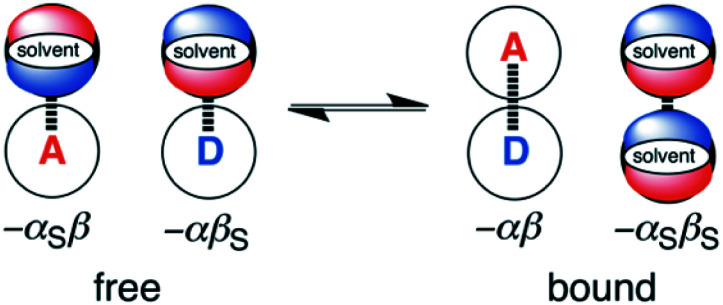
The solvent competition model for the complex formed between a H-bond donor solute, D, and a H-bond acceptor solute, A. The relative stabilities of the four complexes can be estimated using the H-bond parameters, *α*, *β*, *α*_S_ and *β*_S_, as indicated.

The generalised H-bond donor and acceptor parameters *α* and *β* can be used to describe the non-covalent interaction properties of both solvents and solutes.^19,20^ The free energy of formation (−Δ*G*°) for complexes in solution is determined by the competition between solute–solute, solute–solvent and solvent–solvent interactions, as illustrated in Fig. 1.

Given experimentally determined H-bond parameters for two solutes (*α* and *β*) and the solvent (*α*_S_ and *β*_S_), it is possible to make a reliable quantitative estimate of the free energy of formation of a 1 : 1 complex between a H-bond donor (D) and H-bond acceptor (A) using eqn (1).

1 Δ*G*°/kJ mol^−1^ = −(*α* − *α*_S_)(*β* − *β*_S_) + 6

where the constant of 6 kJ mol^−1^ was experimentally determined in carbon tetrachloride solutions.

This surprisingly simple model is based solely on the properties of the pairwise local contacts between molecules, and it does not require any consideration of long range interactions, solvent dielectric constant, solvent structure, cavitation or entropic terms. The success of the model suggests that these terms are small relative to the free energy contributions due to local interactions, or that they cancel out, or that they are captured in some way by the constant of 6 kJ mol^−1^, which does not vary much between solvents.

**Fig. 2:**
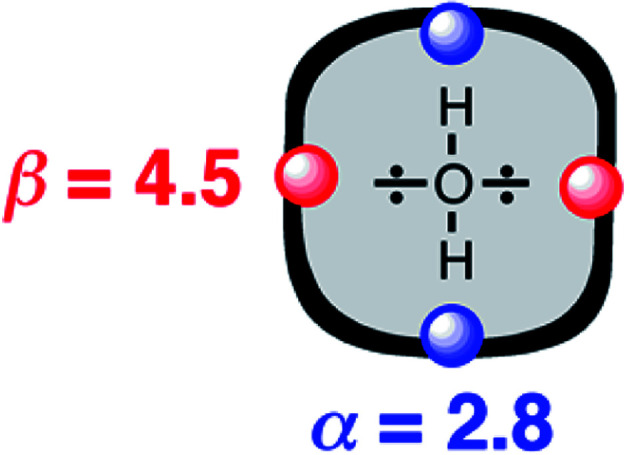
Water is represented by four SSIPs that describe the two H-bond donor (blue) and two H-bond acceptor (red) sites.

The approach illustrated in Fig. 1 has been extrapolated from single point interactions to a complete description of molecular surfaces by assigning a set of surface site interaction points (SSIP) to describe all of the non-covalent interactions that a molecule can make with the surroundings. Each SSIP is assigned an interaction parameter, which is equivalent to the empirically measured *α* and *β* parameters illustrated in Fig. 1. Fig. 2 shows the result for water, which is represented by two donor SSIPs and two acceptor SSIPs. Two approaches have been developed for obtaining the SSIP description of a molecule: manual assignment based on functional groups,^21^ and a computational method based on footprinting of *ab initio* calculated molecular electrostatic potential surfaces.^22,23^ The molecular SSIP description can be used to estimate the free energy contribution of non-covalent interactions to the stability of a solid by assuming that SSIPs are paired in a hierarchical fashion to maximise the total interaction energy, and this approach has been applied to successfully predict cocrystal formation.^24^ The free energy of interaction of a molecule with a solvent can be calculated by using the SSIP descriptions of solute and solvent in the surface site interaction model for the properties of liquids at equilibrium (SSIMPLE), and this approach has been applied in the calculation of solution phase properties like partition.^21,23^

In SSIMPLE, solvation free energies are obtained by calculating the equilibrium distribution of all pairwise SSIP contacts, also allowing for non-bonded states to account for the significant void space present in a liquid. There is an implicit assumption in eqn (1) that all four complexes shown in Fig. 1 are fully bound and that non-polar van der Waals interactions cancel out, so that the equilibrium is dominated by polar interactions. The introduction of non-bonded states in SSIMPLE therefore required an additional treatment of van der Waals interactions, and calculation of solvation energies using this approach is significantly more complicated than eqn (1) would suggest. Here, we present an alternative approach, which is operationally simpler and has the advantage that empirical elements are introduced to improve the accuracy. For teaching purposes, it is also useful to have an approach where partition can be calculated using only a pocket calculator or simple spreadsheet.

### Solvation free energies

**Fig. 3:**
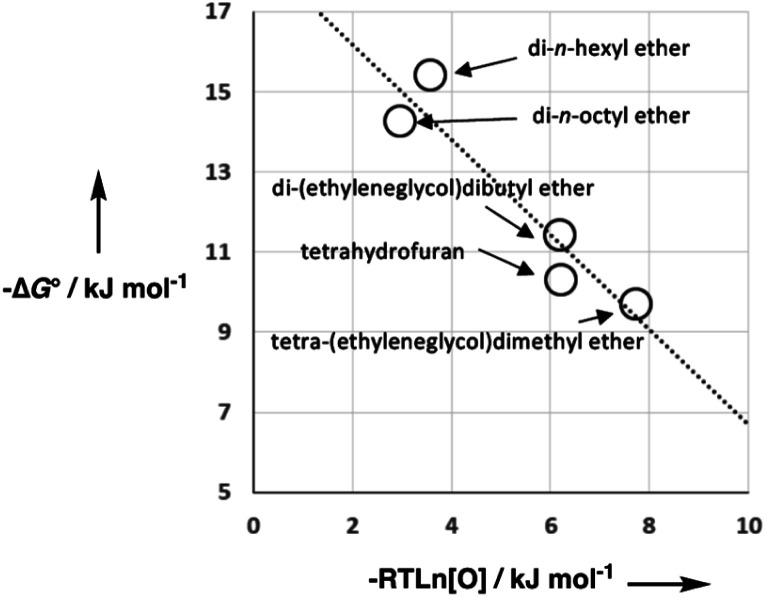
Free energy of formation for 1 : 1 H-bonded complexes in ether solvents (−Δ*G*°) plotted as a function of solvent oxygen concentration in entropy units (−*RT* ln[O]). The linear regression line has formula −1.2*x* + 18.5 with *R*^2^ = 0.90.

As we have previously suggested, the constant of 6 kJ mol^−1^ in eqn (1) is related to the difference between the concentration of the solvent and the standard state of 1 M used to describe the chemical potentials of the solutes.^21,25^Fig. 3 shows experimental data for formation of a 1 : 1 H-bonded complexes in various ether solvents. There is a linear dependence of the value of −Δ*G*° on ether oxygen concentration expressed in entropy units (*i.e. RT* ln[O]), and the slope is close to −1. This result suggests that if we define [S·S] as the effective concentration of the solvent–solvent interactions that are made on formation of a complex between two solutes, eqn (1) can be rewritten as eqn (2). For solvents like ethers that contain a single polar functional group, the effective concentration of solvent–solvent interactions is similar to the functional group concentration, so eqn (2) provides a reasonable description of the experimental data in Fig. 3. This relationship is also consistent with the behaviour of H-bonded complexes in solvent mixtures, where the free energy change for complex formation was found to be directly proportional to the logarithm of the concentration of the more polar solvent present in the mixture.^26–28^

2 Δ*G*° = −*αβ* + *α*_S_*β* + *αβ*_S_ − *α*_S_*β*_S_ + *RT* ln[S·S]

In general, the effective concentration of solvent–solvent interactions is not well-defined, so reliable values of [S·S] can only be deduced from experimental data. Experimentally determined association constants for formation of 1 : 1 H-bonded complexes in carbon tetrachloride were used to derive eqn (1), which gave a value of 10 M for [S·S] for this solvent. In this paper, we describe an approach based on partitioning of solutes between different solvents, which allows experimental determination of [S·S] for a wide range of different solvents.

The first term in eqn (2) describes the free energy of the A·D complex, and the other terms describe the free energy changes associated with desolvation of the two solutes on complexation. If we define Δ*g*_S_ as the free energy of transfer of a SSIP from an external reference state into a solvent S, then eqn (2) can be rewritten as eqn (3).

3 Δ*G*° = −*αβ* − Δ*g*_S_(*α*) − Δ*g*_S_(*β*)

where

4 Δ*g*_S_(*α*) = −*αβ*_S_ − *C*_*β*_

5 Δ*g*_S_(*β*) = −*α*_S_*β* − *C*_*α*_

The two constants *C*_*α*_ and *C*_*β*_ describe the solvent–solvent interactions that are disrupted when a solute enters the solvent. The sum of these constants should match the corresponding terms in eqn (2), which provide a composite description of the polarity and the concentration of the interactions that each solvent SSIP makes with the surrounding bulk solvent. Since these solvent–solvent terms are self-association, then in solvents where there is only one type of solvent–solvent interaction, the two constants must equal, as defined in eqn (6).6
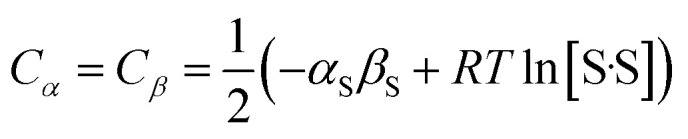


This reformulation of eqn (1) leads to a description of the free energy of solvation of an individual solute SSIP, and summing over a set of SSIPs that represent the surface of a molecule provides a straightforward method for calculating molecular solvation energies and hence partition. Eqn (7) shows the free energy change for transfer of a solute with *N*_*α*_ H-bond donor SSIPs and *N*_*β*_ H-bond acceptor SSIPs from the non-solvated reference state to solvent S.7
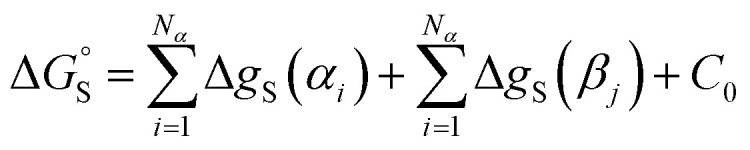


**Fig. 4:**
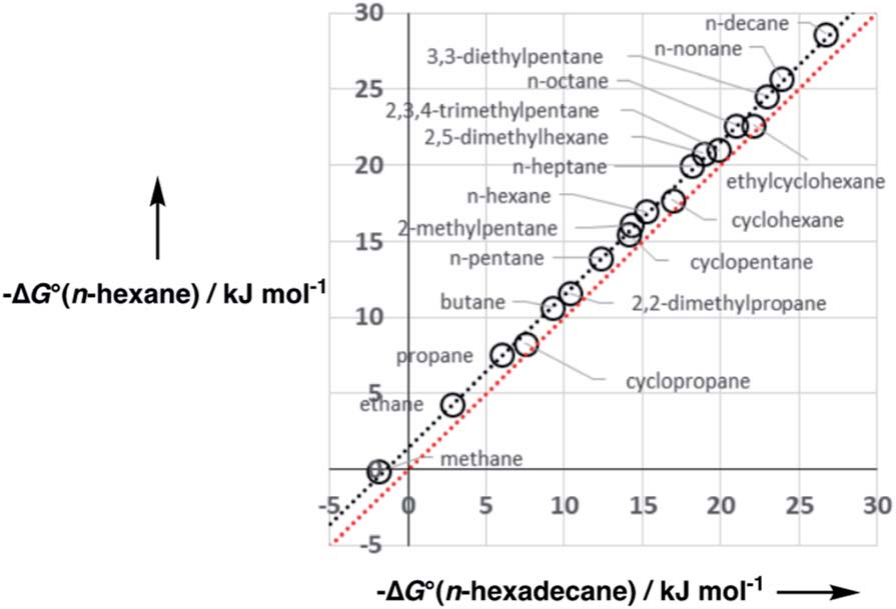
Comparison of the free energies of transfer of alkanes from the gas phase into *n*-hexadecane with the corresponding values for transfer into *n*-hexane. The red line is *y* = *x*, and the line of best fit (black) is *y* = 1.01*x* + 1.39 kJ mol^−1^.

A constant, *C*_0_, is introduced in eqn (7) to take care of any additional free energy contributions. The requirement for this constant can be demonstrated by examining experimental data for the free energies of transfer of alkanes from the gas phase into different solvents. Fig. 4 compares the gas–liquid partitioning of alkanes into *n*-hexadecane with the corresponding values for partitioning into *n*-hexane. Although the slope is very close to unity (1.01), there is an offset of 1.39 kJ mol^−1^ in favour of transfer into *n*-hexane. The fact that the offset is a constant for different alkane solutes with very different numbers of interaction sites implies that this phenomenon is due to differences in the intrinsic properties of the two solvents. Similar behaviour is observed for pairwise comparisons of some other non-polar solvents (see ESI Section S1†). Values of the offset vary from solvent to solvent, so we define the constant *C*_0_ relative to a reference solvent, *n*-hexadecane, to describe this intrinsic difference between different solvents.

The overall free energy of partition for a molecule is obtained by summing over all solute SSIPs using eqn (7), and the free energy of transfer between two different solvents S1 and S2 can then be obtained using eqn (8).8
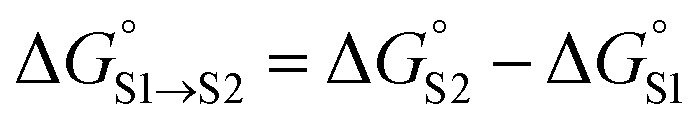


Many of the parameters required for the implementation of eqn (4)–(8) can be determined from the experimentally determined association constants for formation of 1 : 1 H-bonded complexes between polar solutes in non-polar solvents. However, H-bonded complexes are not sufficiently stable in polar solvents for characterisation of the solvation properties of a wider range of solvents, and interactions between non-polar solutes are too weak to lead to complexation in solution. Here we investigate the use of experimental partition data as a way of deducing parameters to describe these systems.

### SSIP description of aliphatic hydrocarbons

A SSIP description of polar functional groups is relatively straightforward to implement using the basic principles of chemical bonding and hybridisation. For example, water is described by four SSIPs, which represent the two H-bond donor sites and the two lone pairs (Fig. 2). For non-polar functional groups, the choice is less obvious. Previously, we have used molecular surface area as the reference point to determine the number of SSIPs required to describe the non-covalent interaction properties of a molecule. The model developed here is different, because all SSIPs are assumed to be fully paired in the liquid state. If the van der Waals contribution to solvation is to cancel out when a molecule is transferred from one liquid to another, then the total concentration of SSIPs in different liquids should be approximately the same. Liquid water provides an obvious benchmark with four SSIPs per molecule and a concentration of 55 M at 298 K. The total number of SSIPs (*N*_*α*_ + *N*_*β*_) required to describe hydrocarbons was therefore defined based on the molar concentration of the pure liquid using eqn (9).

9 [SSIP] = (*N*_*α*_ + *N*_*β*_)[liquid] ≈ 220 M

Solvent H-bond parameters for alkanes have been determined experimentally by applying eqn (1) to measurements of association constants for the formation of 1 : 1 H-bonded complexes.^29^ The experimental values of *α*_S_ = 1.20, *β*_S_ = 0.60, and *RT* ln[S·S] = +6 kJ mol^−1^ can be used in eqn (6) to obtain the constants required to describe alkane solvents: *C*_*α*_ = *C*_*β*_ = 2.64 kJ mol^−1^. In contrast, eqn (1) cannot be used directly to determine the parameters required to describe the properties of alkane solutes, because they are not polar enough to form stable 1 : 1 complexes. We therefore used experimental data on phase transfer free energies to develop a SSIP description of alkane solutes.

**Fig. 5:**
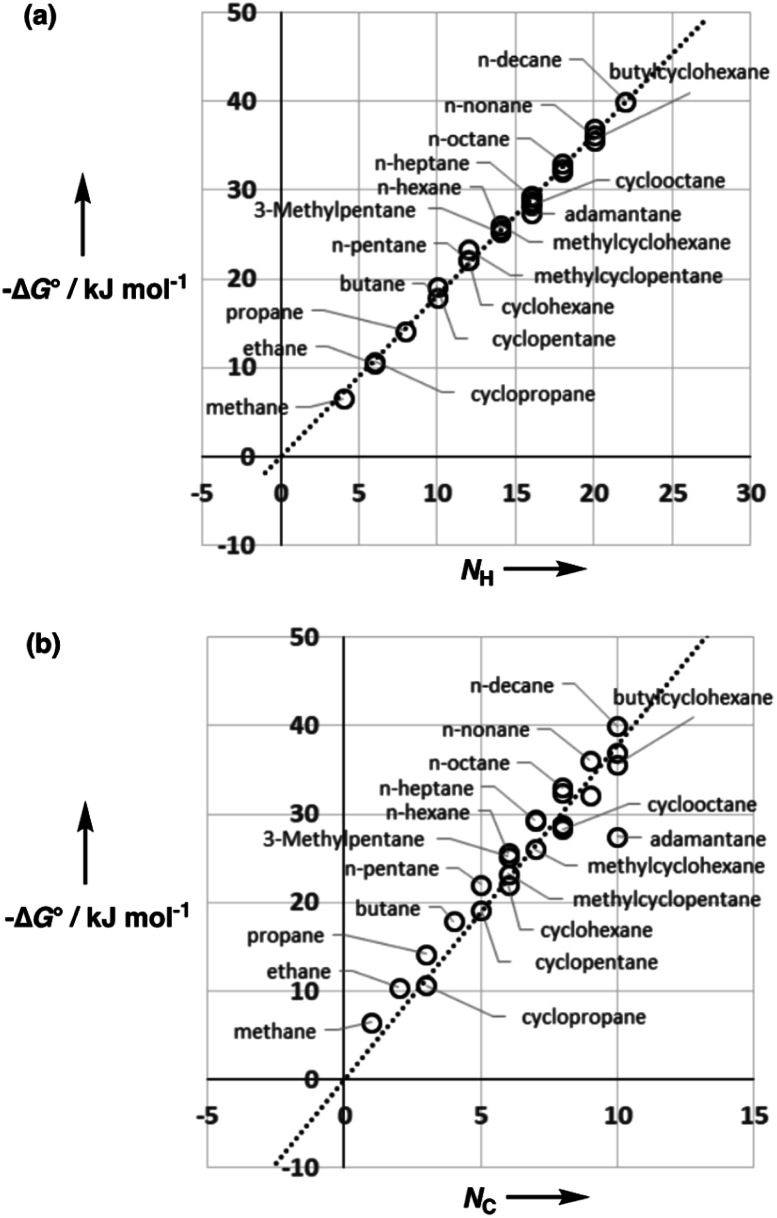
Comparison of the experimental free energy of transfer of alkanes from water to *n*-hexadecane (Δ*G*°) (a) with the number of hydrogen atoms (*N*_H_, *R*^2^ = 0.9956) and (b) with the number of carbons atoms (*N*_C_, *R*^2^ = 0.8778) in the molecule.

**Fig. 6:**
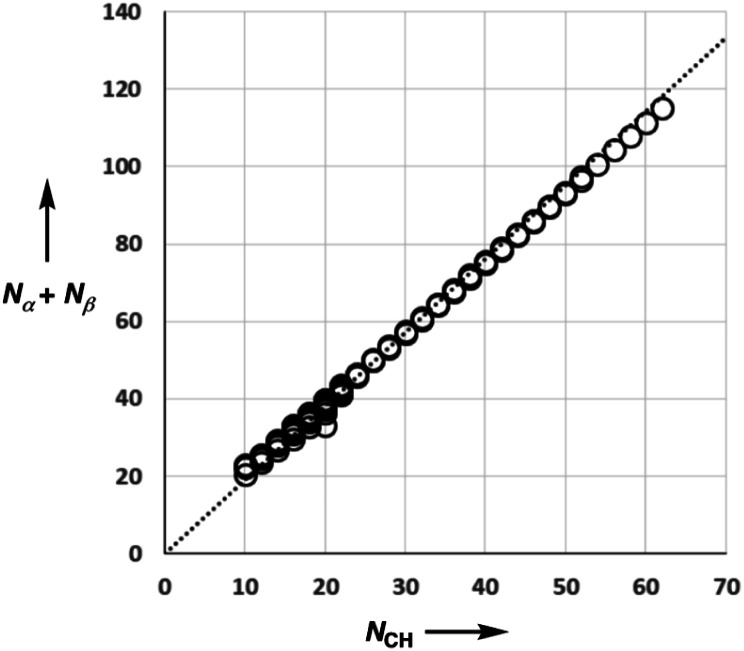
Comparison of the total number of SSIPs (*N*_*α*_ + *N*_*β*_) calculated using eqn (9) with the number of CH bonds (*N*_CH_) for 182 alkanes. The line of best fit through the origin is shown (*y* = 1.90*x*).

**Fig. 7:**
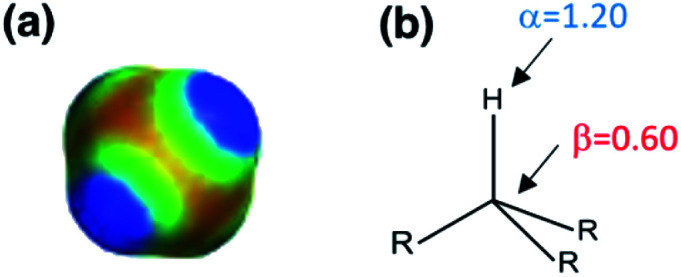
(a) The molecular electrostatic potential surface of methane (red = −25 kJ mol^−1^, blue = +25 kJ mol^−1^) calculated on the 0.002 e bohr^−3^ electron density isosurface using DFT with a B3LYP 6-31G* basis set. (b) The SSIP description of a CH bond in an aliphatic hydrocarbon.

Fig. 5 shows experimental data for the free energy of transfer of alkanes from water to *n*-hexadecane. The correlation with the number of hydrogen atoms (*N*_H_) is significantly better than the correlation with the number of carbon atoms (*N*_C_), because the hydrogen atoms are always exposed on the surface of the molecule, whereas the carbon atoms are not. For example, the quaternary carbon atom in neopentane is completely buried. The approach to construction of a SSIP description of alkane solutes is therefore based on the number of CH bonds. Application of eqn (9) to alkanes indicates that the total number of SSIPs required to describe an alkane (*N*_*α*_ + *N*_*β*_) is approximately twice the number of CH bonds (Fig. 6).^30^ Assigning two SSIPs to each CH bond results in a total SSIP concentration that varies from 208 M for *n*-pentane to 232 M for *n*-hexadecane (*cf.* benchmark value of 220 M for water). Fig. 7 shows the calculated MEPS of methane. There are four regions of positive potential over the hydrogen atoms on the end of each CH bond and four regions of negative potential over the carbon atoms at the back of each CH bond. We therefore assign one *α* and one *β* for every CH bond in an alkane, and assume that the SSIP parameters determined for alkane solvents can be used to describe the non-covalent interaction properties of alkane solutes, *i.e. α* = 1.20, *β* = 0.60.

### SSIP description of aromatic hydrocarbons

**Fig. 8:**
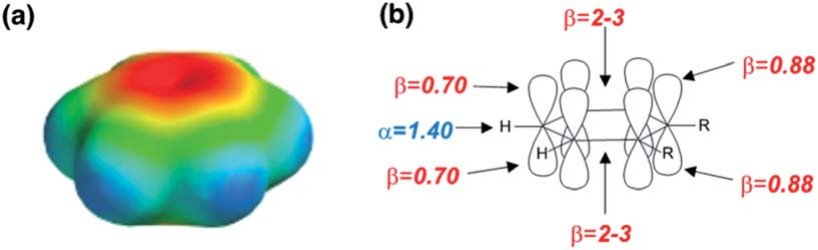
(a) The molecular electrostatic potential surface of benzene (red = −80 kJ mol^−1^, blue = +80 kJ mol^−1^) calculated on the 0.002 e bohr^−3^ electron density isosurface using DFT with a B3LYP 6-31G* basis set. (b) The SSIP description of aromatic hydrocarbons (R is an alkyl substituent).

The archetypal aromatic hydrocarbon is benzene, and this compound was used to develop a SSIP description of aromatic groups. Eqn (9) indicates that benzene should represented by a total of 20 SSIPs, which results in a total SSIP concentration of 224 M at 298 K. The MEPS of benzene is shown in Fig. 8a. Consideration of the hybridisation of the carbon atoms suggests that each aromatic CH group should represented by one *α* on the end of the CH bond and two *β* SSIPs above and below the plane of the ring (Fig. 8b). This description gives a total of 18 SSIPs, and the MEPS shown in Fig. 8a suggests that the two additional SSIPs should be used to represent the more polar negative electrostatic potential located over the centres of the two faces of the π-system. The properties of these two SSIPs were determined by applying eqn (1) to measurements of association constants for the formation of 1 : 1 H-bonded complexes with H-bond donors (see Table 1 and Fig. S2†). The other parameters required to describe the less polar SSIPs associated with aromatic CH groups (*α* = 1.40, *β* = 0.70) were obtained by fitting to experimental data for the free energy of transfer of aromatic hydrocarbons from water to *n*-hexadecane. Using this approach, it was also possible to obtain parameters for aromatic molecules with alkyl substituents. The aromatic carbon bearing the alkyl substituent is described by two SSIPs, one above and one below the plane of the ring, each with a value of *β* = 0.88 (Fig. 8b).

**Table 1:** H-bond parameters for the polar interaction sites on the π-faces of aromatic hydrocarbons

Aromatic acceptors	*β*
Benzene	2.00
Toluene	2.20
*ortho*-, *meta*- or *para*-xylene	2.40
Mesitylene	2.70
Hexamethylbenzene	3.10

### SSIP description of water

The parameters required to describe the properties of water as a solute have been determined experimentally by applying eqn (1) to measurements of association constants for the formation of 1 : 1 H-bonded complexes: *α* = 2.80 and *β* = 4.50.^19^ In contrast, eqn (1) cannot be used directly to determine the parameters required to describe the properties of water as a solvent, because it is too polar to allow formation of singly H-bonded 1 : 1 complexes. We therefore used experimental data on phase transfer free energies to develop solvent parameters for water.

**Fig. 9:**
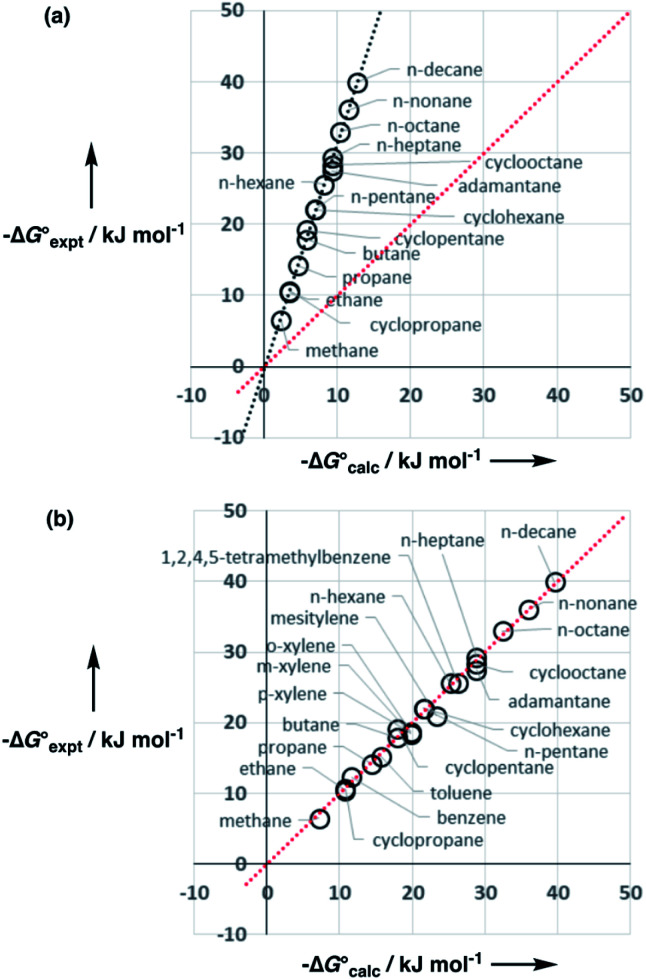
(a) Comparison of the experimental free energy of transfer of alkanes from water to *n*-hexadecane 
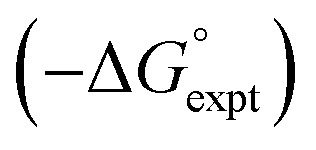
 with the values calculated using solvent parameters for water of *α*_S_ = 2.80, *β*_S_ = 4.50 and *C*_*α*_ = *C*_*β*_ = −0.47 kJ mol^−1^
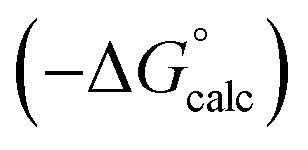
. The red line is *y* = *x*, and the line of best fit is black. (b) Comparison of the experimental free energy of transfer of aliphatic and aromatic hydrocarbons from water to *n*-hexadecane 
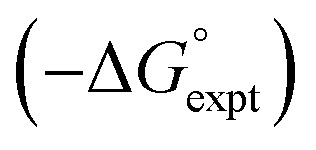
 with the values calculated using solvent parameters for water of *α*_S_ = 3.80, *β*_S_ = 3.47 and *C*_*α*_ = *C*_*β*_ = −0.76 kJ mol^−1^
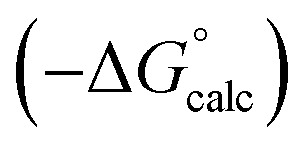
. The red line is *y* = *x*.

Water is a unique solvent in that the concentration of solvent–solvent interactions is known, because the H-bonds are almost fully bound in liquid water at 298 K.^31^ We can therefore set the value of [S·S] equal to 110 M in eqn (6). If we assume that the H-bond parameters that have been experimentally measured for water as a solute can be used to describe water as a solvent, *i.e. α*_S_ = 2.80 and *β*_S_ = 4.50, eqn (6) gives the constants required to describe water as *C*_*α*_ = *C*_*β*_ = −0.47 kJ mol^−1^. However, calculation of the partition of alkanes between water and *n*-hexadecane using these parameters failed to reproduce the experimentally measured values (Fig. 9a). The preference for alkanes to partition into *n*-hexadecane is overestimated, which suggests that the solute parameters for water cannot be used to describe the properties of water as a solvent in this model. By using experimental data for the free energy of transfer of aliphatic and aromatic hydrocarbon solutes from water to *n*-hexadecane, it was possible to optimise the solvent parameters for water: *α*_S_ = 3.80 and *β*_S_ = 3.47. Fig. 9b shows that the resulting parameters provide an excellent description of partitioning of a variety of different types of hydrocarbon into water.

### SSIP description of polar solutes

**Fig. 10:**
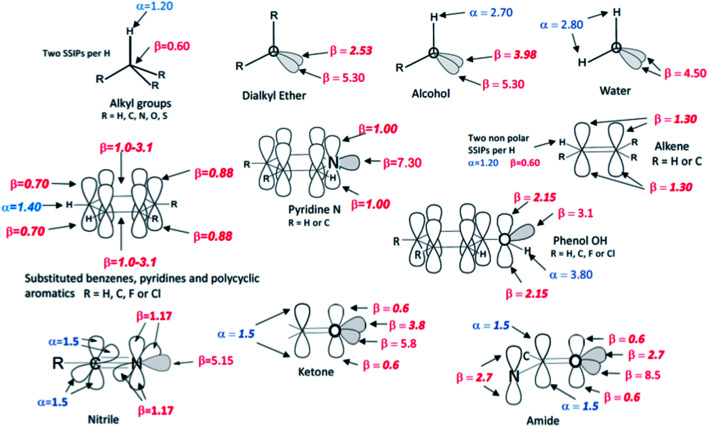
The SSIP description of functional groups. The values shown as bold italic were optimised in order to minimise the rmsd between calculated and experimental free energies for the partition models listed in Table 2. The other values are based on previously measured experimental parameters.

The SSIP models for water and hydrocarbons described above allow accurate calculation of the partition of hydrocarbon solutes between water and *n*-hexadecane as shown in Fig. 9b. The same approach was then used to assign SSIP parameters for an expanded solute set of 134 molecules that included alkenes, polycyclic aromatics, phenols, pyridines, water, alcohols and ethers. An initial set of SSIP parameters was derived from partition between water and hydrocarbons and then further refined using additional data from partition into other non-polar solvents (see below). The SSIP parameters that describe the functional groups present in these compounds are summarised in Fig. 10.

For some functional groups, experimental values of *α* and *β* have previously been determined by applying eqn (1) to measurements of association constants for the formation of 1 : 1 H-bonded complexes, and these values were used unmodified and assigned to the relevant SSIPs. However, these experimental parameters only provide information on the most polar site present in a solute, so when more than one type of *α* or *β* is required to describe a functional group, optimisation of the parameter describing the second site was often required. For example, an alcohol is described by one *α* and two *β* SSIPs (Fig. 10), but using the experimental value of 5.30 for both *β* SSIPs overestimated the polarity of this functional group. An accurate description of the partition coefficients of alcohols was obtained by reducing one of the two *β* parameters from 5.30 to 3.98. Alkyl groups are well-described by the same *α* and *β* parameters developed for alkanes above and to simplify the assignment problem the effects of electronegative substituents (O, N or S) on these parameters were assumed to be negligible. Similarly, the parameters developed for aromatic hydrocarbons generally provide a good description of substituted aromatic rings, with the exception that the value of *β* for the two H-bond acceptor sites over the centre of the ring had to be varied depending on the electronic properties of the ring substituents. The same approach starting from experimental functional group H-bond parameters and atom type hybridisation models was applied to ketones, nitriles, amines, amides, thioethers, alkyl fluorides, alkyl chlorides, fluoro and chloro substituted benzenes and phenols in order to expand the range of solute functional groups (see ESI Section S3† for full details). The SSIP parameters for these functional groups were optimised using data from partition into both non-polar and polar solvents (see below).

### Parameters for non-polar solvents

**Fig. 11:**
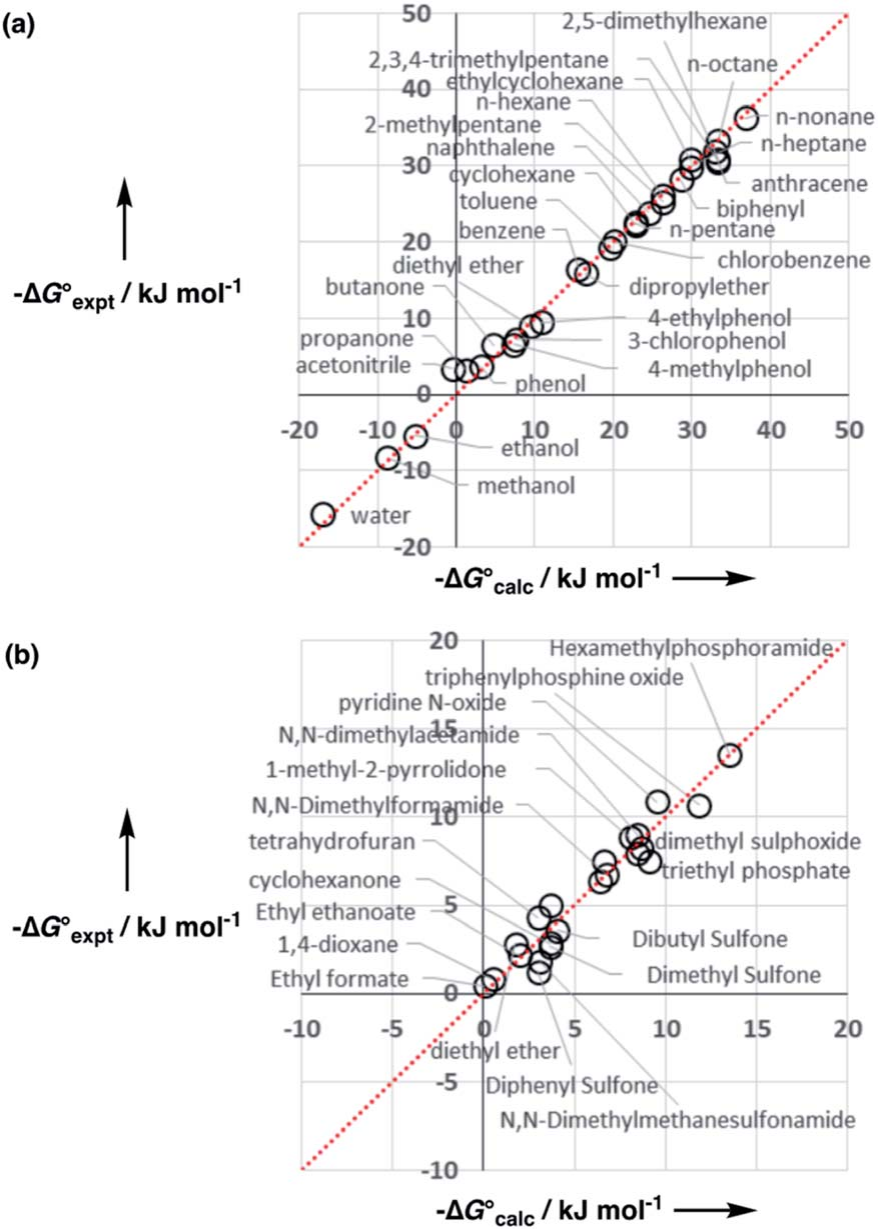
Comparison of calculated 
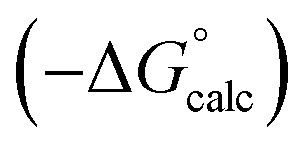
 and experimental free energies 
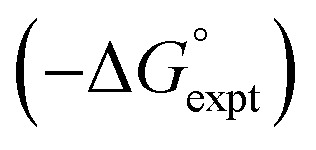
 for (a) transfer of for various solutes from water to dichloromethane (rmsd = 1.0 kJ mol^−1^), and (b) formation of 1 : 1 complexes between 4-fluorophenol and various H-bond acceptors in dichloromethane (rmsd = 0.95 kJ mol^−1^). The red lines are *y* = *x*.

It is possible to determine experimental values of *α*_S_ and *β*_S_ for simple non-polar solvents by applying eqn (1) to measurements of association constants for the formation of 1 : 1 H-bonded complexes. For solvents that have only one type of donor and one type of acceptor SSIP, like aliphatic hydrocarbons, the values of *C*_*α*_ and *C*_*β*_ are equal and can be estimated using eqn (6). This approach was used to obtain parameters for carbon tetrachloride: *α*_S_ = 1.40, *β*_S_ = 0.60, *C*_*α*_ = *C*_*β*_ = 2.58 kJ mol^−1^. However, most organic solvents have more than one type of *α* or *β* site, so the experimentally determined values of *α*_S_ and *β*_S_ represent a weighted average of different interactions between solvent and solute, and the values of *C*_*α*_ and *C*β are unlikely to be equal. Provided the parameters are optimised using experimental partition data, it turns out that only one *α*_S_ value and one *β*_S_ value can be used to accurately describe the properties of non-polar solvents. The experimental values of *α*_S_ and *β*_S_ for 10 non-polar solvents were used as a starting point for the development of a self-consistent set of solvent parameters by optimising against experimental partition data. The resulting values of *α*_S_, *β*_S_, *C*_*α*_ and *C*_*β*_ are listed in Table 2, and Fig. 11a illustrates the quality of the description of transfer free energies for dichloromethane.

**Table 2:** Parameters for water and non-polar organic solvents and comparison of calculated free energies with experimental data. The values shown as bold italic were optimised in order to minimise the rmsd between calculated and experimental free energies for the partition models listed. The other values are based on previously measured parameters from experimental 1 : 1 complexation data

Solvent	Solvent descriptors					Solvent/water partition		1 : 1 complexation	
	*α*_S_	*C*_*α*_	*β*_S_	*C*_*β*_	*C*_0_	*n*	rmsd/kJ mol^−1^	*n*	rmsd/kJ mol^−1^
Water	3.80	−0.76	3.47	−0.76	0				
Hexadecane	1.20	2.64	0.60	2.64	0	219	1.2		
Benzene	1.40	***2.50***	2.00	***1.09***	0	37	1.2	108	1.7
Toluene	1.40	***2.50***	2.00	***1.09***	0	34	1.1	46	1.7
Hexane	1.20	***2.62***	0.60	***2.62***	***1.38***	87	1.1	6	0.7
Cyclohexane	1.20	***2.61***	0.60	***2.61***	***1.33***	51	1.2	109	1.2
Carbon tetrachloride	1.40	2.58	0.60	2.58	***1.34***	86	1.2	475	0.8
Dichloromethane	1.80	***2.16***	1.40	***1.76***	***1.73***	28	1.3	42	1.1
Chloroform	2.10	***1.78***	1.30	***2.11***	***0.60***	71	1.6	13	1.2
1,2-Dichloroethane	1.70	***2.23***	1.60	***1.41***	***1.93***	58	1.6	34	1.0
Chlorobenzene	1.40	***2.51***	1.40	***1.71***	***1.48***	57	1.2	22	1.5
Perfluoroalkane	1.2	***2.41***	0.60	***2.41***	***0.81***	27	1.85	15	1.7

Experimental data on association constants for the formation of 1 : 1 H-bonded complexes provide an independent validation of these solvent parameters. Combining eqn (3)–(5) gives eqn (10), which can be used to calculate values of Δ*G*° for formation of 1 : 1 complexes. For each solvent, the values of *α*_S_, *β*_S_, *C*_*α*_ and *C*_*β*_ in Table 2 were used in conjunction with previously determined experimental values of solute *α* and *β* parameters to calculate the free energy change for complexes formed by a variety of H-bond acceptors with H-bond donors. In all cases, the rmsd between the calculated and experimental values is less than 1.7 kJ mol^−1^ (see ESI Section S4†) confirming the reliability of the solvent parameters. Fig. 11b illustrates the quality of the description of 1 : 1 complexation data for dichloromethane.

10 Δ*G*° = −*αβ* + *αβ*_S_ + *α*_S_*β* + *C*_*α*_ + *C*_*β*_

### Parameters for polar organic solvents

Organic solvents which contain polar functional groups must be treated differently, because it is no longer possible to use only one *α*_S_ value and one *β*_S_ value to describe a weighted average of solvation states. The reason for the failure of this simplistic description is that the non-polar regions of the solvent preferentially solvate non-polar solutes, and the polar groups on the solvent preferentially solvate polar solutes. In this case, both the polarities and the concentrations of the different sites on the solvent become important.

**Fig. 12:**
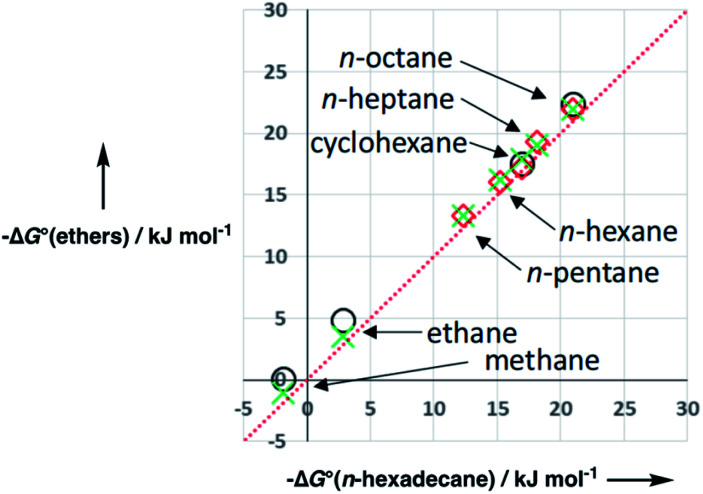
Free energy of transfer of alkane solutes from the gas phase into ether solvents (circles tetrahydrofuran, crosses diethyl ether, diamonds di-*n*-butyl ether) compared to transfer from the gas phase into *n*-hexadecane. The red line is *y* = *x*.

We will describe development of the model for ethers to illustrate the approach used to parameterise polar organic solvents. Ethers are the simplest class of polar organic solvent, because they have two types of acceptor SSIP, which describe the non-polar hydrocarbon region (*β*_S1_) and the polar oxygen sites (*β*_S2_), but only one type of CH donor SSIP (*α*_S1_). Fig. 12 shows that the free energy of transfer of alkanes from the gas phase into alkyl ethers is very similar to the value for transfer into *n*-hexadecane. This result suggests that the hydrocarbon component of an ether has similar properties to an alkane, so the solvation of non-polar solute SSIPs can be described using the same parameters used to describe alkane solvents, *i.e. α*_S1_ = 1.2 and *β*_S1_ = 0.6. The parameter for the polar oxygen acceptor SSIP can be estimated from the solute values in Fig. 10, which gives *β*_S2_ = 5.3.

The solvation of solute H-bond acceptor SSIPs is described in a straightforward manner by eqn (5) using *α*_S_ = *α*_S1_ = 1.2 for the solvent H-bond donor SSIPs. Solvation of solute H-bond donors is more complicated, because they interact with two different acceptor sites. We assume that the solvent is present in a large excess relative to the solute, so that each solute SSIP is solvated according to an effective equilibrium constant for interaction with each type of solvent SSIP. The equilibrium constants for the interaction of a solute H-bond donor SSIP *α* with the two different solvent H-bond acceptor SSIPs *β*_S1_ and *β*_S2_ are given by eqn (11) and (12).11
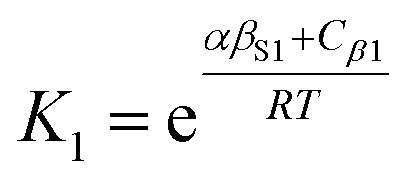
12
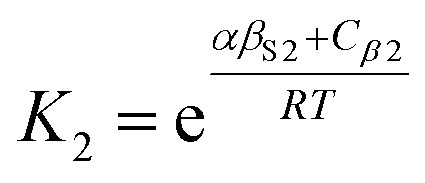
where the constants *C*_*β*1_ and *C*_*β*2_ describe the solvent–solvent interactions that are disrupted on formation of an interaction between solute and solvent (*cf.*eqn (4)).

The total free energy contribution due to solvation of the solute H-bond donor SSIP *α* is given by the sum of these equilibrium constants weighted by the fraction of interactions made with the relevant solvent SSIP, as shown in eqn (13).13
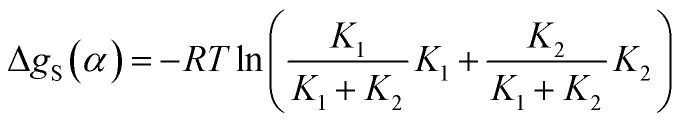


**Fig. 13:**
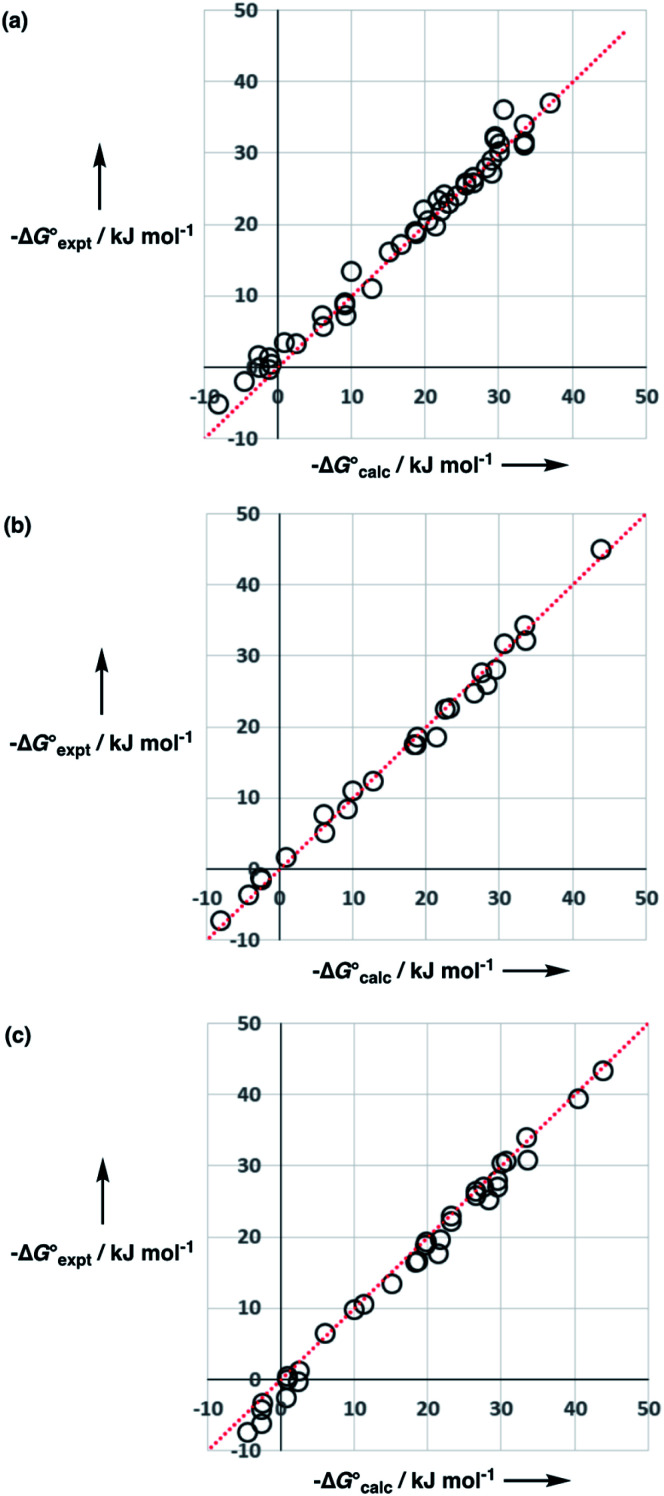
Comparison of calculated 
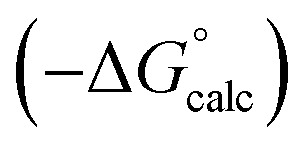
 and experimental free energies 
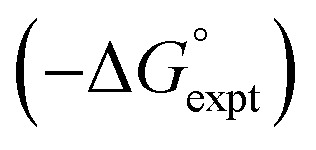
 for transfer from water to (a) tetrahydrofuran (rmsd = 2.0 kJ mol^−1^, *n* = 49), (b) diethyl ether (rmsd = 1.2 kJ mol^−1^, *n* = 25), and (c) di-*n*-butyl ether (rmsd = 1.7 kJ mol^−1^, *n* = 35). The red lines are *y* = *x*.

The constants *C*_*β*1_ and *C*_*β*2_ are determined by the polarity and concentration of solvent–solvent interactions and are likely to vary from solvent to solvent. However, when values of transfer free energies from water to different ether solvents were used to independently optimise values of *C*_*α*1_, *C*_*β*1_, *C*_*β*2_ and *C*_0_ for tetrahydrofuran, diethyl ether and di-*n*-butyl ether, the variation in the values of the constants between solvents was rather small (see ESI Section S5†). Fig. 13 shows that using a single generic value of each constant for ethers (see Table 3) gives a good description of the experimental data for all three solvents.

**Table 3:** Parameters for polar organic solvents and comparison of calculated transfer free energies with experimental data. The values shown as bold italic were optimised in order to minimise the rmsd between calculated and experimental free energies for the partition models listed. The other values are based on previously measured parameters from experimental 1 : 1 complexation data

Solvent class	Solvent descriptors									Solvent/water partition	
	*α*_S1_	*C*_*α*1_	*β*_S1_	*C*_*β*1_	*α*_S2_	*C*_*α*2_	*β*_S2_	*C*_*β*2_	*C*_0_	*n*	rmsd/kJ mol^−1^
Ethers^a^	1.20	***2.58***	0.60	***2.98***	—	—	5.30	**−3.65**	***2.26***	109	1.7
Nitriles^b^	1.20	***−0.79***	0.60	***2.69***	1.50	***2.67***	5.15	***−3.54***	***3.55***	76	1.7
Ketones^c^	1.20	***−0.78***	0.60	***2.82***	1.50	***2.71***	5.80	***−4.09***	***1.89***	118	1.4
Alcohols^d^	1.20	***2.75***	0.60	***2.95***	3.50	***−6.02***	6.90	***−6.42***	***0.18***	604	1.5

a Diethyl ether, di-*n*-butyl ether and tetrahydrofuran.

b Acetonitrile, propionitrile and butyronitrile.

c Acetone, butanone and cyclohexanone.

d Methanol, ethanol, propan-1-ol, butan-1-ol, pentan-1-ol, hexan-1-ol, heptan-1-ol, octan-1-ol, decan-1-ol, propan-2-ol, butan-2-ol, 2-methylpropan-1-ol, 2-methylpropan-2-ol and 3-methylbutan-1-ol.

For alcohols, there are two different types of acceptor SSIP, the polar oxygen and non-polar hydrocarbon, so eqn (11)–(13) can be used to describe the solvation of solute donor SSIPs. There are also two different types of donor SSIP, the polar OH and the nonpolar CH groups, so a similar set of eqn (14)–(16) are required to described the equilibria involved in the solvation of solute acceptor SSIPs.14
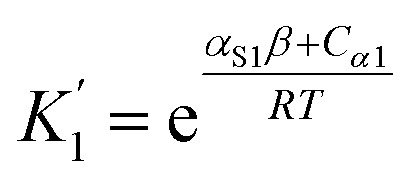
15
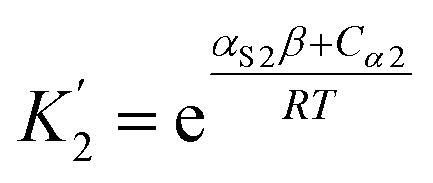
where the constants *C*_*α*1_ and *C*_*α*2_ describe the solvent–solvent interactions that are disrupted on formation of an interaction between solute and solvent.16
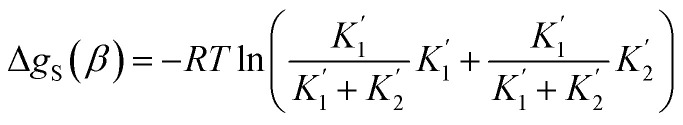


The effect of alcohols on the association constants for formation of 1 : 1 H-bonded complexes has been investigated in alcohol-alkane mixtures.^32^ These experiments suggest that alcohol self-association leads to an increase in the polarity of the hydroxyl group compared with monomeric alcohols in dilute solution. The effective value of the hydroxyl *α* increases from 2.7 to 3.5 and *β* increases from 5.3 to 6.9 in the self-associated bulk liquid. These parameters were therefore used as *α*_S2_ and *β*_S2_ to describe the polar SSIPs in alcohols. The alkane solvent parameters (*α*_S1_ = 1.2 and *β*_S1_ = 0.6) were used to describe the non-polar hydrocarbon SSIPs in alcohols. Partition data were available for 14 different alcohol solvents, and these data were used to optimise a single set of constants (*C*_*α*1_, *C*_*α*2_, *C*_*β*1_, *C*_*β*2_ and *C*_0_ in Table 3) that describe the solvent–solvent interactions in all of these alcohols.

Experimental values of *α*_S_ and *β*_S_ have been determined for nitrile and ketone solvents by applying eqn (1) to measurements of association constants for the formation of 1 : 1 H-bonded complexes (see ESI Section S6†). These values were therefore used to describe the polar SSIPs in these solvents, *α*_S2_ and *β*_S2_, and the non-polar hydrocarbon SSIPs were described using the alkane parameters as above (*α*_S1_ = 1.2 and *β*_S1_ = 0.6). As for ethers and alcohols, a generic set of constants (*C*_*α*1_, *C*_*α*2_, *C*_*β*1_, *C*_*β*2_ and *C*_0_) were obtained for dialkyl ketones and for alkyl nitriles by minimising the rmsd between experimental partition data and calculated transfer free energies (Table 3).

## Results

The training set used to develop the model contained 266 different solutes, 35 different solvents, and a total of 1884 independent measurements of partition free energies (see ESI Section S7† for details). These data were used to optimise 60 SSIP values used to describe the solutes and 45 parameters used to describe the solvents. The other 63 SSIP values and solvent constants used in the model were fixed using previous measurements on 1 : 1 H-bonded complexes. Thus the number of data points used in parameterisation of the model (1884) was more than 10 times the number of optimised parameters (105).

**Fig. 14:**
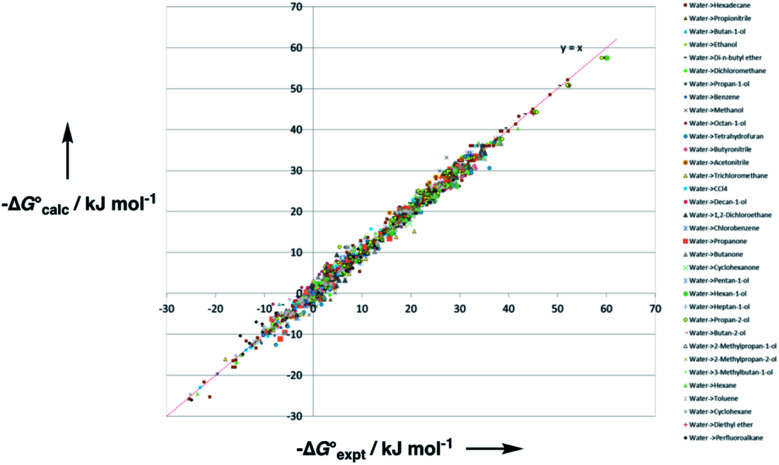
Comparison of free energies calculated using the SSIP model developed here 
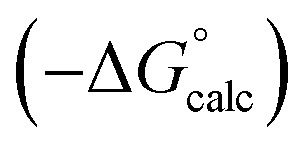
 with experimental values 
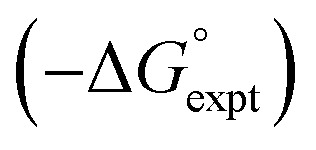
 for transfer of 219 different solutes from water to 35 solvents (overall rmsd is 1.4 kJ mol^−1^ for 1713 data points). The red line is *y* = *x*.

Experimental data for partition between water and *n*-hexadecane was available for 219 of the solutes in the training set. The solvent parameters in Tables 2 and 3 were used with SSIP representations of these solutes to calculate transfer free energies between water and 34 different organic solvents (see ESI Section S9† for details). The results are illustrated in Fig. 14. The agreement is excellent with an overall rmsd of 1.4 kJ mol^−1^ for 1713 data points, and there are no major outliers.

For 189 of the solutes in the training set, data was also available for partition between wet octanol and water, and these data were used to validate the generic alcohol solvent parameters. Comparison of calculated and experimental values was satisfactory with an rmsd of 1.6 kJ mol^−1^. In addition, calculations were performed on a validation set of 84 solutes that were not present in any of data sets used to parameterise the model. Again good agreement with the experimental data was obtained with an overall rmsd of 1.7 kJ mol^−1^ for octanol–water partition and 1.6 kJ mol^−1^ for *n*-hexadecane-water partition. The original training set was limited to simple compounds, some of the validation set solutes contained more than one functional group (*e.g.* 1,4-dicyanobutane), and these compounds are described well. These results suggest that the model developed here is robust and has applicability beyond the systems used for parameterisation.

**Fig. 15:**
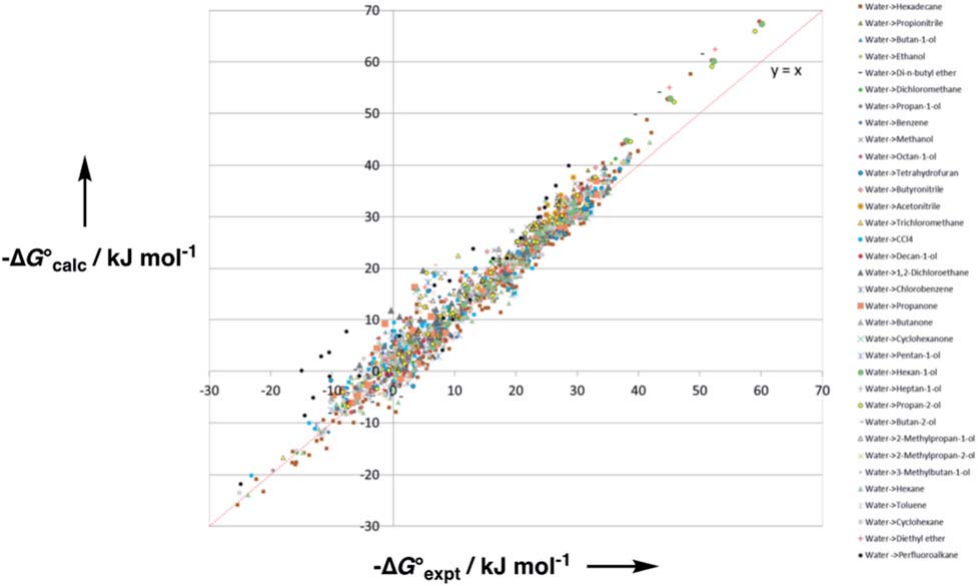
Comparison of free energies calculated using SSIMPLE 
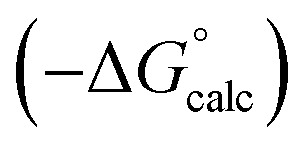
 with experimental values 
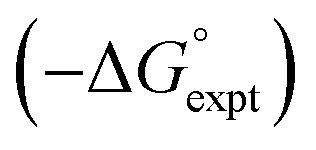
 for transfer of 219 different solutes from water to 35 solvents (overall rmsd is 3.5 kJ mol^−1^ for 1713 data points). The red line is *y* = *x*.

The performance of the model was evaluated by comparing the results with related methods. Fig. 15 compares the model with the performance of the SSIMPLE method for the same for 1713 experimental data points used in Fig. 14. SSIMPLE uses *ab initio* calculations to obtain SSIPs for both solute and solvent and evaluates all pairwise SSIP interactions in the liquid phase, including van der Waals interactions.^21^ There are very few empirical parameters in SSIMPLE, and although the trend is reasonable, the performance is significantly worse than the model developed in this paper, with an overall rmsd of 3.5 kJ mol^−1^. For octanol–water partition, a number of methods have been developed. *c* log *P* is a structure-based method that uses empirically derived parameters for functional group fragments, so it can be generalised to a wide range of solutes but cannot be generalised to other solvents.^33^ Abraham's linear solvation energy relationship (LSER) is based on summation over solvent–solute interactions and can be applied to a wide range of solvents, but empirical parameters are required for each solute.^18^ For 189 wet octanol–water data points, the rmsd values obtained using *c* log *P* and Abraham's linear solvation energy relationship (LSER) are 0.8 kJ mol^−1^ and 1.1 kJ mol^−1^ respectively. Although the rmsd obtained using the model described here is slightly higher (1.6 kJ mol^−1^), the advantage is that extrapolation to a wider range of solutes and solvents requires minimal reparameterisation.

## Discussion

### SSIP representation of solutes

The representation of functional groups illustrated in Fig. 10 provides a straightforward method for determining the number of SSIPs required to describe a molecule based on assignment of an integer number of SSIPs to each heavy atom according to hybridisation and number of attached hydrogen atoms. Thus each alkane and alkene CH is assigned two SSIPs, one *α* and one *β*. Each sp^2^ hybridised carbon is assigned two *β* SSIPs that represent the two faces of the π-system. Each sp hybridised carbon is assigned four *β* SSIPs to represent the π-electrons. A similar approach is used for oxygen and nitrogen, but an additional *β* SSIP is assigned for each lone pair. The approach for halogens is slightly different as explained below.

Many of the required values of the *α* and *β* parameters were available from experimental measurements of association constants for 1 : 1 H-bonded complexes in carbon tetrachloride, and these parameters were used directly. For alkanes, which do not form H-bonds in carbon tetrachloride, experimental 1 : 1 complexation data in alkane solvents have been used to determine solvent H-bond parameters, and these parameters were used for alkane solutes.

**Fig. 16:**
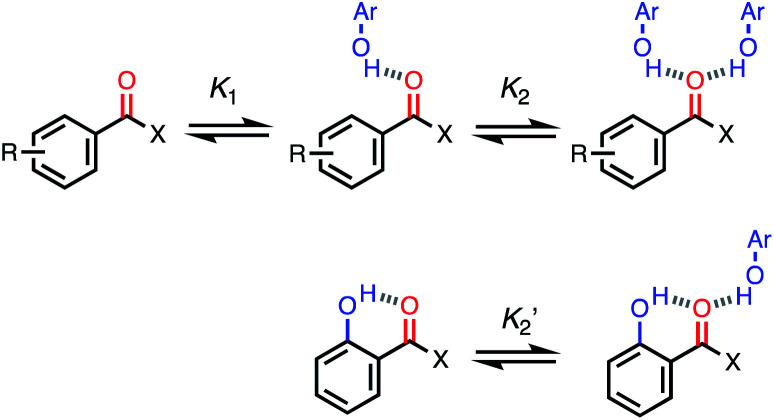
Sequential equilibria for formation of a 1 : 1 and 2 : 1 complex between a carbonyl H-bond acceptor and a phenol H-bond donor (R = H, 2-OMe or 4-OMe, X = H, Me, Ph or OMe). The equilibrium constant 
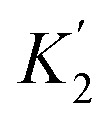
 for formation of a 1 : 1 complex with a carbonyl H-bond acceptor that has an intramolecular H-bond can be used to estimate the value of the second *β* SSIP required to describe solvation of a carbonyl oxygen.

For functional groups that have more than one possible H-bond acceptor site, such as alcohols, the experimental H-bond parameter was used for the first *β* SSIP, and value for the second *β* SSIP was optimised using partition data. In these cases, the optimised value of the second SSIP was always less polar than the experimental value used for the first SSIP. These results are consistent with a range of computational and experimental studies.^34–40^ Analysis of the H-bond acceptor parameters for carbonyl groups that make an intramolecular H-bond confirms the accuracy of the SSIP values. Fig. 16 shows that the equilibrium constant 
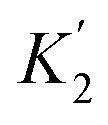
 is a surrogate for *K*_2_, the equilibrium constant for formation of a second intermolecular H-bond with a carbonyl group. The experimental values of 
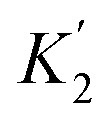
 correspond to *β* values of 3.8–4.3, which are significantly lower than the H-bond parameters measured for the first H-bond using *K*_1_ (5.5–6.4).^37^ These parameters agree well with the two different *β* values (3.8 and 5.8) used to describe the two lone pairs of a carbonyl group in Fig. 10.

### Functional group parameterisation

Parameters have been derived for the most common organic functional groups in this paper, but we have not discussed halogens so far. Chloroalkanes exemplify how the general approach can be extended to other functional groups. Eqn (9) indicates that the number of SSIPs required to describe a chlorine substituent is 5, which gives [SSIP] = 219 M for CH_2_Cl_2_, 212 M for CHCl_3_, 227 M for CH_2_Cl_2_CH_2_Cl_2_ and 207 M for CCl_4_. Experimental data on the association constants for 1 : 1 complexes of chloroalkanes and H-bond donors such as phenols suggest that one of these SSIPs has a *β* value of 2.3.^23^ However chlorine substituents are non-polar, so the other four SSIPs were assigned the same parameters as for alkanes, two with *α* = 1.2 and two with *β* = 0.6. Partition of *t*-butyl chloride between water and 10 organic solvents is well predicted using these values (rmsd = 1.1 kJ mol^−1^), which indicates that this SSIP representation provides a reasonable description of chlorine.

**Fig. 17:**
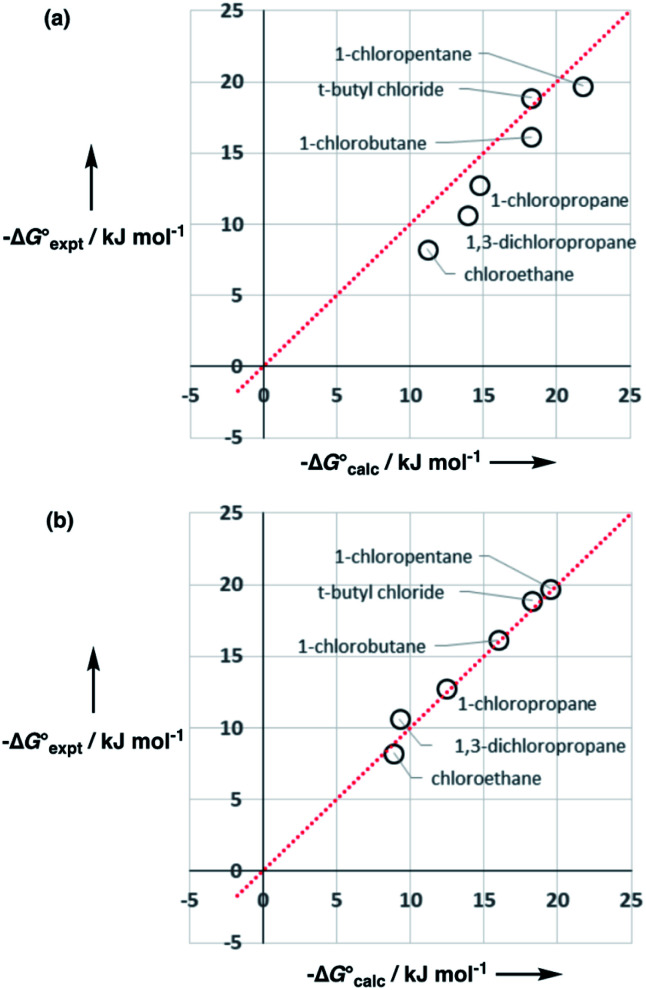
Comparison of calculated 
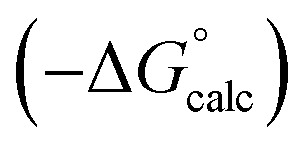
 and experimental free energies 
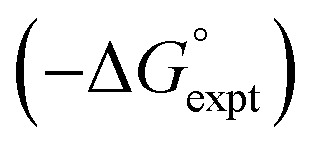
 for transfer of chloroalkane solutes from *n*-hexane into water. (a) Calculated values obtained using *α* = 1.2 for the C–H groups in CH_2_Cl (rmsd = 2.3 kJ mol^−1^). (b) Calculated values obtained using *α* = 1.6 for the C–H groups in –CH_2_Cl (rmsd = 0.7 kJ mol^−1^). The line is *y* = *x* in each case.

However, the agreement between calculated and experimental free energies of transfer for chloroalkanes which have CH_2_Cl groups was not so good (rmsd = 1.6 kJ mol^−1^ for 10 organic solvents). We have modelled alkyl substituents in various compound classes discussed above using the same parameters as for alkanes. Polarisation of C–H bonds adjacent to heteroatoms might be expected, but reasonable results were obtained without modifying the SSIPs in most cases. As the large values of *α* found for dichloromethane and chloroform might suggest, this is not the case for chloroalkanes. Fig. 17 shows that increasing the value of *α* for the C–H groups adjacent to the chlorine from 1.2 to 1.6 significantly improved the description of these compounds (rmsd = 0.9 kJ mol^−1^ for 10 organic solvents). Note that one of the assumptions implicit in the approach described here is that there are no changes in the net van der Waals interaction in the equilibrium illustrated in Fig. 1, so that partition can be described purely in terms of polar interactions between SSIPs. The excellent agreement between the calculated and experimental partition data obtained for chloroalkanes suggests that this assumption holds for second row elements as well.

### SSIP representation of solvents

For non-polar solvents, interactions with solutes can be described using just two types of solvent SSIP, *α*_S_ and *β*_S_. These parameters are available for several solvents from experimental measurements of association constants for 1 : 1 H-bonded complexes, and these values were used directly. However, most polar solvents require additional SSIPs in order to account for differences in the way they interact with polar and non-polar functional groups. For these solvents, the two SSIPs used to describe alkanes were used to describe the non-polar regions of the solvent, and two SSIPs were used to describe the polar regions of the solvent. For ethers, ketones and nitriles, solvation of polar solutes can be successfully described by using the relevant functional group H-bond acceptor parameters. For alcohols, self-association increases the apparent polarity of the solvent, so the hydroxyl groups were described using experimentally determined solvent parameters. Experimental solvent parameters are not available for the other strongly self-associated solvent, water, so the values of *α*_S_ and *β*_S_ were optimised using partition data. These optimised solvent parameters suggest that water interacts more strongly with H-bond acceptors than alcohols (*α*_S_ = 3.8 for water compared with *α*_S2_ = 3.5 for alcohols) but less strongly with H-bond donors (*α*_S_ = 3.5 for water compared with *α*_S2_ = 6.9 for alcohols). The difference between the description of water and alcohols is consistent with the Taft solvent parameters determined using solvatochromic dyes: the Taft H-bond donor parameter is larger for water (1.2 compared with 0.8 for alcohols), and the Taft H-bond acceptor parameter is smaller (0.5 compared with 0.8 for alcohols).^20^

Each solvent SSIP is also assigned a constant (*C*_*α*_ or *C*_*β*_), which quantifies the interactions that are broken with the bulk solvent when the solvent SSIP solvates a solute. The values of these constants were optimised using partition data, and they capture information about both the polarity and the concentration of solvent–solvent interactions. For solvents with just two types of SSIP, the sum of the two constants can be directly related to the properties of the solvent–solvent interactions by eqn (17).

17 *C*_*α*_ + *C*_*β*_ = −*α*_S_*β*_S_ + *RT* ln[S·S]

As explained above, the effective concentration of solvent–solvent interactions [S·S] is not easy to estimate for most solvents. When eqn (1) was first proposed the constant of +6 kJ mol^−1^ was obtained from measurements of formation of 1 : 1 H-bonded complexes in carbon tetrachloride.^19^ This value corresponds to [S·S] = 10 M, and we have assumed that a constant of +6 kJ mol^−1^ can also be used in eqn (1) to describe 1 : 1 complexation other organic solvents. However, the values of *C*_*α*_ and *C*_*β*_ obtained here from the partition data can be used in eqn (17) to obtain a direct experimental measurement of this parameter. The results shown in Table 4 confirm that the values of *RT* ln[S·S] for non-polar solvents are approximately constant (+5.5 to +6.6 kJ mol^−1^). The high value for chloroform (6.6 kJ mol^−1^) is indicative of the greater solvating power of a more polar solvent, and the low value for perfluoroalkanes (5.5 kJ mol^−1^) reflects the very weak intermolecular interactions present in these solvents. The results in Table 4 show why using a value of +6 kJ mol^−1^ for the constant in eqn (1) provides an accurate description of the association constants for formation of 1 : 1 complexes in different non-polar solvents. In contrast, partition is much more sensitive to the precise values of the solvent constants, because the small differences in Table 4 are significant when they are multiplied up by the total number of SSIPs in a solute.

**Table 4:** Values of *RT* ln[S·S] (kJ mol^−1^)^a^

Solvent	*RT* ln[S·S]
Hexadecane	6.0
Benzene	6.4
Toluene	6.4
Hexane	6.0
Cyclohexane	5.9
Carbon tetrachloride	6.0
Dichloromethane	6.4
Chloroform	6.6
1,2-Dichloroethane	6.4
Chlorobenzene	6.2
Perfluoroalkane	5.5

a Calculated using eqn (17).

The behaviour of polar solvents is more complicated, because with two types of donor and two types of acceptor there are four different types of solvent–solvent interaction. However in the case of alcohols, the solvent parameters in Table 3 show that *C*_*α*1_ ≈ *C*_*β*1_ and *C*_*α*2_ ≈ *C*_*β*2_. Structural studies on alcohols showing clustering of the alkyl chains and separate H-bonded aggregates of the hydroxyl groups.^41,42^ In other words to a first approximation, we can think of alcohols as consisting of two independent solvating domains. The solvent parameters that describe the non-polar hydrocarbon domain in alcohols are similar to those found for alkanes. Using the values of *α*_S1_, *C*_*α*1_, *β*_S1_ and *C*_*β*1_ in eqn (17) gives *RT* ln[S·S] = +6.4 kJ mol^−1^ for the hydrocarbon domain in alcohols, which is similar to the values found for non-polar solvents in Table 4. The polar hydroxyl domain is described by *α*_S2_, *C*_*α*2_, *β*_S2_ and *C*_*β*2_, and using these values in eqn (17) gives a value of +11.7 kJ mol^−1^ for *RT* ln[S·S], which is the same as the value for water in Table 4. The model that emerges from the partition data is that alcohol solvents behave as a mixture of a water-like domain and an alkane-like domain.

There is an additional solvent constant *C*_0_, which is used to describe differences between solvents that are not accounted for by the SSIP interaction model. The values of *C*_0_ listed in Tables 2 and 3 are all positive and small (generally less than +2 kJ mol^−1^), and there are no obvious patterns. This result indicates that a simple model based on pairwise interactions between specific interaction sites on solvent and solute provides a rather general description of solvation phenomena and that there are no major additional factors that need to be considered.

### The hydrophobic effect

Although the approach was developed from experiments on H-bonded complexes in non-polar solvents, the model provides a good description of very different phenomena, such as the hydrophobic effect in water. Interestingly, there is no need for different treatments of the water and hydrocarbon solvents or the need for a special cavitation term to describe the breaking of water structure. Free energy of transfer of a non-polar molecule from a non-polar liquid into water is a process dominated by disruption of water–water interactions and has been quantitatively linked to the molecular volume or molecular surface area of the solute.^43^ It has been estimated that the free energy of transfer from a non-polar liquid into water is unfavourable by 0.2 kJ mol^−1^ Å^−2^ of non-polar molecular surface area, but calculation of non-polar surface area requires specialist software.^44^ The total number of SSIPs that are assigned to a molecule using the relatively simple procedure outlined above is closely correlated to the molecular surface area for diverse structural type and functionality (see ESI Section S8†), so the magnitude of the hydrophobic effect can be quite accurately estimated. The advantage of this SSIP approach is that it is a simple spreadsheet calculation.

Consider for example transfer of the 9 different alkanes in Table 5 from *n*-hexadecane into water. Each of these alkanes has 16 hydrogen atoms, and each C–H groups has two SSIPs with values of *α* = 1.2 and *β* = 0.6. The free energy of solvation for each of the SSIPs can be calculated using eqn (4) and (5) with the solvent constants listed in Table 2.

**Table 5:** Experimental free energies of transfer for alkane solutes at 298 K

Alkane	Formula	*n*-Hexadecane to water	*n*-Hexadecane to gas	Melting point
		Δ*G*°/kJ mol^−1^	Δ*G*°/kJ mol^−1^	K
*n*-Heptane	C_7_H_16_	29.3	18.1	182
3-Methylhexane	C_7_H_16_	28.7	17.4	154
2,2-Dimethylpentane	C_7_H_16_	28.0	16.0	149
Ethylcyclohexane	C_8_H_16_	31.1	22.1	162
Propylcyclopentane	C_8_H_16_	30.6	21.7	156
*cis*-1,2-Dimethylcyclohexane	C_8_H_16_	28.6	21.9	223
*trans*-1,4-Dimethylcyclohexane	C_8_H_16_	28.7	20.8	236
Cyclooctane	C_8_H_16_	28.3	24.7	288
Adamantane	C_10_H_16_	27.5	28.1	543

For water:Δ*g*_S_(*α*)/kJ mol^−1^ = −*αβ*_S_ − *C*_*β*_ = −1.20 × 3.47 + 0.76 = −3.40Δ*g*_S_(*β*)/kJ mol^−1^ = −*α*_S_*β* − *C*_*α*_ = −3.80 × 0.60 + 0.76 = −1.52

For *n*-hexadecane:Δ*g*_S_(*α*)/kJ mol^−1^ = −*αβ*_S_ − *C*_*β*_ = −1.20 × 0.60 − 2.64 = −3.36Δ*g*_S_(*β*)/kJ mol^−1^ = −*α*_S_*β* − *C*_*α*_ = −0.60 *×* 1.20 − 2.64 = −3.36

The difference between these solvation energies is (−3.40–1.52) − (−3.36–3.36) = 1.80 kJ mol^−1^, which represents the free energy of transfer of one C–H group from *n*-hexadecane to water. Thus the calculated value for the free energy of transfer is the same for all of the alkanes, 16 × 1.8 = 28.8 kJ mol^−1^, which agrees very well with the experimental values in Table 5 (29 ± 2 kJ mol^−1^).

### Phase change equilibria

The behaviour of alkanes with respect to liquid–liquid transfer is remarkably predictable, but this is not the case for gas–liquid and solid–liquid equilibria. Table 5 also shows experimental values for the free energy of transfer of C_*x*_H_16_ alkanes from *n*-hexadecane into the gas phase. These values vary significantly from one alkane to another and span range of more than 10 kJ mol^−1^. It is clear that there is an additional contribution to the free energy of transfer of a molecule into the gas phase that is not included in the liquid phase solvation model described here. The origin of this difference is not known, but cyclic structures appear to have a dramatic effect, and in order to obtain an accurate model of gas–liquid equilibria, most methods require an empirical correction based on experimentally determined vapour pressures. Table 5 also shows that there is a variation of 400 K in the melting points of the 9 different alkanes, which highlights the dramatic difference between these phase change equilibria and liquid–liquid transfer. The equations in this paper calculate the free energy of a solute in a solvent relative to a liquid-like reference state, and additional contributions from changes in internal molecular motion are likely to be significant when a phase change is involved.

### The fluorous effect

Fluorous solvents are an important class of solvents that exhibit a range of anomalous properties compared with other liquids. Perfluorocarbons are more volatile that the corresponding hydrocarbons, dissolve very high concentrations of gaseous solutes, and do not mix with either non-polar or polar solvents. The preferential dissolution of perfluorocarbon solutes in perfluorocarbon solvents is known as the fluorous effect and has been exploited in separation technology. As indicated in Table 4, the low value of *RT* ln[S·S] provides a good description of the weak intermolecular interactions present in perfluorocarbon solvents for most solutes. However, the model breaks down for perfluorocarbon solutes. The free energy of transfer of perfluorocarbon solutes (C_5_F_12_ to C_8_F_18_) from alkanes to water has a slightly higher error than for other solutes (rmsd = 4.8 kJ mol^−1^, *n* = 4), but the errors are significant for the transfer from perfluorocarbon solvents to alkanes (rmsd = 13.8 kJ mol^−1^) and to water (rmsd = 18.6 kJ mol^−1^). The strength of the interactions between perfluorocarbon solutes and perfluorocarbon solvents is consistently underestimated by the model. The origin of this special interaction between perfluorocarbons is difficult to understand, especially given the volatility of these substances, which indicates weak intermolecular interactions. Steric effects that interfere with molecular packing may contribute to this anomaly, and considerable changes in volume occur when hydrogenated and fluorinated substances are mixed.^45^ For example, when *n*-perfluorohexane is dissolved in *n*-octane at infinite dilution, the molar volume of the solute increases by 13%.^46,47^ The effect is even more pronounced when *n*-alkanes are dissolved in *n*-perfluoroalkanes, where the molar volume of the solute increases by 20%.^48^ It is as though layers of empty space are created around each molecule, and this behaviour clearly cannot be captured by a simple model that assumes van der Waals interactions balance and polar interactions dominate. In conclusion, the fluorous effect is outside the scope of the model developed in this paper even though the model provides a good description of the solvation of different types of solute by perfluorocarbon solvents.

## Conclusions

Solvation of organic molecules is a fundamentally important property that governs intermolecular association of solutes and phase transfer equilibria. Both processes depend on non-covalent interactions between solutes and solvents, and we have shown that it is possible to quantitatively account for the free energy changes associated with intermolecular contacts by using surface site interaction points (SSIP). A molecule is described by a discrete set of SSIPs, which define the array of all possible interactions that can be made with other molecules. Association constants for the formation of 1 : 1 complexes in solution are governed by the interaction of the most polar SSIP on each solute, whereas phase transfer equilibria are governed by the sum of the interactions made by all of the solute SSIPs with the solvent. A large body of experimental data is available for both processes, and here we use this information to parameterise an integrated model for describing non-covalent interactions and solvation.

Solutes are described based on the composition of functional groups, which allows straightforward rule-based translation of chemical structure into a SSIP description without the need for the *ab initio* calculations we have used previously. Positive SSIPs (*α*) are assigned to hydrogen atoms, and negative SSIPs (*β*) are assigned to represent lone pairs and π-electron density. The total number of SSIPs used to represent a solute is fixed such that the concentration of SSIPs in a liquid is approximately constant. The assumption is that van der Waals contributions cancel out in any association or phase transfer equilibria, so that the behaviour of the system is dominated by polar interactions between SSIPs. Equilibria are treated as a competition between pairwise interactions of solute and solvent SSIPs, which has been shown previously to provide a rather good description of experimental data on 1 : 1 complexation in organic solvents.

Values of the most polar SSIPs required to describe each functional group were obtained from the experimentally determined H-bond parameters *α* and *β*. The values of any additional SSIPs required to complete description of each functional group were obtained by optimisation using experimental data on phase transfer equilibria. Similarly, the values of the SSIPs required to describe many organic solvents (*α*_S_ and *β*_S_) have been determined previously from experimental data on 1 : 1 complexation. The SSIP description of other solvents like water, where 1 : 1 complexes are not sufficiently stable for experimental study, were obtained by optimisation using experimental data on phase transfer equilibria. For solvents that contain both polar and non-polar functional groups like alcohols, two sets of solvent SSIPs were used to describe the equilibrium between the two different solvation modes. In addition, for each solvent SSIP, a constant term was used to describe the effects of solvent–solvent interactions. The resulting model is simple to implement using just a spreadsheet (see ESI Section S9†) and accurately describes the transfer of a wide range of different solutes from water to a wide range of different organic solvents (overall rmsd is 1.4 kJ mol^−1^ for 1713 data points).

The model described above is the result of a feasibility study based on solutes with one functional group and a limited set of solvents. The purpose was to determine whether the solvent competition model originally applied to H-bond complexes in non-polar solvents could be extended to describe whole molecule solvation and partition between organic solvents and water. This simple model describes the hydrophobic effect with surprising accuracy. It has also been possible to deduce new descriptors for range of organic solvents that were not accessible by direct investigation of H-bond formation in non-polar solvents. These empirical parameters should be of value in linking the results of *ab initio* quantum mechanics calculations with the thermodynamic properties of molecules in solution.

Experimental H-bond parameters are available for most organic functional groups, and these parameters can be used to develop the method for application to a much wider range of solutes than those used in the parameterisation described here. In addition, the availability of experimental H-bond parameters for anionic and cationic functional groups may provide a method for the treatment of ionisable compounds. One limitation is that the experimental data on 1 : 1 complexation and partition is generally restricted to room temperature, which means that extrapolation to different temperatures would require a new treatment. There are some challenges that will have to be addressed in development of the model to tackle more complex polyfunctional compounds. Electronic interactions between functional groups through the bonding framework can affect the H-bond parameters, and intramolecular non-covalent interactions may change the availability of SSIPs for interaction with solvent. In addition, the current model is based purely on covalent connectivity, and a more elaborate treatment would be required to tackle the effects of conformer distribution on solvation energies.

## Methods

### Experimental partition data

The initial training set of neutral organic solutes included a variety of common functional groups and various structural types. The selected solutes had published partition coefficients available for several different solvents. Gas-*n*-hexadecane partition coefficients were available for the whole set and gas-water partition for 219 molecules. Thermodynamic values for solvent–solvent partition were calculated from the available values of the free energy of transfer from gas to solvent (see ESI Section S7† for values and references to data sources).

### Analysis of chemical structures

From the SMILES string each molecule was fragmented into constituent atom types.^49^ Each non-hydrogen atom was assigned a group code based on SMARTS nomenclature that described the atom and the directly bonded neighbours. Each atom code was assigned a number of donor and acceptor SSIP. In the optimisation process, the number of SSIP per atom was constrained. Only integer numbers of SSIP per atom were allowed, and a close correlation between the total number of SSIP used to describe a molecule and the molecular volume was maintained. Aromatic groups were assigned an additional code to describe a SSIP in the centre of the π-face of each 6 membered ring.

### Calculation procedure

For each SSIP, the free energies of interaction with solvent were calculated using eqn (4) and (5) for simple solvents or eqn (11)–(16) for complex solvents. A lookup table was used to retrieve the free energy calculated for each code, and these values were summed as in eqn (7) to calculate the overall transfer free energy from the reference phase. Free energy of transfer between solvents was calculated with eqn (8) and compared with experimental values. The calculations were performed with Microsoft Excel, and the matrix of compounds and atom codes required for the calculations was assembled by analysis of SMILES strings using a combination of the fragmentation tools in Advanced Algorithm Builder software and the open source CDK tools accessible within Excel through the LICSS add-in.^33,50,51^

### Optimisation of parameters

Published values of functional group H-bond parameters derived from experimental data were used as a starting point.^19,23,24^ An iterative approach was used to assign new *α* and *β* values and new solvent constants by minimisation of the root mean square deviation (rmsd) between calculated and experimental values. A stepwise approach was adopted starting with a small initial training set of hydrocarbon solutes from which it was possible to deduce descriptors for the water model based on partition data for the systems hexadecane/water and benzene/water. With parameters for water established it was a straightforward process to refine parameters for other solvents and to assign *α* and *β* values to interaction sites for which there was no reliable precedent.

## Data availability

All supporting data is provided in the ESI.†

## Author contributions

All authors contributed to writing the manuscript.

## Conflicts of interest

There are no conflicts to declare.

## Supplementary Material

SC-012-D1SC03392A-s001

SC-012-D1SC03392A-s002

SC-012-D1SC03392A-s003

## References

[cit1] Constantinescu D., Gmehling J. (2016). J. Chem. Eng. Data.

[cit2] Fredenslund A., Vapor-liquid equilibria using UNIFAC: a group-contribution method, Elsevier, 2012

[cit3] Fredenslund A., Jones R. L., Prausnitz J. M. (1975). AIChE J..

[cit4] Lohmann J., Joh R., Gmehling J. (2001). Ind. Eng. Chem. Res..

[cit5] Qiu D., Shenkin P. S., Hollinger F. P., Still W. C. (1997). J. Phys. Chem. A.

[cit6] Duffy E. M., Jorgensen W. L. (2000). J. Am. Chem. Soc..

[cit7] Cramer C. J., Truhlar D. G. (2008). Acc. Chem. Res..

[cit8] Rivail J.-L. and Rinaldi D., Computational Chemistry, Review of Current Trends, World Scientific, New York, 1996, p. 139

[cit9] Tomasi J., Mennucci B., Cammi R. (2005). Chem. Rev..

[cit10] Pomogaeva A., Chipman D. M. (2013). J. Phys. Chem. A.

[cit11] Andreussi O., Dabo I., Marzari N. (2012). J. Chem. Phys..

[cit12] Hille C., Ringe S., Deimel M., Kunkel C., Acree W. E., Reuter K., Oberhofer H. (2019). J. Chem. Phys..

[cit13] Eckert F., Klamt A. (2002). AIChE J..

[cit14] Klamt A., Eckert F. (2000). Fluid Phase Equilib..

[cit15] Klamt A. (2018). Wiley Interdiscip. Rev.: Comput. Mol. Sci..

[cit16] Abraham M. H. (1993). Chem. Soc. Rev..

[cit17] Abraham M. H., Ibrahim A., Zissimos A. M., Zhao Y. H., Comer J., Reynolds D. P. (2002). Drug Discovery Today.

[cit18] Abraham M. H., Acree Jr W. E. (2010). J. Org. Chem..

[cit19] Hunter C. A. (2004). Angew. Chem., Int. Ed. Engl..

[cit20] Cabot R., Hunter C. A. (2012). Chem. Soc. Rev..

[cit21] Hunter C. A. (2013). Chem. Sci..

[cit22] Oliver A., Hunter C. A., Prohens R., Rosselló J. L. (2018). J. Comput. Chem..

[cit23] Calero C. S., Farwer J., Gardiner E. J., Hunter C. A., Mackey M., Scuderi S., Thompson S., Vinter J. G. (2013). Phys. Chem. Chem. Phys..

[cit24] Musumeci D., Hunter C. A., Prohens R., Scuderi S., McCabe J. F. (2011). Chem. Sci..

[cit25] Driver M. D., Williamson M. J., Cook J. L., Hunter C. A. (2020). Chem. Sci..

[cit26] Buurma N. J., Cook J. L., Hunter C. A., Low C. M. R., Vinter J. G. (2010). Chem. Sci..

[cit27] Amenta V., Cook J. L., Hunter C. A., Low C. M. R., Vinter J. G. (2011). Org. Biomol. Chem..

[cit28] Amenta V., Cook J. L., Hunter C. A., Low C. M. R., Vinter J. G. (2012). J. Phys. Chem. B.

[cit29] Cabot R., Hunter C. A., Varley L. M. (2010). Org. Biomol. Chem..

[cit30] Gomez S., Rojas-Valencia N., Gomez S. A., Cappelli C., Merino G., Restrepo A. (2021). Chem. Sci..

[cit31] Pay N. G. M., Symons M. C. R. (1993). J. Chem. Soc., Faraday Trans..

[cit32] Henkel S., Misuraca M. C., Troselj P., Davidson J., Hunter C. A. (2018). Chem. Sci..

[cit33] Japertas P., Didziapetris R., Petrauskas A. (2002). Quant. Struct.-Act. Relat..

[cit34] Symons M. C. R. (1986). Pure Appl. Chem..

[cit35] Eaton G., Pena-Nunez A. S., Symons M. C. R. (1988). J. Chem. Soc., Faraday Trans..

[cit36] Eaton G., Symons M. C. R., Rastogi P. P., O'Duinn C., Waghorne W. E. (1992). J. Chem. Soc., Faraday Trans..

[cit37] Berthelot M., Laurence C., Foucher D., Taft R. W. (1996). J. Phys. Org. Chem..

[cit38] Feldblum E. S., Arkin I. T. (2014). Proc. Natl. Acad. Sci. U. S. A..

[cit39] Yang J., Christianson L. A., Gellman S. H. (1999). Org. Lett..

[cit40] Yang J., Gellman S. H. (1998). J. Am. Chem. Soc..

[cit41] Böhmer R., Gainaru C., Richert R. (2014). Phys. Rep..

[cit42] Franks N. P., Abraham M. H., Lieb W. R. (1993). J. Pharm. Sci..

[cit43] Leo A., Hansch C., Jow P. Y. (1976). J. Med. Chem..

[cit44] Hunter C. A. (2013). Chem. Sci..

[cit45] Morgado P., Garcia A. R., Martins L. F. G., Ilharco L. M., Filipe E. J. M. (2017). Langmuir.

[cit46] Morgado P., Rodrigues H., Blas F. J., McCabe C., Filipe E. J. M. (2011). Fluid Phase Equilib..

[cit47] Morgado P., Tomás R., Zhao H., dos Ramos M. C., Blas F. J., McCabe C., Filipe E. J. M. (2007). J. Phys. Chem. C.

[cit48] Lepori L., Matteoli E., Spanedda A., Duce C., Tiné M. R. (2002). Fluid Phase Equilib..

[cit49] Weininger D. (1988). J. Chem. Inf. Comput. Sci.

[cit50] Willighagen E. L., Mayfield J. W., Alvarsson J., Berg A., Carlsson L., Jeliazkova N., Kuhn S., Pluskal T., Rojas-Chertó M., Spjuth O., Torrance G., Evelo C. T., Guha R., Steinbeck C. (2017). J. Cheminf..

[cit51] Lawson K. R., Lawson J. (2012). J. Cheminf..

