# Supplementary material for: An empirical model for solvation based on surface site interaction points
Source: Chem Sci. 2021 Sep 16;12(39):13193–208. doi: 10.1039/d1sc03392a (PMC8513935; doi:10.1039/d1sc03392a)
Supplement: SC-012-D1SC03392A-s001 [file SC-012-D1SC03392A-s001.pdf]

Electronic Supplementary Material (ESI) for Chemical Science.
This journal is © The Royal Society of Chemistry 2021

An Empirical Model for Solvation Based on Surface Site Interaction Points

Derek P. Reynolds,* Maria Chiara Storer, Christopher A. Hunter*

Yusuf Hamied Department of Chemistry, University of Cambridge, Lensfield Road, Cambridge CB2 1EW (UK)

Supplementary Information

Section	Title	Page
1	Comparison of free energies ($-\Delta G^0$ kJ mol^{-1}) of transfer of alkanes from gas phase into n-hexadecane with transfer into other solvents.	S2
2	Complex formation between aromatic acceptors and H-bond donors	S3
3	Substructure fragments	S6
4	Free energy of formation for H-bonded 1:1 complexes	S7
5	Individually optimised solvent constants	S37
6	Solvent H-bond parameters α_S and β_S	S38
7	Calculated and experimental free energies of transfer in workbook Excel1.xlsx	S40
8	Correlation between Molecular Surface Area and the number of Surface Site Interaction Points	S44
9	Description of calculation procedure exemplified in workbook Excel2.xlsx	S48

Section 1:

Comparison of free energies (-ΔG^0 kJ mol^{-1}) of transfer of alkanes from gas phase into n-hexadecane with transfer into other solvents.

Table S1: Free energies (-ΔG^0 kJ mol^{-1}) of transfer of alkanes from gas phase to solvent
(Solvents were only included if at least 10 transfer values were available)

	Hexadecane	CCl4	Hexane	Propionitrile	Tetrahydrofuran	Chlorobenzene	Acetone	Propanone	cyclohexanone	Methanol	Ethanol	propan-1-ol	Propan-2-ol
**Alkane solute**	**-ΔG^0**	**-ΔG^0**	**-ΔG^0**	**-ΔG^0**	**-ΔG^0**	**-ΔG^0**	**-ΔG^0**	**-ΔG^0**	**-ΔG^0**	**-ΔG^0**	**-ΔG^0**	**-ΔG^0**	**-ΔG^0**
n-pentane	12.3	13.5	13.9	11.0	13.3	13.9	11.3	11.8	11.3	9.5	10.2	10.3	10.6
n-hexane	15.2	17.0	16.9	13.4	16.2	16.8	13.5	14.4	13.9	11.7	12.8	13.0	13.1
n-heptane	18.1	19.9	20.0	16.1	19.1	19.8	16.0	17.3	16.8	14.3	15.5	15.6	15.6
n-octane	21.0	23.1	22.6	18.3	22.0	22.8	18.4	19.7	19.5	16.1	18.1	18.1	18.1
n-nonane	23.9	26.0	25.7	20.7	24.8	25.8	20.9	22.3	21.9	18.4	20.1	20.3	20.7
n-decane	26.7	#N/A	28.7	#N/A	#N/A	#N/A	#N/A	25.2	#N/A	#N/A	22.4	#N/A	#N/A
2-methylpentane	14.3	16.0	16.1	12.6	15.4	15.9	12.8	13.6	13.1	11.2	12.3	12.3	12.3
2,5-dimethylhexane	18.9	20.9	20.8	16.7	20.1	20.8	16.8	17.9	17.5	#N/A	#N/A	#N/A	16.4
2,2,4-trimethylpentane	17.7	#N/A	#N/A	#N/A	19.5	#N/A	#N/A	16.9	#N/A	#N/A	#N/A	15.3	15.4
2,3,4-trimethylpentane	19.9	21.2	21.0	17.2	20.5	21.1	17.3	18.4	18.0	15.2	16.8	16.8	16.8
3,3-diethylpentane	22.9	#N/A	24.6	#N/A	#N/A	#N/A	#N/A	#N/A	#N/A	18.0	#N/A	#N/A	#N/A
cyclopentane	14.1	#N/A	15.5	#N/A	#N/A	#N/A	#N/A	#N/A	#N/A	11.0	11.6	12.1	12.1
cyclohexane	16.9	18.4	17.7	15.2	17.9	18.4	14.8	15.7	16.0	13.9	14.5	14.5	14.4
cycloheptane	21.1	#N/A	#N/A	#N/A	#N/A	#N/A	#N/A	#N/A	#N/A	#N/A	#N/A	17.9	17.7
methylcyclopentane	16.6	#N/A	#N/A	#N/A	#N/A	#N/A	#N/A	#N/A	#N/A	#N/A	#N/A	#N/A	#N/A
methylcyclohexane	18.9	#N/A	#N/A	17.1	19.5	#N/A	16.4	17.2	#N/A	#N/A	#N/A	#N/A	15.9
ethylcyclohexane	22.1	23.3	22.6	19.1	22.4	23.2	18.9	20.1	20.2	16.9	18.6	18.7	18.7
methane	-1.8	-0.8	-0.1	#N/A	-1.0	-1.9	-1.3	#N/A	#N/A	-1.5	-1.7	-1.7	-1.9
ethane	2.8	4.2	4.3	#N/A	3.5	3.4	2.9	#N/A	2.2	2.2	2.5	2.6	2.7
propane	6.0	7.6	7.5	#N/A	#N/A	6.7	5.7	#N/A	#N/A	4.8	5.2	5.5	5.1
butane	9.2	11.2	10.7	#N/A	#N/A	9.7	8.9	9.6	#N/A	7.2	8.0	8.3	8.5
cyclopropane	7.5	#N/A	8.3	#N/A	#N/A	#N/A	#N/A	#N/A	#N/A	#N/A	#N/A	6.6	6.6
2,2-dimethylpropane	10.4	#N/A	11.6	#N/A	#N/A	#N/A	#N/A	#N/A	#N/A	8.0	#N/A	#N/A	#N/A

Figure S1: **Comparison of free energies (-ΔG^0 kJ mol^{-1}) of transfer of alkanes from gas phase into n-hexadecane with transfer into other solvents** (x axis**:** hexadecane and y axis: solvent2)

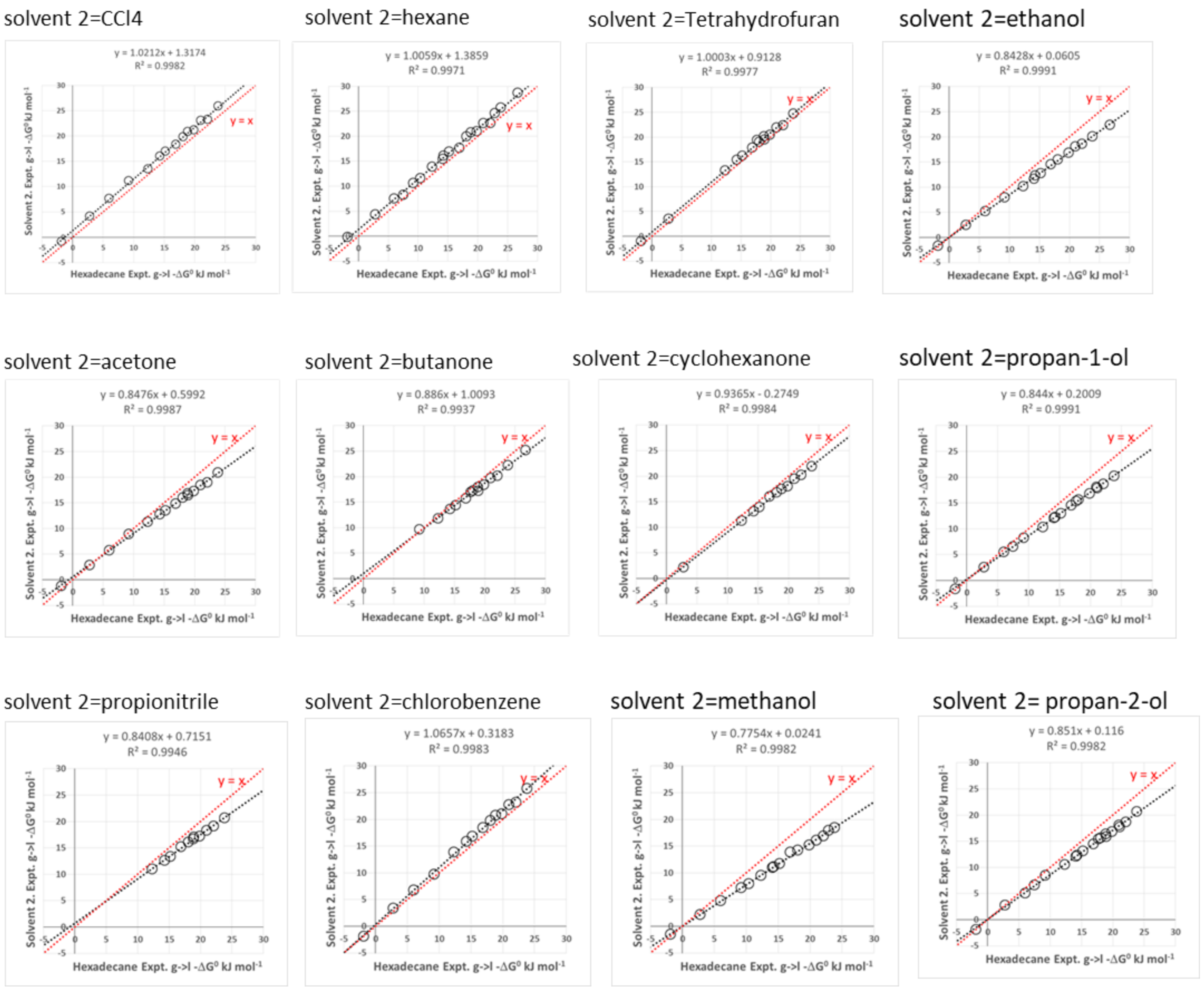

Section 2:
Complex formation between aromatic acceptors and H-bond donors

H-bond parameters for the polar interaction sites on the π-faces of aromatic hydrocarbons

Aromatic acceptors	β
benzene	2.00
toluene	2.20
ortho-, meta- or para-xylene	2.40
mesitylene	2.70
hexamethylbenzene	3.10

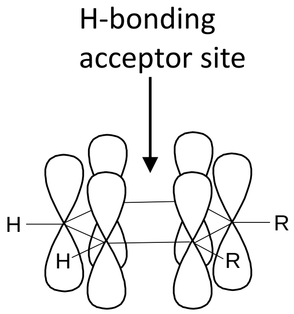

Solvent parameters for CCl_4 from ref [1] and alpha values for donors from ref [2]

$$\mathrm{CCl_4}\ \text{solvent: } \alpha_S = 1.4 \text{ and } \beta_S = 0.6$$

$$\text{Calculated } -\Delta G^0_{CCl_4}\,(\mathrm{kJ\ mol^{-1}}) = (\alpha - 1.4)(\beta - 0.6) - 6$$

Figure S2:
Calculated v experimental $-\Delta G^0$ for formation of 1:1 complexes between benzene acceptors and alcohol and phenol H-bond donors in CCl_4 (rmsd= 0.35 kJ mol^{-1} n= 38)

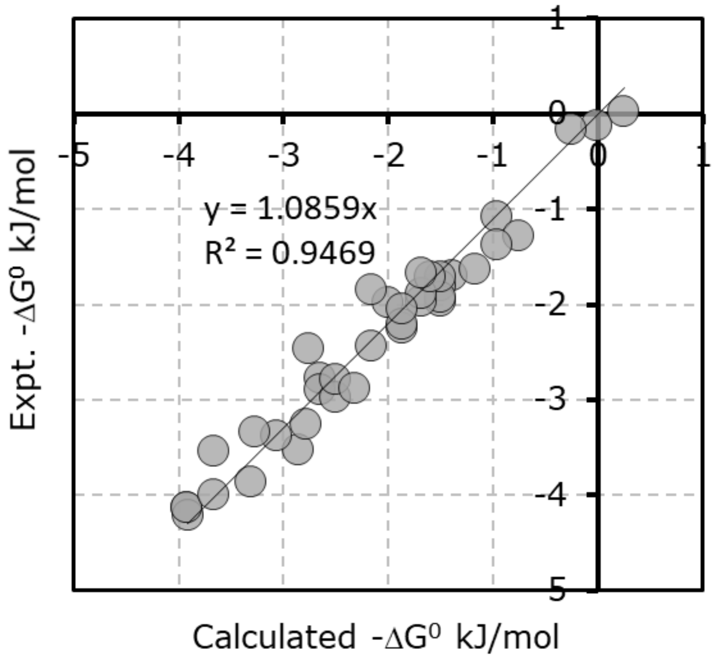

.

Table S2

Calculated v experimental $-\Delta G^0$ for formation of 1:1 complexes between benzene acceptors and alcohol and phenol H-bond donors in CCl_4

H-Bond Donor	α	H-Bond Acceptor	β	$-\Delta G^0$ Calculated kJ mol^{-1}	$-\Delta G^0$ Experiment kJ mol^{-1}	Reference
Methanol	**2.90**	benzene	**2.00**	-3.90	-4.21*	[3]
2-Methylphenol	**3.50**	benzene	**2.00**	-3.06	-3.39	[4]
4-methylphenol	**3.70**	benzene	**2.00**	-2.78	-3.26	[4]
phenol	**3.80**	benzene	**2.00**	-2.64	-2.77	[4]
1-naphthol	**3.80**	benzene	**2.00**	-2.64	-2.90	[4]
2-naphthol	**3.90**	benzene	**2.00**	-2.50	-2.98	[4]
4-fluorophenol	**3.90**	benzene	**2.00**	-2.50	-2.80	[5]
4-nitrophenol	**4.70**	benzene	**2.00**	-1.38	-1.70	[4]
tert-Butyl alcohol	**2.70**	toluene	**2.20**	-3.92	-4.13	[6]
4-methylphenol	**3.70**	toluene	**2.20**	-2.32	-2.89	[4]
1-naphthol	**3.80**	toluene	**2.20**	-2.16	-2.44	[4]
phenol	**3.80**	toluene	**2.20**	-2.16	-1.86	[4]
4-fluorophenol	**3.90**	toluene	**2.20**	-2.00	-1.98	[5]
4-methylphenol	**3.70**	o-xylene	**2.40**	-1.86	-2.22	[4]
phenol	**3.80**	o-xylene	**2.40**	-1.68	-1.97	[4]
2-naphthol	**3.90**	o-xylene	**2.40**	-1.50	-1.93	[4]
tert-Butyl alcohol	**2.70**	m-xylene	**2.40**	-3.66	-3.55	[6]
Methanol	**2.90**	m-xylene	**2.40**	-3.30	-3.87	[6]
4-methylphenol	**3.70**	m-xylene	**2.40**	-1.86	-2.26	[4]
2-naphthol	**3.90**	m-xylene	**2.40**	-1.50	-1.97	[4]
tert-Butyl alcohol	**2.70**	p-xylene	**2.40**	-3.66	-4.00	[6]
4-methylphenol	**3.70**	p-xylene	**2.40**	-1.86	-2.05	[4]
phenol	**3.80**	p-xylene	**2.40**	-1.68	-1.89	[4]
1-naphthol	**3.80**	p-xylene	**2.40**	-1.68	-1.68	[4]
2-naphthol	**3.90**	p-xylene	**2.40**	-1.50	-1.81	[4]
4-fluorophenol	**3.90**	p-xylene	**2.40**	-1.50	-1.71	[7]
tert-Butyl alcohol	**2.70**	mesitylene	**2.70**	-3.27	-3.35	[6]
Methanol	**2.90**	mesitylene	**2.70**	-2.85	-3.54	[6]
2-Methylphenol	**3.50**	mesitylene	**2.70**	-1.59	-1.72	[4]
4-methylphenol	**3.70**	mesitylene	**2.70**	-1.17	-1.63	[4]
1-naphthol	**3.80**	mesitylene	**2.70**	-0.96	-1.09	[4]
phenol	**3.80**	mesitylene	**2.70**	-0.96	-1.39	[4]
4-fluorophenol	**3.90**	mesitylene	**2.70**	-0.75	-1.28	[5]
tert-Butyl alcohol	**2.70**	Hexamethylbenzene	**3.10**	-2.75	-2.47	[6]
4-methylphenol	**3.70**	Hexamethylbenzene	**3.10**	-0.25	-0.17	[4]
phenol	**3.80**	Hexamethylbenzene	**3.10**	0.00	-0.13	[4]
4-fluorophenol	**3.90**	Hexamethylbenzene	**3.10**	0.25	0.02	[5]

Footnote to table: * reported value converted from mole fraction standard state to molar standard state

References for Section 2

1. Hunter, C.A., *Quantifying intermolecular interactions: guidelines for the molecular recognition toolbox.* Angew Chem Int Ed Engl, 2004. **43**(40): p. 5310-24.
2. Calero, C.S., et al., *Footprinting molecular electrostatic potential surfaces for calculation of solvation energies.* Phys Chem Chem Phys, 2013. **15**(41): p. 18262-73.
3. Koné, M., et al., *Can Quantum-Mechanical Calculations Yield Reasonable Estimates of Hydrogen-Bonding Acceptor Strength? The Case of Hydrogen-Bonded Complexes of Methanol.* The Journal of Physical Chemistry A, 2011. **115**(47): p. 13975-13985.
4. Joesten, M.D. and L.J. Schaad, *Hydrogen Bonding*. 1974: Marcel Dekker, New York.
5. Laurence, C., et al., *The pK(BHX) database: toward a better understanding of hydrogen-bond basicity for medicinal chemists.* J Med Chem, 2009. **52**(14): p. 4073-86.
6. Murthy, A.S.N. and C.N.R. Rao, *Spectroscopic Studies of the Hydrogen Bond.* Applied Spectroscopy Reviews, 1968. **2**(1): p. 69-191.
7. Laurence, C. and M. Berthelot, *Observations on the strength of hydrogen bonding.* Perspectives in Drug Discovery and Design, 2000. **18**(1): p. 39-60.

Section 3:

Substructure fragments

For each molecule the SMILES string was analysed and a SMARTS based substructure code from table S3 was assigned to each heavy atom. Aromatic groups were assigned an additional code to describe a SSIP in the centre of the π-face of each aromatic 6 membered ring.

Table S3: Description of the substructure fragments used to describe the solutes and details of the number SSIPs and the associated parameters assigned to each fragment

Fragment ID	Description	Central Atom Type	SMARTS based code	no of α_1 SSIP	α_1	no of α_2 SSIP	α_2	no of β_1 SSIP	β_1	no of β_2 SSIP	β_2
1	H attached to sp3 C also bonded to one F atom	Csp3	[H]CF	**1**	1.70	**0**	0.00	**1**	0.20	**0**	0.00
2	H attached to sp3 C also bonded to one Cl atom	Csp3	[H]C(Cl)	**1**	1.60	**0**	0.00	**1**	0.60	**0**	0.00
3	H attached to sp3 C	Csp3	[H]C	**1**	1.20	**0**	0.00	**1**	0.60	**0**	0.00
4	sp2 C with H attached	Csp2 aliphatic	[H][C](C)=C	**1**	1.20	**0**	0.00	**2**	1.31	**1**	0.60
5	sp2 C with two H attached	Csp2 aliphatic	[H][C]([H])=C	**2**	1.20	**0**	0.00	**2**	1.31	**2**	0.60
6	H attached to sp2 C in a formamide	Csp2 aliphatic	[H][C](N)=O	**1**	1.20	**0**	0.00	**1**	0.60	**0**	0.00
7	sp2 C with no attached H	Csp2 aliphatic	C[C](C)=C	**0**	0.00	**0**	0.00	**2**	1.31	**0**	0.00
8	sp2 C in a ketone	Csp2 aliphatic	C[C](C)=O	**2**	1.50	**0**	0.00	**0**	0.00	**0**	0.00
9	sp2 C in an amide	Csp2 aliphatic	C[C](N)=O	**2**	1.50	**0**	0.00	**0**	0.00	**0**	0.00
10	CH in benzene or pyridine ring	Csp2 aromatic	c[c]([H])c or n[c]([H])c	**1**	1.40	**0**	0.00	**2**	0.70	**0**	0.00
11	Quaternary aromatic sp2 C atom	Csp2 aromatic	c[c](*)c or n[c](*)c	**0**	0.00	**0**	0.00	**2**	0.88	**0**	0.00
12	sp C in nitrile	Csp_C#N	C[C]#N	**4**	1.50	**0**	0.00	**0**	0.00	**0**	0.00
13	NH in secondary amide	Nsp3	[H][N](C=O)C	**1**	2.81	**0**	0.00	**2**	2.71	**0**	0.00
14	N in tertiary amide	Nsp3	C[N](C=O)C	**0**	0.00	**0**	0.00	**2**	2.71	**0**	0.00
15	NH3 in ammonia	Nsp3	[H]N([H])[H]	**3**	1.59	**0**	0.00	**1**	6.80	**1**	0.81
16	NH2 in primary amine	Nsp3	[H][N]([H])C	**2**	1.59	**0**	0.00	**1**	8.00	**1**	0.95
17	NH in secondary amine	Nsp3	[H][N](C)C	**1**	1.59	**0**	0.00	**1**	7.90	**1**	2.44
18	N in tertiary amine	Nsp3	C[N](C)C	**0**	0.00	**0**	0.00	**1**	7.50	**1**	3.59
19	sp2 N in pyridine ring	Nsp2 aromatic	[n]1ccccc1	**0**	0.00	**0**	0.00	**1**	7.30	**2**	1.00
20	sp N in nitrile	Nsp	C#[N]	**0**	0.00	**0**	0.00	**1**	5.15	**4**	1.17
21	H2O water monomer	Osp3	[H][O][H]	**2**	2.80	**0**	0.00	**2**	4.50	**0**	0.00
22	OH in a phenol	Osp3	[H][O]c1ccccc1	**1**	3.80	**0**	0.00	**1**	3.10	**2**	2.15
23	OH in alcohol	Osp3	[H][O]C	**1**	2.70	**0**	0.00	**1**	5.30	**1**	3.98
24	sp3 O in cyclic ether	Osp3	C[O;R]C	**0**	0.00	**0**	0.00	**1**	5.30	**1**	2.53
25	sp3 O in acyclic dialkyl ether	Osp3	C[O]R	**0**	0.00	**0**	0.00	**1**	5.30	**1**	2.53
26	lone pair on sp2 O in ketone	Osp2	lone pair electrons [O]=C(C)C	**0**	0.00	**0**	0.00	**1**	5.80	**1**	3.77
27	pi orbital electrons sp2 O in ketone	Osp2	Pi electrons [O]=C(C)C	**0**	0.00	**0**	0.00	**2**	0.60	**0**	0.00
28	lone pair on sp2 O in amide	Osp2	lone pair electrons [O]=C(N)	**0**	0.00	**0**	0.00	**1**	8.50	**1**	2.74
29	pi orbital electrons sp2 O in amide	Osp2	Pi electrons [O]=C(N)	**0**	0.00	**0**	0.00	**2**	0.60	**0**	0.00
30	Single F attached to sp3 C	F	[F]C([H])	**1**	0.80	**0**	0.00	**1**	3.10	**0**	0.00
31	F in perfluoroalkane (includes CF4)	F	[F]C(F)C(F)(F)	**1**	1.20	**0**	0.00	**1**	0.60	**0**	0.00
32	F attached to benzene ring	F	[F]c1ccccc1	**1**	1.52	**0**	0.00	**1**	1.52	**0**	0.00
33	S in dialkyl sulphide	S	C[S]C	**0**	0.00	**0**	0.00	**1**	3.80	**3**	1.39
34	Single Cl attached to sp3 C	Cl	[Cl]CC	**2**	1.20	**0**	0.00	**1**	2.30	**2**	0.60
35	Cl in tetrachloromethane	Cl	[Cl]C(Cl)(Cl)Cl	**2**	1.40	**0**	0.00	**3**	0.60	**0**	0.00
36	Cl attached to benzene ring	Cl	[Cl]c1ccccc1	**2**	1.50	**0**	0.00	**1**	0.85	**2**	0.85
	**Aromatic Ring Description**		Code	no of α_1 SSIP	α_1	no of α_2 SSIP	α_2	no of β_1 SSIP	β_1	no of β_2 SSIP	β_2
A1	Default e.g Benzene		A1	**0**	0.00	**0**	0.00	**1**	2.00	**0**	0.00
A2	Mono alkyl e.g Toluene		A2	**0**	0.00	**0**	0.00	**1**	2.20	**0**	0.00
A3	di-alkyl e.g. xylenes		A3	**0**	0.00	**0**	0.00	**1**	2.40	**0**	0.00
A4	s-trialkyl e.g. mesitylene		A4	**0**	0.00	**0**	0.00	**1**	2.70	**0**	0.00
A5	poly-alkyl benzene (n=4,5 or6)		A5	**0**	0.00	**0**	0.00	**1**	3.10	**0**	0.00
A6	polycyclic aromatic		A6	**0**	0.00	**0**	0.00	**1**	1.85	**0**	0.00
A7	fluorobenzene		A7	**0**	0.00	**0**	0.00	**1**	1.40	**0**	0.00
A8	chlorobenzene		A8	**0**	0.00	**0**	0.00	**1**	1.40	**0**	0.00
A9	di-halobenzene		A9	**0**	0.00	**0**	0.00	**1**	1.05	**0**	0.00
A10	phenol		A10	**0**	0.00	**0**	0.00	**1**	2.15	**0**	0.00
A11	mono-halophenol		A11	**0**	0.00	**0**	0.00	**1**	1.60	**0**	0.00
A12	di-halophenol		A12	**0**	0.00	**0**	0.00	**1**	1.25	**0**	0.00
A13	pyridine		A13	**0**	0.00	**0**	0.00	**1**	1.60	**0**	0.00
A14	monoalkyl phenol		A14	**0**	0.00	**0**	0.00	**1**	2.35	**0**	0.00
A15	di-alkyl phenol		A15	**0**	0.00	**0**	0.00	**1**	2.55	**0**	0.00
A16	mono-methyl pryridines		A16	**0**	0.00	**0**	0.00	**1**	2.00	**0**	0.00
A17	di-methyl pyridines		A17	**0**	0.00	**0**	0.00	**1**	2.40	**0**	0.00
A18	quinoline		A18	**0**	0.00	**0**	0.00	**1**	1.60	**0**	0.00
A19	isoquinoline		A19	**0**	0.00	**0**	0.00	**1**	1.60	**0**	0.00

Section 4:
Free Energy of Formation for H-bonded 1:1 complexes

a) Benzene

Solvent		α_S	C_α	β_S	C_β
Benzene		1.40	2.50	2.00	1.09

Figure S4a

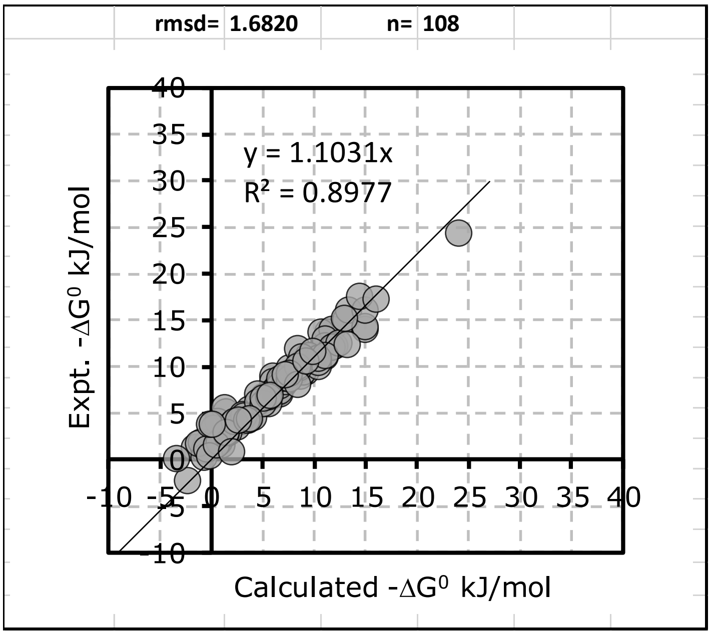

Free energy calculation for 1:1 Association

$$-\Delta G^0 = \alpha\beta - \alpha\beta_S - \alpha_S\beta - C_\alpha - C_\beta$$

Table S4a	Donor		Acceptor		Calc.	Expt.
Reference	DH	α	A	β	-ΔG⁰	-ΔG⁰
[1]	phenol	3.80	pyridine	7.20	6.09	8.90
[1]	propan-2-ol	2.70	pyridine	7.20	0.37	1.72
[1]	Ethanol	2.70	Propan-2-one	5.70	-1.58	1.16
[1]	3-fluorophenol	4.10	pyridine	7.20	7.65	9.84
[1]	3-fluorophenol	4.10	dimethyl sulphoxide	8.60	11.43	13.72
[1]	3-fluorophenol	4.10	Ethyl ethanoate	5.40	2.79	3.99
[1]	butan-1-ol	2.70	pyridine	7.20	0.37	1.72
[1]	3-fluorophenol	4.10	triethylamine	7.50	8.46	11.86
[1]	2,2,2-trifluoroethanol	3.70	benzophenone	5.40	1.43	5.03
[2]	2,2,2-Trifluoro-1,1-bis(trifluoromethyl)ethanol	4.90	Tributylphosphine oxide	10.70	24.06	24.29
[3]	Ethanol	2.70	Ethanol	5.20	-2.23	-2.26
[4]	phenol	3.80	tetrahydrofuran	5.90	2.97	3.99
[5]	4-nitrophenol	4.70	Benzylamine	7.20	10.77	13.64
[5]	4-nitrophenol	4.70	pyridine	7.20	10.77	11.80
[5]	4-nitrophenol	4.70	tributylamine	6.80	9.45	10.59
[5]	4-nitrophenol	4.70	tripropylamine	6.60	8.79	10.21
[5]	4-nitrophenol	4.70	triethylamine	7.50	11.76	13.89
[5]	4-nitrophenol	4.70	di-n-butylamine	7.90	13.08	15.02
[5]	4-nitrophenol	4.70	n-butylamine	8.00	13.41	16.07

[6]	2,2,3,3-Tetrafluoropropan-1-ol	**3.50**	di-n-butylamine	**7.90**	6.00	8.34
[6]	2,2,3,3-Tetrafluoropropan-1-ol	**3.50**	Trioctylamine	**7.00**	4.11	4.44
[6]	2,2,3,3-Tetrafluoropropan-1-ol	**3.50**	tributylamine	**6.80**	3.69	5.44
[6]	phenol	**3.80**	Trioctylamine	**7.00**	5.61	7.02
[6]	phenol	**3.80**	tributylamine	**6.80**	5.13	6.71
[6]	Propan-1-ol	**2.60**	di-n-butylamine	**7.90**	0.69	3.99
[6]	Propan-1-ol	**2.60**	Trioctylamine	**7.00**	-0.39	1.72
[6]	Propan-1-ol	**2.60**	tributylamine	**6.80**	-0.63	1.00
**[4]**	2,6-Dimethylphenol	**3.30**	tetrahydrofuran	**5.90**	1.02	1.46
**[4]**	phenol	**3.80**	tetrahydrofuran	**5.90**	2.97	3.99
[6]	4-tert-Butylphenol	**3.60**	acetophenone	**5.50**	1.31	5.55
[7]	phenol	**3.80**	acetophenone	**5.50**	2.01	3.67
[7]	4-bromophenol	**4.10**	acetophenone	**5.50**	3.06	4.75
[7]	4-fluorophenol	**3.90**	N,N-dimethylacetamide	**8.50**	9.86	11.10
[7]	4-bromophenol	**4.10**	N,N-dimethylacetamide	**8.50**	11.16	12.11
[7]	4-nitrophenol	**4.70**	N,N-dimethylacetamide	**8.50**	15.06	14.01
[7]	4-bromophenol	**4.10**	Propan-2-one	**5.70**	3.60	4.13
[7]	phenol	**3.80**	Propan-2-one	**5.70**	2.49	3.51
[7]	phenol	**3.80**	1,1,3,3-Tetramethylurea	**8.50**	9.21	9.32
[7]	4-chlorophenol	**4.10**	1,1,3,3-Tetramethylurea	**8.50**	11.16	11.00
[7]	4-bromophenol	**4.10**	1,1,3,3-Tetramethylurea	**8.50**	11.16	12.91
[7]	4-nitrophenol	**4.70**	1,1,3,3-Tetramethylurea	**8.50**	15.06	14.20
[7]	4-tert-Butylphenol	**3.60**	Propan-2-one	**5.70**	1.75	2.80
[8]	2-Methoxyphenol	**2.40**	triethylamine	**7.50**	-0.89	1.79
[8]	2-Methoxyphenol	**2.40**	N,N-Dimethylformamide	**7.70**	-0.69	0.14
[9]	2-Methoxyphenol	**2.40**	dimethyl sulphoxide	**8.60**	0.21	3.27
[9]	phenol	**3.80**	dimethyl sulphoxide	**8.60**	9.45	11.07
[10]	2-Methoxyphenol	**2.40**	pyridine	**7.20**	-1.19	1.61
[11]	butan-1-ol	**2.70**	tripropylamine	**6.60**	-0.41	1.10
[11]	butan-1-ol	**2.70**	triethylamine	**7.50**	0.76	1.80
[11]	butan-1-ol	**2.70**	tributylamine	**6.80**	-0.15	0.40
[6]	phenol	**3.80**	diethyl ether	**5.30**	1.53	2.65
[6]	phenol	**3.80**	Hexamethylphosphoramide	**10.90**	14.97	16.00
[6]	3-methylphenol	**3.70**	pyridine	**7.20**	5.57	6.90
[6]	4-methylphenol	**3.70**	pyridine	**7.20**	5.57	6.50
[6]	4-Methoxyphenol	**3.70**	2-methylpyridine	**7.60**	6.49	7.18
[6]	4-methylphenol	**3.70**	2-methylpyridine	**7.60**	6.49	7.70
[6]	3-methylphenol	**3.70**	2-methylpyridine	**7.60**	6.49	7.07
[6]	phenol	**3.80**	2-methylpyridine	**7.60**	7.05	7.98
[6]	4-chlorophenol	**4.10**	2-methylpyridine	**7.60**	8.73	8.86
[6]	4-nitrophenol	**4.70**	2-methylpyridine	**7.60**	12.09	12.12
[6]	4-Methoxyphenol	**3.70**	3-methylpyridine	**7.50**	6.26	7.01
[6]	4-methylphenol	**3.70**	3-methylpyridine	**7.50**	6.26	7.18
[6]	3-methylphenol	**3.70**	3-methylpyridine	**7.50**	6.26	7.51
[6]	phenol	**3.80**	3-methylpyridine	**7.50**	6.81	8.44
[6]	4-chlorophenol	**4.10**	3-methylpyridine	**7.50**	8.46	9.46
[6]	4-nitrophenol	**4.70**	3-methylpyridine	**7.50**	11.76	12.05

[6]	4-Methoxyphenol	**3.70**	4-methylpyridine	**7.70**	6.72	7.04
[6]	4-methylphenol	**3.70**	4-methylpyridine	**7.70**	6.72	7.29
[6]	3-methylphenol	**3.70**	4-methylpyridine	**7.70**	6.72	7.84
[6]	phenol	**3.80**	4-methylpyridine	**7.70**	7.29	8.32
[6]	4-chlorophenol	**4.10**	4-methylpyridine	**7.70**	9.00	9.29
[6]	4-Methoxyphenol	**3.70**	pyridine	**7.20**	5.57	5.94
[6]	4-nitrophenol	**4.70**	4-methylpyridine	**7.70**	12.42	12.49
[6]	4-Methoxyphenol	**3.70**	4-N,N-dimethylaminopyridine	**9.30**	10.40	10.01
[6]	4-methylphenol	**3.70**	4-N,N-dimethylaminopyridine	**9.30**	10.40	10.44
[6]	3-methylphenol	**3.70**	4-N,N-dimethylaminopyridine	**9.30**	10.40	10.78
[6]	phenol	**3.80**	4-N,N-dimethylaminopyridine	**9.30**	11.13	11.08
[6]	4-chlorophenol	**4.10**	4-N,N-dimethylaminopyridine	**9.30**	13.32	12.32
[11]	3-methylphenol	**3.70**	dibutyl ether	**5.00**	0.51	1.59
[11]	3-methylphenol	**3.70**	1,4-dioxane	**4.70**	-0.18	3.73
[11]	3-methylphenol	**3.70**	anisole	**3.30**	-3.40	0.00
[11]	3-methylphenol	**3.70**	N,N-Dimethylformamide	**7.70**	6.72	8.66
[11]	3-methylphenol	**3.70**	N,N-dimethylacetamide	**8.50**	8.56	9.84
[11]	3-methylphenol	**3.70**	cyclohexanone	**6.20**	3.27	4.60
[11]	3-methylphenol	**3.70**	dimethyl sulphoxide	**8.60**	8.79	11.04
[11]	3-methylphenol	**3.70**	tetrahydropyran	**5.80**	2.35	4.09
[11]	3-methylphenol	**3.70**	Ethyl ethanoate	**5.40**	1.43	2.88
[12]	phenol	**3.80**	1,4-dioxane	**4.70**	0.09	3.78
[13]	2,3-Dimethylphenol	**3.50**	pyridine	**7.20**	4.53	7.02
[13]	2,4-Dimethylphenol	**3.50**	pyridine	**7.20**	4.53	6.11
[13]	2,5-Dimethylphenol	**3.60**	pyridine	**7.20**	5.05	5.94
[13]	3,5-Dimethylphenol	**3.70**	pyridine	**7.20**	5.57	6.71
[6]	4-bromophenol	**4.10**	pyridine	**7.20**	7.65	8.66
[6]	4-bromophenol	**4.10**	triethylamine	**7.50**	8.46	9.91
[6]	4-chlorophenol	**4.10**	pyridine	**7.20**	7.65	9.17
[6]	4-chlorophenol	**4.10**	triethylamine	**7.50**	8.46	8.05
[6]	3,4-Dimethylphenol	**3.60**	pyridine	**7.20**	5.05	6.54
[6]	3,4-Dimethylphenol	**3.60**	triethylamine	**7.50**	5.71	6.87
[6]	2,6-Dimethylphenol	**3.30**	pyridine	**7.20**	3.49	4.13
[12]	phenol	**3.80**	N,N-Dimethylformamide	**7.70**	7.29	9.02
[12]	phenol	**3.80**	N,N-dimethylacetamide	**8.50**	9.21	10.57
[12]	phenol	**3.80**	cyclohexanone	**6.20**	3.69	4.40
[12]	phenol	**3.80**	tetrahydropyran	**5.80**	2.73	4.13
[14]	tert-Butyl alcohol	**2.70**	N,N-diethylacetamide	**8.50**	2.06	0.85
[15]	phenol	**3.80**	Tributylphosphine oxide	**10.70**	14.49	17.46
[15]	phenol	**3.80**	triethyl phosphate	**8.80**	9.93	11.58
[15]	4-nitrophenol	**4.70**	triethyl phosphate	**8.80**	16.05	17.17
[15]	phenol	**3.80**	triphenylphosphine oxide	**10.10**	13.05	15.12

b) Toluene

Solvent		α_S	C_α	β_S	C_β
Toluene		1.40	2.50	2.00	1.09

Figure S4b

rmsd= 1.6836 n= 46

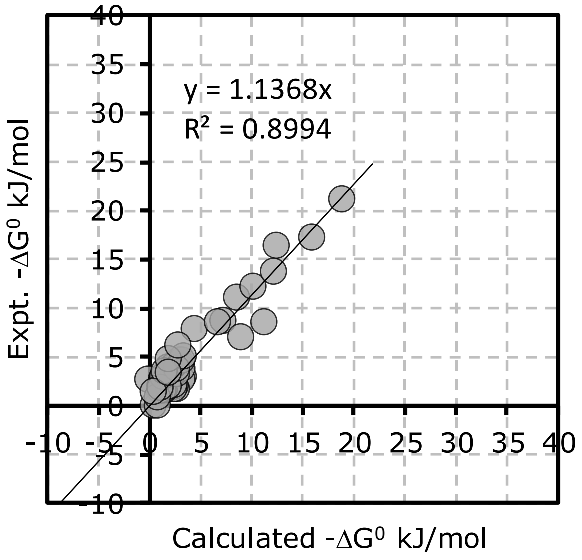

Free energy calculation for 1:1 Association

$$-\Delta G^0 = \alpha\beta - \alpha\beta_S - \alpha_S\beta - C_\alpha - C_\beta$$

Table S4b	Donor		Acceptor		Calc.	Expt.
Reference	DH	Alpha	A	Beta	$-\Delta G^0$	$-\Delta G^0$
**[16]**	4-methylphenol	**3.70**	Methyl ethanoate	**4.70**	-0.18	2.72
[16]	4-methylphenol	**3.70**	N,N-diethylacetamide	**8.50**	8.56	11.04
[17]	4-phenylazophenol	**4.30**	Tributylphosphine oxide	**10.70**	18.84	21.11
[16]	4-methylphenol	**3.70**	Ethyl ethanoate	**5.40**	1.43	2.72
[16]	4-methylphenol	**3.70**	Ethyl 4-methylbenzoate	**5.40**	1.43	2.72
[16]	4-methylphenol	**3.70**	N,N-diethyl-4-methylbenzamide	**7.90**	7.18	8.66
[18]	3,5,5-Trimethyl-hexanoic acid phenylamide	**2.90**	Diethyl ethylphosphonate	**9.20**	4.41	7.87
[19]	4-methylphenol	**3.70**	Diethyl ethylphosphonate	**9.20**	10.17	12.24
[20]	4-methylphenol	**3.70**	diethyl ether	**5.30**	1.20	2.72
[21]	4-methylphenol	**3.70**	n-butyl-di-tert-butylphosphine oxide	**10.20**	12.47	16.44
[22]	4-phenylazophenol	**4.30**	N,N-di-n-hexylacetamide	**8.40**	12.17	13.75
[23]	4-methylphenol	**3.70**	4-methylpyridine	**7.70**	6.72	8.51
[24]	2,6-Dimethylphenol	**3.30**	tetrahydrofuran	**5.90**	1.02	2.17
[25]	pyrrole	**3.00**	2,4,6-trimethylpyridine	**8.10**	3.37	2.95
[25]	pyrrole	**3.00**	3,5-Dimethylpyridine	**8.00**	3.21	2.65
[25]	pyrrole	**3.00**	4-methylpyridine	**7.70**	2.73	1.68
[25]	pyrrole	**3.00**	pyridine	**7.20**	1.93	1.68
[25]	pyrrole	**3.00**	2-methylpyridine	**7.60**	2.57	1.99
[25]	pyrrole	**3.00**	3-methylpyridine	**7.50**	2.41	1.67
[25]	pyrrole	**3.00**	triethylamine	**7.50**	2.41	2.17
[26]	propan-2-ol	**2.70**	2,4,6-trimethylpyridine	**8.10**	1.54	1.50
[26]	propan-2-ol	**2.70**	pyridine	**7.20**	0.37	0.02
[26]	benzyl alcohol	**3.00**	pyridine	**7.20**	1.93	3.95

[26]	propan-2-ol	**2.70**	triethylamine	**7.50**	0.76	-0.02
[26]	benzyl alcohol	**3.00**	triethylamine	**7.50**	2.41	3.24
[26]	propan-2-ol	**2.70**	3,5-Dimethylpyridine	**8.00**	1.41	1.49
[26]	benzyl alcohol	**3.00**	3,5-Dimethylpyridine	**8.00**	3.21	3.84
[26]	benzyl alcohol	**3.00**	4-methylpyridine	**7.70**	2.73	3.18
[26]	propan-2-ol	**2.70**	4-methylpyridine	**7.70**	1.02	1.49
G12	pyrrole	**3.00**	2,4-Dimethylpyridine	**7.20**	1.93	2.00
G6	phenol	**3.80**	tetrahydrofuran	**5.90**	2.97	4.68
G77	3-Chlorophenol	**4.20**	triethylamine	**7.50**	9.01	6.98
G77	3-nitrophenol	**4.60**	triethylamine	**7.50**	11.21	8.49
G100	phenol	**3.80**	Tri-n-octylphosphine oxide	**11.30**	15.93	17.27
[26]	propan-2-ol-D	**2.70**	triethylamine	**7.50**	0.76	0.78
[26]	benzyl alcohol-D	**3.00**	triethylamine	**7.50**	2.41	4.07
[26]	propan-2-ol-D	**2.70**	3,5-Dimethylpyridine	**8.00**	1.41	2.95
[26]	benzyl alcohol-D	**3.00**	3,5-Dimethylpyridine	**8.00**	3.21	4.58
[26]	propan-2-ol-D	**2.70**	4-methylpyridine	**7.70**	1.02	1.85
[26]	benzyl alcohol-D	**3.00**	4-methylpyridine	**7.70**	2.73	3.80
[26]	benzyl alcohol-D	**3.00**	2,4,6-trimethylpyridine	**8.10**	3.37	5.02
[26]	propan-2-ol-D	**2.70**	2,4,6-trimethylpyridine	**8.10**	1.54	3.52
[27]	Methanol-D	**2.90**	triethylamine	**7.50**	1.86	4.74
[27]	Methanol-D	**2.90**	2,4,6-trimethylpyridine	**8.10**	2.76	6.18
[20]	propan-2-ol-D	**2.70**	pyridine	**7.20**	0.37	1.46
[20]	benzyl alcohol-D	**3.00**	pyridine	**7.20**	1.93	3.33

c) Hexane

Solvent		α_S	C_α	β_S	C_β
**Hexane**		1.20	2.62	0.60	2.62

Figure S4c

rmsd= 0.6833 **n= 6**

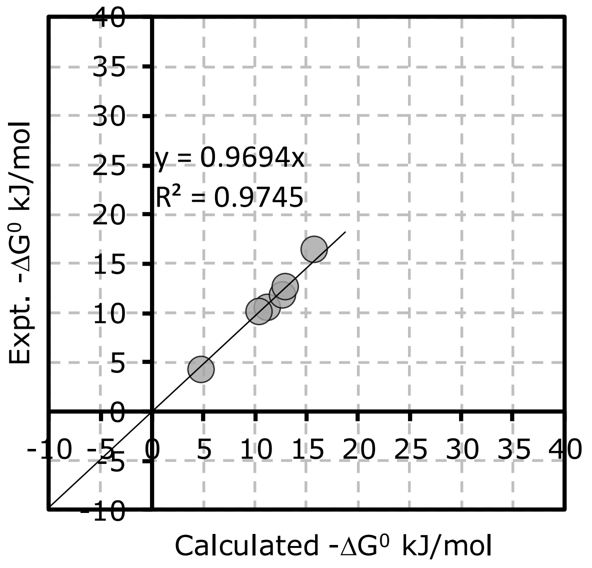

Free energy calculation for 1:1 Association

$$-\Delta G^0 = \alpha\beta - \alpha\beta_S - \alpha_S\beta - C_\alpha - C_\beta$$

**Table S4c**	**Donor**		**Acceptor**		**Calc.**	**Expt.**
**Reference**	**DH**	**Alpha**	**A**	**Beta**	**$-\Delta G^0$**	**$-\Delta G^0$**
[28]	2,2,2-trifluoroethanol	**3.70**	triethylamine	**7.50**	11.25	10.46
[1]	Hexafluoropropan-2-ol	**4.50**	pyridine	**7.20**	15.78	16.34
[28]	2,2,2-trifluoroethanol	**3.70**	2,4,6-trimethylpyridine	**8.10**	12.75	11.72
[1]	2,2,2-trifluoroethanol	**3.70**	pyridine	**7.20**	10.50	10.10
[28]	1-naphthol	**3.80**	di-n-butylamine	**7.90**	12.98	12.61
[28]	Hexafluoropropan-2-ol	**4.50**	tetrahydrothiophene	**3.90**	4.89	4.18

d) Cyclohexane

Solvent	α_S	C_α	β_S	C_β
**Cyclohexane**	1.20	2.61	0.60	2.61

Figure S4d

rmsd= 1.2330 **n= 109**

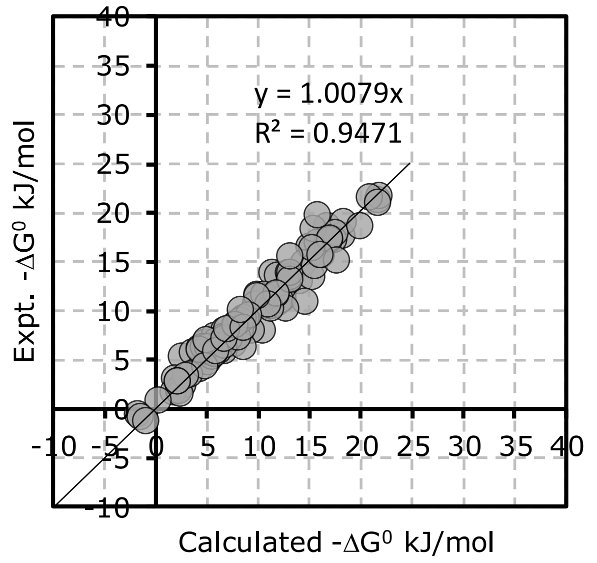

Free energy calculation for 1:1 Association

$$-\Delta G^0 = \alpha\beta - \alpha\beta_S - \alpha_S\beta - C_\alpha - C_\beta$$

Table S4d	Donor		Acceptor		Calc.	Expt.
**Reference**	**DH**	**Alpha**	**A**	**Beta**	**-ΔG⁰**	**-ΔG⁰**
[1]	phenol	**3.80**	tetrahydrothiophene	**3.90**	2.58	5.31
[1]	4-chlorophenol	**4.10**	tetrahydrothiophene	**3.90**	3.57	5.71
[1]	Hexafluoropropan-2-ol	**4.50**	tetrahydrothiophene	**3.90**	4.89	6.68
[1]	4-nitrophenol	**4.70**	diethyl ether	**5.30**	10.45	7.99
[1]	4-tert-Butylphenol	**3.60**	tetrahydrothiophene	**3.90**	1.92	3.02
[1]	4-chlorophenol	**4.10**	tripropylamine	**6.60**	11.40	13.81
[29]	4-fluorophenol	**3.90**	triethylamine	**7.50**	12.63	12.22
[28]	3-trifluoromethylphenol	**4.30**	tetrahydrothiophene	**3.90**	4.23	4.10
[1]	2,2,2-trifluoroethanol	**3.70**	pyridine N-oxide	**9.00**	15.00	16.54
[1]	4-chlorophenol	**4.10**	tributylamine	**6.80**	11.98	13.64
[1]	4-fluorophenol	**3.90**	pyridine N-oxide	**9.00**	16.68	18.66
[1]	4-chlorophenol	**4.10**	tetrahydrofuran	**5.90**	9.37	8.04
[1]	3-methylphenol	**3.70**	1,4-dioxane	**4.70**	4.25	6.16
[1]	4-chlorophenol	**4.10**	triethylamine	**7.50**	14.01	13.06
[1]	4-tert-Butylphenol	**3.60**	N,N-dimethylacetamide	**8.50**	12.96	13.81
[1]	4-chlorophenol	**4.10**	diethyl ether	**5.30**	7.63	6.56
[1]	phenol	**3.80**	triethylamine	**7.50**	11.94	11.01
[1]	3-nitrophenol	**4.60**	diethyl ether	**5.30**	9.98	11.70
[1]	4-fluorophenol	**3.90**	1,4-dioxane	**4.70**	5.07	4.74
[1]	Propan-1-ol	**2.60**	pyridine N-oxide	**9.00**	5.76	7.36
[1]	4-nitrophenol	**4.70**	triethylamine	**7.50**	18.15	17.69
[1]	1-naphthol	**3.80**	4-methylpyridine	**7.70**	12.46	11.18
[1]	phenol	**3.80**	pyridine N-oxide	**9.00**	15.84	17.00

[1]	4-tert-Butylphenol	**3.60**	tetrahydrofuran	**5.90**	6.72	7.99
[1]	Hexafluoropropan-2-ol	**4.50**	pyridine N-oxide	**9.00**	21.72	21.28
[1]	1-naphthol	**3.80**	pyridine	**7.20**	11.16	10.44
[1]	4-fluorophenol	**3.90**	Hexamethylphosphoramide	**10.90**	21.81	21.68
[1]	1-naphthol	**3.80**	tetrahydrofuran	**5.90**	7.78	7.42
[1]	tert-Butyl alcohol	**2.70**	pyridine N-oxide	**9.00**	6.60	7.19
[1]	4-tert-Butylphenol	**3.60**	pyridine	**7.20**	9.84	10.61
[1]	2-naphthol	**3.90**	diethyl ether	**5.30**	6.69	5.93
[1]	propan-2-ol	**2.70**	pyridine N-oxide	**9.00**	6.60	7.25
[1]	3-trifluoromethylphenol	**4.30**	Ethyl ethanoate	**5.40**	8.88	9.30
[1]	3-trifluoromethylphenol	**4.30**	pyridine	**7.20**	14.46	14.83
[1]	1-naphthol	**3.80**	diethyl ether	**5.30**	6.22	5.93
[1]	2-naphthol	**3.90**	tetrahydrofuran	**5.90**	8.31	7.64
[1]	3-trifluoromethylphenol	**4.30**	cyclohexanone	**6.20**	11.36	10.55
[1]	3-fluorophenol	**4.10**	diethyl sulphide	**3.60**	2.70	2.34
[1]	4-chlorophenol	**4.10**	pyridine N-oxide	**9.00**	18.36	19.11
[1]	4-tert-Butylphenol	**3.60**	triethylamine	**7.50**	10.56	11.58
[1]	1-naphthol	**3.80**	3-methylpyridine	**7.50**	11.94	10.84
[1]	3,5-dichlorophenol	**4.50**	triethylamine	**7.50**	16.77	16.89
[1]	3-nitrophenol	**4.60**	triethylamine	**7.50**	17.46	17.23
[1]	Ethanol	**2.70**	pyridine N-oxide	**9.00**	6.60	7.30
[28]	4-chlorophenol	**4.10**	pyridine	**7.20**	13.14	13.39
[28]	3-trifluoromethylphenol	**4.30**	diethyl sulphide	**3.60**	3.30	3.56
[1]	4-chlorophenol	**4.10**	1,4-dioxane	**4.70**	5.89	6.85
[1]	1-naphthol	**3.80**	2-methylpyridine	**7.60**	12.20	11.07
[1]	phenol	**3.80**	dibutyl ether	**5.00**	5.44	5.31
[1]	phenol	**3.80**	diethyl sulphide	**3.60**	1.80	1.71
[1]	4-fluorophenol	**3.90**	benzonitrile	**4.80**	5.34	5.71
[1]	3-methylphenol	**3.70**	diethyl ether	**5.30**	5.75	5.71
[1]	3-methylphenol	**3.70**	tetrahydrofuran	**5.90**	7.25	7.30
[1]	Methanol	**2.90**	pyridine N-oxide	**9.00**	8.28	7.99
[1]	3-fluorophenol	**4.10**	dibutyl ether	**5.00**	6.76	7.02
[1]	4-fluorophenol	**3.90**	4-N,N-dimethylaminopyridine	**9.30**	17.49	17.97
[1]	4-fluorophenol	**3.90**	Diethyl chloromethylphosphonate	**8.50**	15.33	13.52
[1]	phenol	**3.80**	Diethyl chloromethylphosphonate	**8.50**	14.54	10.86
[1]	2-tert-Butylphenol	**3.40**	Diethyl isopropylphosphonate	**9.10**	12.70	10.27
[1]	4-fluorophenol	**3.90**	pyridine	**7.20**	11.82	11.58
[1]	4-fluorophenol	**3.90**	3-bromopyridine	**6.00**	8.58	6.33
[1]	3-fluorophenol	**4.10**	pyridine	**7.20**	13.14	13.81
[1]	3-fluorophenol	**4.10**	Ethyl ethanoate	**5.40**	7.92	8.73
[1]	1-naphthol	**3.80**	1,4-dioxane	**4.70**	4.66	6.33
[1]	2-naphthol	**3.90**	1,4-dioxane	**4.70**	5.07	6.33
[30]	4-fluorophenol	**3.90**	dimethyl sulphoxide	**8.60**	15.60	14.61
[30]	4-fluorophenol	**3.90**	N,N-Dimethylformamide	**7.70**	13.17	13.12
[28]	4-chlorophenol	**4.10**	n-butylamine	**8.00**	15.46	18.41
[28]	4-methylphenol	**3.70**	triethylamine	**7.50**	11.25	10.17

[28]	4-methylphenol	**3.70**	diethyl ether	**5.30**	5.75	5.67
[28]	4-methylphenol	**3.70**	tetrahydrofuran	**5.90**	7.25	7.21
[28]	4-methylphenol	**3.70**	1,4-dioxane	**4.70**	4.25	5.98
[28]	4-methylphenol	**3.70**	Propan-2-one	**5.70**	6.75	6.46
[28]	4-methylphenol	**3.70**	cyclohexanone	**6.20**	8.00	7.28
[28]	4-methylphenol	**3.70**	tetrahydrothiophene	**3.90**	2.25	1.84
[3]	butan-1-ol	**2.70**	cyclohexanone	**6.20**	2.40	1.50
[31]	Butan-2-ol	**2.50**	pyridine N-oxide	**9.00**	4.92	7.02
[29]	4-fluorophenol	**3.90**	2,2,2-Trifluoroethylamine	**4.60**	4.80	4.39
[29]	4-fluorophenol	**3.90**	Benzylamine	**7.20**	11.82	11.76
[29]	4-fluorophenol	**3.90**	Cyclopropylamine	**6.90**	11.01	10.61
[29]	4-fluorophenol	**3.90**	Pyrrolidine	**8.80**	16.14	15.74
[29]	4-fluorophenol	**3.90**	quinuclidine	**9.10**	16.95	17.19
[29]	4-fluorophenol	**3.90**	3,5-Dichloropyridine	**5.00**	5.88	5.82
[29]	4-fluorophenol	**3.90**	3-Chloropyridine	**6.00**	8.58	9.27
[29]	4-fluorophenol	**3.90**	1-Methyl-1H-imidazole	**9.10**	16.95	17.33
[29]	4-fluorophenol	**3.90**	Dimethylcyanamide	**6.50**	9.93	11.47
[29]	4-fluorophenol	**3.90**	tetrahydrofuran	**5.90**	8.31	8.94
[29]	4-fluorophenol	**3.90**	diethyl ether	**5.30**	6.69	7.12
[29]	4-fluorophenol	**3.90**	2,2,5,5-tetramethyltetrahydrofuran	**6.20**	9.12	9.53
[29]	4-fluorophenol	**3.90**	Propan-2-one	**5.70**	7.77	8.55
[29]	4-fluorophenol	**3.90**	Ethyl ethanoate	**5.40**	6.96	8.06
[29]	4-fluorophenol	**3.90**	N,N-dimethylacetamide	**8.50**	15.33	16.39
[29]	4-fluorophenol	**3.90**	dibutyl sulphoxide	**8.70**	15.87	19.82
[29]	4-fluorophenol	**3.90**	N,N-Dimethylthioacetamide	**6.00**	8.58	8.33
[29]	4-fluorophenol	**3.90**	tetrahydrothiophene	**3.90**	2.91	3.31
[29]	4-fluorophenol	**3.90**	diethyl sulphide	**3.60**	2.10	2.83
[29]	4-fluorophenol	**3.90**	dibutyl sulphide	**3.60**	2.10	2.80
[29]	4-fluorophenol	**3.90**	1-Fluoropentane	**2.90**	0.21	0.82
[29]	4-fluorophenol	**3.90**	1-Chloropentane	**2.20**	-1.68	-0.59
[29]	4-fluorophenol	**3.90**	1-Bromopentane	**2.30**	-1.41	-0.77
[29]	4-fluorophenol	**3.90**	1-Iodopentane	**2.50**	-0.87	-1.27
[28]	4-nitrophenol	**4.70**	n-butylamine	**8.00**	19.90	18.58
[28]	4-nitrophenol	**4.70**	1,4-dioxane	**4.70**	8.35	10.08
[28]	3-Chlorophenol	**4.20**	N,N-dimethylacetamide	**8.50**	17.70	15.06
[1]	4-chlorophenol	**4.10**	morpholine	**7.20**	13.14	15.52
[1]	3-trifluoromethyl-4-nitrophenol	**5.10**	triethylamine	**7.50**	20.91	21.62
[1]	Pentafluorophenol	**4.50**	pyridine N-oxide	**9.00**	21.72	21.05
[1]	3,4-dichlorophenol	**4.40**	triethylamine	**7.50**	16.08	15.69
[1]	4-cyanophenol	**4.60**	triethylamine	**7.50**	17.46	16.94

e) Carbon Tetrachloride

Solvent		α_S	C_α	β_S	C_β
**CCl4**		1.40	2.58	0.60	2.58

Figure S4e

rmsd= **0.8198** **n=** **475**

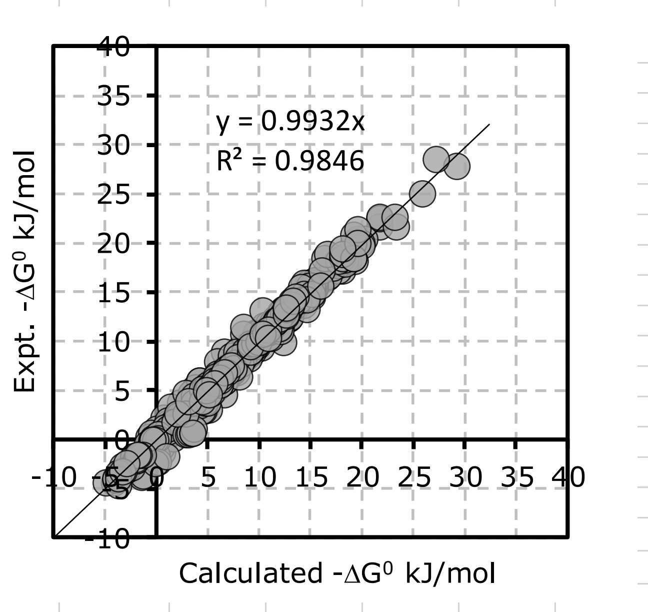

Free energy calculation for 1:1 Association

$$-\Delta G^0 = \alpha\beta - \alpha\beta_S - \alpha_S\beta - C_\alpha - C_\beta$$

in CCl4 solvent $C_\alpha = C_\beta = 2.58$ therefore:

$$-\Delta G^0 = \alpha\beta - \alpha\beta_S - \alpha_S\beta - 5.16$$

$$= \alpha\beta - \alpha\beta_S - \alpha_S\beta + \alpha_S\beta_S - 6$$

$$= (\alpha - \alpha_S)(\beta - \beta_S) - 6$$

Table S4e	Donor		Acceptor		Calc.	Expt.
Reference	DH	Alpha	A	Beta	$-\Delta G^0$	$-\Delta G^0$
[1]	3-Chlorophenol	**4.20**	aniline	**4.50**	4.92	4.22
[1]	3-Chlorophenol	**4.20**	pyridine	**7.20**	12.48	9.81
[28]	phenol	**3.80**	tetrahydrothiophene	**3.90**	1.92	0.83
[28]	1-naphthol	**3.80**	toluene	**2.20**	-2.16	-2.44
[28]	3-trifluoromethylphenol	**4.30**	tetrahydrothiophene	**3.90**	3.57	2.63
[29]	4-chlorophenol	**4.10**	tetrahydrofuran	**5.90**	8.31	8.22
[28]	4-chlorophenol	**4.10**	triethylamine	**7.50**	12.63	12.06
[28]	4-chlorophenol	**4.10**	diethyl ether	**5.30**	6.69	6.12
[28]	phenol	**3.80**	triethylamine	**7.50**	10.56	10.49
[32]	4-fluorophenol	**3.90**	1,4-dioxane	**4.70**	4.25	5.88
[29]	4-nitrophenol	**4.70**	triethylamine	**7.50**	16.77	16.44
[28]	phenol	**3.80**	pyridine N-oxide	**9.00**	14.16	14.41
[28]	1-naphthol	**3.80**	pyridine	**7.20**	9.84	10.74
[33]	Ethanol	**2.70**	pyridine N-oxide	**9.00**	4.92	4.48
[29]	4-chlorophenol	**4.10**	pyridine	**7.20**	11.82	11.98
[28]	phenol	**3.80**	diethyl sulphide	**3.60**	1.20	0.24
[34]	4-fluorophenol	**3.90**	benzonitrile	**4.80**	4.50	4.56
[28]	3-fluorophenol	**4.10**	dibutyl ether	**5.00**	5.88	5.96
[28]	4-fluorophenol	**3.90**	4-N,N-dimethylaminopyridine	**9.30**	15.75	16.05
[28]	1-naphthol	**3.80**	triethylamine	**7.50**	10.56	11.43
[28]	2,2,2-trifluoroethanol	**3.70**	triethylamine	**7.50**	9.87	10.56

[29]	phenol	**3.80**	pyridine	**7.20**	9.84	9.64
[28]	Ethanol	**2.70**	N,N-dimethylacetamide	**8.50**	4.27	3.92
[29]	2,2,2-trifluoroethanol	**3.70**	N,N-dimethylacetamide	**8.50**	12.17	11.40
[28]	2,2,2-trifluoroethanol	**3.70**	dimethyl sulphoxide	**8.60**	12.40	12.13
[29]	2,2,2-trifluoroethanol	**3.70**	Hexamethylphosphoramide	**10.90**	17.69	17.63
[28]	2,2,2-trifluoroethanol	**3.70**	diethyl ether	**5.30**	4.81	4.18
[28]	2,2,2-trifluoroethanol	**3.70**	Propan-2-one	**5.70**	5.73	4.93
[28]	2,2,2-trifluoroethanol	**3.70**	N,N-Dimethylformamide	**7.70**	10.33	9.20
[28]	phenol	**3.80**	Propanaldehyde	**4.60**	3.60	3.66
[28]	phenol	**3.80**	diethyl ether	**5.30**	5.28	5.40
[28]	phenol	**3.80**	N,N-dimethylacetamide	**8.50**	12.96	12.14
[29]	phenol	**3.80**	Propan-2-one	**5.70**	6.24	5.89
[28]	phenol	**3.80**	Hexamethylphosphoramide	**10.90**	18.72	18.36
[28]	phenol	**3.80**	benzene	**2.00**	-2.64	-2.77
[28]	phenol	**3.80**	dimethyl sulphoxide	**8.60**	13.20	13.15
[35]	Methanol	**2.90**	Propan-2-one	**5.70**	1.65	1.03
[28]	Methanol	**2.90**	Triethylphosphine oxide	**10.10**	8.25	9.20
[33]	Ethanol	**2.70**	Ethanol	**5.20**	-0.02	-1.11
[33]	Octan-1-ol	**2.70**	Octan-1-ol	**5.30**	0.11	-0.65
[33]	tert-Butyl alcohol	**2.70**	tert-Butyl alcohol	**5.70**	0.63	-1.34
[28]	propan-2-ol	**2.70**	N,N-dimethylacetamide	**8.50**	4.27	2.60
[31]	4-fluorophenol	**3.90**	3-bromopyridine	**6.00**	7.50	7.72
[28]	4-fluorophenol	**3.90**	triethyl phosphate	**8.80**	14.50	13.98
[29]	Methanol	**2.90**	diethyl ether	**5.30**	1.05	0.41
[35]	Methanol	**2.90**	acetonitrile	**5.10**	0.75	0.78
[28]	Methanol	**2.90**	Trimethylphosphine oxide	**10.70**	9.15	9.05
[28]	Methanol	**2.90**	triethyl phosphate	**8.80**	6.30	6.69
[29]	Methanol	**2.90**	dimethyl sulphoxide	**8.60**	6.00	5.24
[28]	Methanol	**2.90**	N,N-Dimethylformamide	**7.70**	4.65	4.22
[29]	4-chlorophenol	**4.10**	N,N-dimethylacetamide	**8.50**	15.33	14.39
[28]	4-fluorophenol	**3.90**	Benzaldehyde	**4.80**	4.50	4.44
[28]	4-fluorophenol	**3.90**	diethyl sulphide	**3.60**	1.50	0.65
[28]	propan-2-ol	**2.70**	pyridine	**7.20**	2.58	2.09
[28]	propan-2-ol	**2.70**	diethyl ether	**5.30**	0.11	-0.55
[28]	tert-Butyl alcohol	**2.70**	N,N-Dimethylformamide	**7.70**	3.23	2.64
[28]	tert-Butyl alcohol	**2.70**	pyridine	**7.20**	2.58	0.83
[28]	tert-Butyl alcohol	**2.70**	diethyl ether	**5.30**	0.11	-0.74
[28]	tert-Butyl alcohol	**2.70**	toluene	**2.20**	-3.92	-4.13
[28]	phenol	**3.80**	Benzaldehyde	**4.80**	4.08	3.82
[28]	phenol	**3.80**	N,N-Dimethylformamide	**7.70**	11.04	10.71
[28]	phenol	**3.80**	tributylamine	**6.80**	8.88	8.20
[28]	phenol	**3.80**	pyrimidine	**5.40**	5.52	5.44
[28]	phenol	**3.80**	diethylamine	**7.90**	11.52	11.39
[28]	phenol	**3.80**	isopropylamine	**8.00**	11.76	11.52
[28]	phenol	**3.80**	aniline	**4.50**	3.36	3.46
[28]	phenol	**3.80**	Methyl ethanoate	**4.70**	3.84	5.18

[28]	phenol	**3.80**	Ethyl ethanoate	**5.40**	5.52	5.56
[28]	phenol	**3.80**	anisole	**3.30**	0.48	0.00
[28]	phenol	**3.80**	tetrahydrofuran	**5.90**	6.72	6.87
[28]	phenol	**3.80**	acetophenone	**5.50**	5.76	5.16
[29]	phenol	**3.80**	acetonitrile	**5.10**	4.80	4.89
[28]	phenol	**3.80**	benzonitrile	**4.80**	4.08	3.03
[28]	phenol	**3.80**	Tributylphosphine oxide	**10.70**	18.24	17.12
[28]	phenol	**3.80**	triphenylphosphine oxide	**10.10**	16.80	16.74
[28]	phenol	**3.80**	triethyl phosphate	**8.80**	13.68	13.34
[28]	phenol	**3.80**	cyclohexyl fluoride	**3.30**	0.48	-0.29
[28]	phenol	**3.80**	cyclohexyl chloride	**2.50**	-1.44	-2.09
[28]	phenol	**3.80**	cyclohexyl bromide	**2.50**	-1.44	-2.21
[28]	phenol	**3.80**	cyclohexyl iodide	**2.30**	-1.92	-2.33
[28]	4-fluorophenol	**3.90**	1-methyl-2-pyridone	**8.80**	14.50	13.58
[28]	4-fluorophenol	**3.90**	anisole	**3.30**	0.75	2.06
[28]	pentachlorophenol	**3.60**	triphenylphosphine oxide	**10.10**	14.90	15.87
[28]	pentachlorophenol	**3.60**	Propan-2-one	**5.70**	5.22	5.38
[28]	pentachlorophenol	**3.60**	triethyl phosphate	**8.80**	12.04	12.25
[31]	4-fluorophenol	**3.90**	triphenylphosphine oxide	**10.10**	17.75	17.12
[28]	4-fluorophenol	**3.90**	diphenyl sulphoxide	**7.50**	11.25	11.53
[28]	1-naphthol	**3.80**	triphenylphosphine oxide	**10.10**	16.80	17.20
[28]	Methanol	**2.90**	pyridine	**7.20**	3.90	2.72
[28]	Ethanol	**2.70**	Propan-2-one	**5.70**	0.63	0.45
[28]	2,2,2-trifluoroethanol	**3.70**	Ethyl ethanoate	**5.40**	5.04	4.79
[28]	Hexafluoropropan-2-ol	**4.50**	1,4-dioxane	**4.70**	6.71	8.84
[28]	tert-Butyl alcohol	**2.70**	Ethyl ethanoate	**5.40**	0.24	-0.43
[32]	4-fluorophenol	**3.90**	(Me2N)2C=NH	**10.20**	18.00	18.31
[28]	3-fluorophenol	**4.10**	pyridine	**7.20**	11.82	11.55
[28]	3-fluorophenol	**4.10**	dimethyl sulphoxide	**8.60**	15.60	15.24
[28]	3-fluorophenol	**4.10**	Ethyl ethanoate	**5.40**	6.96	7.31
[28]	Propan-1-ol	**2.60**	pyridine	**7.20**	1.92	1.46
[28]	tert-Butyl alcohol	**2.70**	Propan-2-one	**5.70**	0.63	0.00
[28]	4-Methoxyphenol	**3.70**	triethylamine	**7.50**	9.87	9.32
[28]	2-Isopropylphenol	**3.60**	tetrahydrofuran	**5.90**	5.66	5.15
[28]	2-tert-Butylphenol	**3.40**	N,N-dimethylacetamide	**8.50**	9.80	10.68
[28]	3-methylphenol	**3.70**	pyridine	**7.20**	9.18	8.94
[28]	2-Isopropylphenol	**3.60**	dibutyl ether	**5.00**	3.68	2.76
[28]	2-tert-Butylphenol	**3.40**	dibutyl ether	**5.00**	2.80	2.67
[28]	2-Methylphenol	**3.50**	dibutyl ether	**5.00**	3.24	2.40
[28]	2-Methylphenol	**3.50**	diethyl ether	**5.30**	3.87	3.07
[28]	2-Methylphenol	**3.50**	tetrahydrofuran	**5.90**	5.13	4.28
[28]	4-nitrophenol	**4.70**	benzene	**2.00**	-1.38	-1.70
[28]	4-nitrophenol	**4.70**	aniline	**4.50**	6.87	6.96
[28]	butan-1-ol	**2.70**	pyridine	**7.20**	2.58	2.37
[28]	3-trifluoromethylphenol	**4.30**	N,N-dimethylacetamide	**8.50**	16.91	16.46
[28]	phenol	**3.80**	tripropylamine	**6.60**	8.40	8.68

[28]	1-naphthol	**3.80**	benzene	**2.00**	-2.64	-2.90
[28]	1-naphthol	**3.80**	mesitylene	**2.70**	-0.96	-1.09
[1]	4-tert-Butylphenol	**3.60**	N,N-dimethylacetamide	**8.50**	11.38	10.83
[28]	2-naphthol	**3.90**	p-xylene	**2.40**	-1.50	-1.81
[1]	4-tert-Butylphenol	**3.60**	pyridine	**7.20**	8.52	10.61
[28]	2-naphthol	**3.90**	m-xylene	**2.40**	-1.50	-1.97
[28]	2-naphthol	**3.90**	o-xylene	**2.40**	-1.50	-1.93
[1]	3,5-dichlorophenol	**4.50**	triethylamine	**7.50**	15.39	15.47
[1]	3,5-dichlorophenol	**4.50**	aniline	**4.50**	6.09	6.20
[28]	2-naphthol	**3.90**	benzene	**2.00**	-2.50	-2.98
[36]	Ethanol	**2.70**	1-methyl-2-pyrrolidone	**8.30**	4.01	4.28
[35]	Methanol	**2.90**	triethylamine	**7.50**	4.35	3.96
[33]	Ethanol	**2.70**	triethylamine	**7.50**	2.97	2.61
[31]	4-fluorophenol	**3.90**	cyclohexyl fluoride	**3.30**	0.75	0.62
[37]	4-fluorophenol	**3.90**	Dichloromethane	**2.00**	-2.50	-2.85
[37]	4-fluorophenol	**3.90**	1,2-Dichloroethane	**2.40**	-1.50	-1.77
[37]	4-fluorophenol	**3.90**	cyclohexyl chloride	**2.50**	-1.25	-1.54
[37]	4-fluorophenol	**3.90**	cyclohexyl bromide	**2.50**	-1.25	-1.43
[37]	4-fluorophenol	**3.90**	cyclohexyl iodide	**2.30**	-1.75	-1.83
[38]	4-fluorophenol	**3.90**	benzene	**2.00**	-2.50	-2.80
[38]	4-fluorophenol	**3.90**	Octan-1-ol	**5.30**	5.75	5.93
[28]	4-fluorophenol	**3.90**	Chlorobenzene	**1.40**	-4.00	-4.54
[29]	4-fluorophenol	**3.90**	pyridine	**7.20**	10.50	10.73
[32]	4-fluorophenol	**3.90**	ammonia	**6.80**	9.50	9.58
[38]	4-fluorophenol	**3.90**	trimethylamine	**7.80**	12.00	12.15
[38]	4-fluorophenol	**3.90**	tripropylamine	**6.60**	9.00	8.39
[38]	4-fluorophenol	**3.90**	tributylamine	**6.80**	9.50	8.84
[31]	4-fluorophenol	**3.90**	1-methyl-2-pyrrolidone	**8.30**	13.25	14.02
[38]	4-fluorophenol	**3.90**	methanol	**4.80**	4.50	4.68
[38]	4-fluorophenol	**3.90**	Ethanol	**5.20**	5.50	5.82
[38]	4-fluorophenol	**3.90**	water	**4.50**	3.75	3.71
[32]	4-fluorophenol	**3.90**	pyrimidine	**5.40**	6.00	7.82
[2]	2,2,2-Trifluoro-1,1-bis(trifluoromethyl)ethanol	**4.90**	Tributylphosphine oxide	**10.70**	29.35	27.67
[36]	Butan-2-ol	**2.50**	1-methyl-2-pyrrolidone	**8.30**	2.47	3.71
[39]	4-fluorophenol	**3.90**	Tetramethylene sulfone	**6.30**	8.25	8.39
[28]	phenol	**3.80**	Diphenyl Sulfone	**5.90**	6.72	4.44
[39]	4-fluorophenol	**3.90**	Diphenyl Sulfone	**5.90**	7.25	6.90
[39]	4-fluorophenol	**3.90**	Dimethyl Sulfone	**6.20**	8.00	7.99
[28]	phenol	**3.80**	Dibutyl Sulfone	**6.40**	7.92	6.71
[39]	4-fluorophenol	**3.90**	Dibutyl Sulfone	**6.40**	8.50	8.67
[36]	4-bromophenol	**4.10**	1-methyl-2-pyrrolidone	**8.30**	14.79	15.18
[29]	4-chlorophenol	**4.10**	1-methyl-2-pyrrolidone	**8.30**	14.79	15.34
[36]	1-naphthol	**3.80**	1-methyl-2-pyrrolidone	**8.30**	12.48	13.18
[29]	4-Methoxyphenol	**3.70**	1-methyl-2-pyrrolidone	**8.30**	11.71	12.15
[29]	phenol	**3.80**	1-methyl-2-pyrrolidone	**8.30**	12.48	12.20
[36]	4-methylphenol	**3.70**	1-methyl-2-pyrrolidone	**8.30**	11.71	11.70

[36]	pentachlorophenol	**3.60**	1-methyl-2-pyrrolidone	**8.30**	10.94	11.58
[36]	Hexafluoropropan-2-ol	**4.50**	1-methyl-2-pyrrolidone	**8.30**	17.87	17.69
[36]	2,2,2-trifluoroethanol	**3.70**	1-methyl-2-pyrrolidone	**8.30**	11.71	11.30
[29]	phenol	**3.80**	Nitrobenzene	**3.70**	1.44	3.27
[29]	phenol	**3.80**	cyclohexanone	**6.20**	7.44	6.66
[29]	phenol	**3.80**	Trimethyl phosphate	**8.50**	12.96	12.53
[29]	phenol	**3.80**	2,6-dimethylpyridine	**7.80**	11.28	10.79
[29]	phenol	**3.80**	2,4,6-trimethylpyridine	**8.10**	12.00	11.66
[29]	phenol	**3.80**	1,1,3,3-Tetramethylurea	**8.50**	12.96	12.17
[29]	phenol	**3.80**	4-methylpyridine	**7.70**	11.04	10.39
[29]	4-fluorophenol	**3.90**	acetonitrile	**5.10**	5.25	5.13
[31]	4-fluorophenol	**3.90**	Ethyl ethanoate	**5.40**	6.00	6.52
[29]	4-fluorophenol	**3.90**	cyclohexanone	**6.20**	8.00	7.53
[29]	4-fluorophenol	**3.90**	diethyl ether	**5.30**	5.75	5.76
[31]	4-fluorophenol	**3.90**	Trimethyl phosphate	**8.50**	13.75	14.12
[31]	4-fluorophenol	**3.90**	tetrahydrofuran	**5.90**	7.25	7.42
[29]	4-fluorophenol	**3.90**	dimethyl sulphoxide	**8.60**	14.00	14.43
[40]	4-fluorophenol	**3.90**	1,1,3,3-Tetramethylurea	**8.50**	13.75	13.92
[29]	4-fluorophenol	**3.90**	N,N-dimethylaniline	**4.20**	3.00	2.57
[40]	4-fluorophenol	**3.90**	N,N-Dimethylformamide	**7.70**	11.75	11.98
[31]	4-fluorophenol	**3.90**	N,N-dimethylacetamide	**8.50**	13.75	13.82
[29]	4-fluorophenol	**3.90**	Hexamethylphosphoramide	**10.90**	19.75	20.31
[31]	4-fluorophenol	**3.90**	4-methylpyridine	**7.70**	11.75	11.92
[29]	4-fluorophenol	**3.90**	triethylamine	**7.50**	11.25	11.01
[29]	4-chlorophenol	**4.10**	Ethyl ethanoate	**5.40**	6.96	6.84
[29]	4-chlorophenol	**4.10**	Propan-2-one	**5.70**	7.77	7.90
[29]	4-chlorophenol	**4.10**	dimethyl sulphoxide	**8.60**	15.60	15.42
[29]	4-chlorophenol	**4.10**	1,1,3,3-Tetramethylurea	**8.50**	15.33	14.97
[29]	4-chlorophenol	**4.10**	N,N-Dimethylformamide	**7.70**	13.17	12.30
[29]	4-chlorophenol	**4.10**	Hexamethylphosphoramide	**10.90**	21.81	21.59
[29]	4-bromophenol	**4.10**	cyclohexanone	**6.20**	9.12	8.12
[29]	4-bromophenol	**4.10**	dimethyl sulphoxide	**8.60**	15.60	15.80
[29]	4-bromophenol	**4.10**	1,1,3,3-Tetramethylurea	**8.50**	15.33	15.15
[29]	4-bromophenol	**4.10**	N,N-dimethylacetamide	**8.50**	15.33	14.68
[29]	4-bromophenol	**4.10**	Hexamethylphosphoramide	**10.90**	21.81	22.39
[29]	4-bromophenol	**4.10**	pyridine	**7.20**	11.82	11.84
[29]	4-bromophenol	**4.10**	triethylamine	**7.50**	12.63	12.41
[29]	4-Iodophenol	**4.10**	dimethyl sulphoxide	**8.60**	15.60	16.12
[29]	4-Iodophenol	**4.10**	1,1,3,3-Tetramethylurea	**8.50**	15.33	15.16
[29]	4-Iodophenol	**4.10**	1-methyl-2-pyrrolidone	**8.30**	14.79	15.27
[29]	4-Iodophenol	**4.10**	Hexamethylphosphoramide	**10.90**	21.81	22.56
[29]	4-Iodophenol	**4.10**	pyridine	**7.20**	11.82	11.03
[29]	4-Iodophenol	**4.10**	triethylamine	**7.50**	12.63	12.96
[29]	3-methylphenol	**3.70**	cyclohexanone	**6.20**	6.88	5.98
[29]	3-methylphenol	**3.70**	dimethyl sulphoxide	**8.60**	12.40	12.41
[29]	3-methylphenol	**3.70**	1,1,3,3-Tetramethylurea	**8.50**	12.17	11.58

[28]	3-methylphenol	**3.70**	N,N-dimethylacetamide	**8.50**	12.17	11.30
[29]	3-methylphenol	**3.70**	1-methyl-2-pyrrolidone	**8.30**	11.71	11.96
[29]	3-methylphenol	**3.70**	Hexamethylphosphoramide	**10.90**	17.69	18.02
[29]	3-methylphenol	**3.70**	triethylamine	**7.50**	9.87	9.85
[29]	4-Methoxyphenol	**3.70**	Propan-2-one	**5.70**	5.73	5.63
[29]	4-Methoxyphenol	**3.70**	cyclohexanone	**6.20**	6.88	5.92
[29]	4-Methoxyphenol	**3.70**	dimethyl sulphoxide	**8.60**	12.40	12.41
[29]	4-Methoxyphenol	**3.70**	1,1,3,3-Tetramethylurea	**8.50**	12.17	11.60
[29]	4-Methoxyphenol	**3.70**	N,N-dimethylacetamide	**8.50**	12.17	11.25
[29]	4-Methoxyphenol	**3.70**	Hexamethylphosphoramide	**10.90**	17.69	17.90
[29]	4-Methoxyphenol	**3.70**	pyridine	**7.20**	9.18	8.81
[29]	4-nitrophenol	**4.70**	Nitrobenzene	**3.70**	4.23	4.08
[29]	4-nitrophenol	**4.70**	dimethyl sulphoxide	**8.60**	20.40	20.30
[29]	4-nitrophenol	**4.70**	1,1,3,3-Tetramethylurea	**8.50**	20.07	19.87
[28]	4-nitrophenol	**4.70**	N,N-dimethylacetamide	**8.50**	20.07	19.66
[29]	4-nitrophenol	**4.70**	1-methyl-2-pyrrolidone	**8.30**	19.41	18.01
[29]	Methanol	**2.90**	Ethyl ethanoate	**5.40**	1.20	0.84
[28]	Methanol	**2.90**	Trimethyl phosphate	**8.50**	5.85	5.86
[29]	Methanol	**2.90**	2,6-dimethylpyridine	**7.80**	4.80	2.88
[29]	Methanol	**2.90**	2,4,6-trimethylpyridine	**8.10**	5.25	2.97
[29]	Methanol	**2.90**	Hexamethylphosphoramide	**10.90**	9.45	9.08
[29]	Methanol	**2.90**	4-methylpyridine	**7.70**	4.65	2.80
[29]	Ethanol	**2.70**	Ethyl ethanoate	**5.40**	0.24	0.09
[29]	Ethanol	**2.70**	diethyl ether	**5.30**	0.11	-0.72
[29]	Ethanol	**2.70**	1,1,3,3-Tetramethylurea	**8.50**	4.27	4.18
[29]	Ethanol	**2.70**	N,N-Dimethylformamide	**7.70**	3.23	3.09
[29]	Ethanol	**2.70**	Hexamethylphosphoramide	**10.90**	7.39	8.47
[29]	Ethanol	**2.70**	pyridine	**7.20**	2.58	2.17
[29]	butan-1-ol	**2.70**	Propan-2-one	**5.70**	0.63	0.65
[29]	butan-1-ol	**2.70**	cyclohexanone	**6.20**	1.28	1.50
[28]	butan-1-ol	**2.70**	diethyl ether	**5.30**	0.11	-0.78
[29]	butan-1-ol	**2.70**	tetrahydrofuran	**5.90**	0.89	0.23
[28]	butan-1-ol	**2.70**	triethylamine	**7.50**	2.97	2.88
[29]	tert-Butyl alcohol	**2.70**	Hexamethylphosphoramide	**10.90**	7.39	6.87
[29]	2,2,2-trifluoroethanol	**3.70**	acetonitrile	**5.10**	4.35	4.24
[29]	2,2,2-trifluoroethanol	**3.70**	Trimethyl phosphate	**8.50**	12.17	12.38
[29]	2,2,2-trifluoroethanol	**3.70**	2,4,6-trimethylpyridine	**8.10**	11.25	10.16
[29]	2,2,2-trifluoroethanol	**3.70**	1,1,3,3-Tetramethylurea	**8.50**	12.17	11.42
[29]	2,2,2-trifluoroethanol	**3.70**	pyridine	**7.20**	9.18	8.83
[29]	Hexafluoropropan-2-ol	**4.50**	acetonitrile	**5.10**	7.95	8.80
[29]	Hexafluoropropan-2-ol	**4.50**	Ethyl ethanoate	**5.40**	8.88	8.95
[29]	Hexafluoropropan-2-ol	**4.50**	Propan-2-one	**5.70**	9.81	9.64
[29]	Hexafluoropropan-2-ol	**4.50**	diethyl ether	**5.30**	8.57	8.17
[29]	Hexafluoropropan-2-ol	**4.50**	tetrahydrofuran	**5.90**	10.43	10.78
[29]	Hexafluoropropan-2-ol	**4.50**	2,4,6-trimethylpyridine	**8.10**	17.25	17.32
[29]	Hexafluoropropan-2-ol	**4.50**	dimethyl sulphoxide	**8.60**	18.80	18.01

[29]	Hexafluoropropan-2-ol	**4.50**	1,1,3,3-Tetramethylurea	**8.50**	18.49	17.77
[29]	Hexafluoropropan-2-ol	**4.50**	N,N-dimethylacetamide	**8.50**	18.49	18.15
[29]	Hexafluoropropan-2-ol	**4.50**	Hexamethylphosphoramide	**10.90**	25.93	24.93
[29]	Hexafluoropropan-2-ol	**4.50**	pyridine	**7.20**	14.46	15.88
[31]	4-fluorophenol	**3.90**	quinuclidine	**9.10**	15.25	15.22
[28]	Methanol	**2.90**	tributylamine	**6.80**	3.30	3.46
[28]	Methanol	**2.90**	tripropylamine	**6.60**	3.00	3.00
[28]	Methanol	**2.90**	3-bromopyridine	**6.00**	2.10	1.45
[28]	Methanol	**2.90**	2-methylpyridine	**7.60**	4.50	2.76
[28]	Methanol	**2.90**	1,4-dioxane	**4.70**	0.15	1.00
[28]	Ethanol	**2.70**	1,4-dioxane	**4.70**	-0.67	0.13
[28]	2,2,2-trifluoroethanol	**3.70**	1,4-dioxane	**4.70**	3.43	4.60
[28]	Propan-1-ol	**2.60**	triethylamine	**7.50**	2.28	1.72
[28]	Butan-2-ol	**2.50**	triethylamine	**7.50**	1.59	1.30
[28]	Butan-2-ol	**2.50**	pyridine	**7.20**	1.26	1.87
[28]	phenol	**3.80**	dibutyl ether	**5.00**	4.56	4.56
[28]	phenol	**3.80**	tetrahydropyran	**5.80**	6.48	6.43
[28]	phenol	**3.80**	1,4-dioxane	**4.70**	3.84	3.89
[28]	phenol	**3.80**	n-hexylamine	**7.70**	11.04	10.73
[28]	4-chlorophenol	**4.10**	1,4-Diazabicyclo[2.2.2]octane	**8.90**	16.41	18.41
[31]	4-fluorophenol	**3.90**	tetrahydropyran	**5.80**	7.00	7.12
[41]	4-fluorophenol	**3.90**	dibutyl ether	**5.00**	5.00	5.02
[41]	4-fluorophenol	**3.90**	1,3-Dioxolane	**4.10**	2.75	2.57
[32]	4-fluorophenol	**3.90**	1-Methyl-1H-imidazole	**9.10**	15.25	15.52
[31]	4-fluorophenol	**3.90**	1-Methylpyrrolidine	**7.90**	12.25	12.85
[42]	4-fluorophenol	**3.90**	Dimethylcyanamide	**6.50**	8.75	8.90
[42]	4-fluorophenol	**3.90**	propionitrile	**5.20**	5.50	5.48
[31]	4-fluorophenol	**3.90**	chloroacetonitrile	**3.90**	2.25	2.42
[42]	4-fluorophenol	**3.90**	3,5-Dichloropyridine	**5.00**	5.00	4.85
[42]	4-fluorophenol	**3.90**	3-Chloropyridine	**6.00**	7.50	7.47
[42]	4-fluorophenol	**3.90**	3-Fluoropyridine	**6.10**	7.75	7.70
[42]	4-fluorophenol	**3.90**	2-methylpyridine	**7.60**	11.50	11.58
[31]	4-fluorophenol	**3.90**	3-methylpyridine	**7.50**	11.25	11.52
[42]	4-fluorophenol	**3.90**	Benzylamine	**7.20**	10.50	10.73
[31]	4-fluorophenol	**3.90**	N,N-Dimethylbenzamide	**8.00**	12.50	12.92
[31]	4-fluorophenol	**3.90**	Methyl ethanoate	**4.70**	4.25	5.92
[43]	4-fluorophenol	**3.90**	Nitrobenzene	**3.70**	1.75	1.71
[31]	4-fluorophenol	**3.90**	N,N-Dimethylthioacetamide	**6.00**	7.50	6.92
[44]	4-fluorophenol	**3.90**	cyclopentanone	**5.90**	7.25	7.25
[44]	4-fluorophenol	**3.90**	3-Methylbutan-2-one	**5.70**	6.75	6.85
[44]	4-fluorophenol	**3.90**	Propan-2-one	**5.70**	6.75	6.73
[44]	4-fluorophenol	**3.90**	Pentan-3-one	**5.60**	6.50	6.50
[44]	4-fluorophenol	**3.90**	acetophenone	**5.50**	6.25	6.33
[44]	4-fluorophenol	**3.90**	benzophenone	**5.40**	6.00	6.10
[45]	4-fluorophenol	**3.90**	2,4,6-trimethylpyridine	**8.10**	12.75	13.06
[31]	4-fluorophenol	**3.90**	2,6-dimethylpyridine	**7.80**	12.00	11.92

[31]	4-fluorophenol	**3.90**	2-Chloropyridine	**5.40**	6.00	6.12
[45]	4-fluorophenol	**3.90**	2-Fluoropyridine	**5.20**	5.50	5.42
[37]	4-fluorophenol	**3.90**	1,1,1-Trichloroethane	**1.50**	-3.75	-3.99
[46]	4-fluorophenol	**3.90**	isopropylamine	**8.00**	12.50	12.67
[31]	4-fluorophenol	**3.90**	2,2,2-Trifluoroethylamine	**4.60**	4.00	4.35
[32]	4-fluorophenol	**3.90**	p-xylene	**2.40**	-1.50	-1.71
[32]	4-fluorophenol	**3.90**	tetrahydrothiophene	**3.90**	2.25	1.71
[32]	4-fluorophenol	**3.90**	aniline	**4.50**	3.75	3.19
[31]	4-fluorophenol	**3.90**	pyridine N-oxide	**9.00**	15.00	15.52
[32]	4-fluorophenol	**3.90**	Triethylphosphine oxide	**10.10**	17.75	18.03
[28]	phenol	**3.80**	dibutyl sulphoxide	**8.70**	13.44	13.34
[3]	Propan-1-ol	**2.60**	Propan-1-ol	**5.30**	-0.36	0.55
[3]	propan-2-ol	**2.70**	Propan-2-ol	**5.50**	0.37	-0.67
[3]	butan-1-ol	**2.70**	cyclopentanone	**5.90**	0.89	1.07
[28]	phenol	**3.80**	cyclopentanone	**5.90**	6.72	6.69
[28]	phenol	**3.80**	Triethylphosphine oxide	**10.10**	16.80	18.83
[3]	phenol	**3.80**	Trimethylphosphine oxide	**10.70**	18.24	18.31
[28]	Methanol	**2.90**	triphenylphosphine oxide	**10.10**	8.25	7.53
[29]	Methanol	**2.90**	Diethyl ethylphosphonate	**9.20**	6.90	6.97
[29]	Methanol	**2.90**	Diethyl chloromethylphosphonate	**8.50**	5.85	5.82
[3]	Methanol	**2.90**	m-xylene	**2.40**	-3.30	-3.87
[3]	Methanol	**2.90**	mesitylene	**2.70**	-2.85	-3.54
[3]	Methanol	**2.90**	Chlorobenzene	**1.40**	-4.80	-4.54
[3]	2-Isopropylphenol	**3.60**	nitromethane	**3.70**	0.82	0.45
[3]	Methanol	**2.90**	nitromethane	**3.70**	-1.35	-3.99
[3]	2-Isopropylphenol	**3.60**	acetophenone	**5.50**	4.78	4.68
[3]	2-Isopropylphenol	**3.60**	benzophenone	**5.40**	4.56	3.67
[3]	2-Isopropylphenol	**3.60**	diethyl ether	**5.30**	4.34	3.37
[3]	2-Isopropylphenol	**3.60**	1,4-dioxane	**4.70**	3.02	4.56
[35]	Methanol	**2.90**	benzene	**2.00**	-3.90	-4.21
[35]	Methanol	**2.90**	Ethylamine	**7.90**	4.95	4.71
[35]	Methanol	**2.90**	Methylamine	**7.80**	4.80	4.38
[35]	Methanol	**2.90**	diethylamine	**7.90**	4.95	4.41
[35]	Methanol	**2.90**	trimethylamine	**7.80**	4.80	3.62
[35]	Methanol	**2.90**	quinuclidine	**9.10**	6.75	5.74
[35]	Methanol	**2.90**	3,5-Dichloropyridine	**5.00**	0.60	-1.02
[35]	Methanol	**2.90**	3-Fluoropyridine	**6.10**	2.25	0.85
[35]	Methanol	**2.90**	3,5-Dimethylpyridine	**8.00**	5.10	3.64
[35]	Methanol	**2.90**	1,3-Dioxolane	**4.10**	-0.75	-0.51
[35]	Methanol	**2.90**	tetrahydrofuran	**5.90**	1.95	0.75
[35]	Methanol	**2.90**	tetrahydrothiophene	**3.90**	-1.05	-2.00
[35]	Methanol	**2.90**	cyclohexyl fluoride	**3.30**	-1.95	-2.37
[35]	Methanol	**2.90**	cyclohexyl chloride	**2.50**	-3.15	-2.80
[31]	4-fluorophenol	**3.90**	dibutyl sulphoxide	**8.70**	14.25	15.42
[31]	4-fluorophenol	**3.90**	toluene	**2.20**	-2.00	-1.98
[31]	4-fluorophenol	**3.90**	mesitylene	**2.70**	-0.75	-1.28

[31]	4-fluorophenol	**3.90**	Hexamethylbenzene	**3.10**	0.25	0.02
[31]	4-fluorophenol	**3.90**	n-butylamine	**8.00**	12.50	12.32
[31]	4-fluorophenol	**3.90**	di-n-butylamine	**7.90**	12.25	12.12
[31]	4-fluorophenol	**3.90**	diethylamine	**7.90**	12.25	12.72
[31]	4-fluorophenol	**3.90**	1,4-Diazabicyclo[2.2.2]octane	**8.90**	14.75	13.12
[31]	4-fluorophenol	**3.90**	cyclohexyldimethylamine	**7.80**	12.00	12.22
[31]	4-fluorophenol	**3.90**	3,5-Dimethylpyridine	**8.00**	12.50	12.42
[31]	4-fluorophenol	**3.90**	2,2,5,5-tetramethyltetrahydrofuran	**6.20**	8.00	8.32
[31]	4-fluorophenol	**3.90**	Ethyl formate	**4.50**	3.75	4.32
[31]	4-fluorophenol	**3.90**	Methyl formate	**4.50**	3.75	3.62
[31]	4-fluorophenol	**3.90**	nitromethane	**3.70**	1.75	0.02
[31]	4-fluorophenol	**3.90**	Trimethylphosphine oxide	**10.70**	19.25	19.92
[31]	4-fluorophenol	**3.90**	Tributylphosphine oxide	**10.70**	19.25	20.72
[31]	4-fluorophenol	**3.90**	dimethyl sulphide	**3.50**	1.25	0.72
[31]	4-fluorophenol	**3.90**	dibutyl sulphide	**3.60**	1.50	1.12
[31]	4-fluorophenol	**3.90**	1-Fluoropentane	**2.90**	-0.25	0.02
[31]	4-fluorophenol	**3.90**	1-Chloropentane	**2.20**	-2.00	-1.88
[31]	4-fluorophenol	**3.90**	1-Bromopentane	**2.30**	-1.75	-1.98
[31]	4-fluorophenol	**3.90**	1-Iodopentane	**2.50**	-1.25	-3.78
[28]	Methanol	**2.90**	benzophenone	**5.40**	1.20	1.00
[28]	Ethanol	**2.70**	benzophenone	**5.40**	0.24	0.46
[28]	2,2,2-trifluoroethanol	**3.70**	tetrahydrofuran	**5.90**	6.19	5.86
[28]	Propan-1-ol	**2.60**	diethyl ether	**5.30**	-0.36	-0.25
[28]	Propan-1-ol	**2.60**	Propan-2-one	**5.70**	0.12	-1.72
[28]	Propan-1-ol	**2.60**	benzophenone	**5.40**	-0.24	-0.13
[28]	butan-1-ol	**2.70**	1,4-Diazabicyclo[2.2.2]octane	**8.90**	4.79	5.23
[28]	tert-Butyl alcohol	**2.70**	benzophenone	**5.40**	0.24	0.04
[28]	phenol	**3.80**	benzophenone	**5.40**	5.52	5.02
[28]	phenol	**3.80**	3-Methylbutan-2-one	**5.70**	6.24	5.44
[28]	phenol	**3.80**	propionitrile	**5.20**	5.04	5.40
[28]	phenol	**3.80**	chloroacetonitrile	**3.90**	1.92	1.72
[28]	phenol	**3.80**	Dimethylcyanamide	**6.50**	8.16	6.28
[28]	phenol	**3.80**	Diethyl chloromethylphosphonate	**8.50**	12.96	12.97
[28]	phenol	**3.80**	Diethyl isopropylphosphonate	**9.10**	14.40	14.64
[28]	phenol	**3.80**	Diethyl ethylphosphonate	**9.20**	14.64	14.23
[28]	phenol	**3.80**	toluene	**2.20**	-2.16	-1.86
[28]	phenol	**3.80**	o-xylene	**2.40**	-1.68	-1.97
[28]	phenol	**3.80**	p-xylene	**2.40**	-1.68	-1.89
[28]	phenol	**3.80**	mesitylene	**2.70**	-0.96	-1.39
[28]	phenol	**3.80**	Hexamethylbenzene	**3.10**	0.00	-0.13
[28]	4-methylphenol	**3.70**	Hexamethylbenzene	**3.10**	-0.25	-0.17
[28]	phenol	**3.80**	Tetramethylene sulfone	**6.30**	7.68	7.11
[28]	phenol	**3.80**	1,4-Diazabicyclo[2.2.2]octane	**8.90**	13.92	14.02
[28]	propan-2-ol	**2.70**	2-methylpyridine	**7.60**	3.10	0.50
[28]	propan-2-ol	**2.70**	3-methylpyridine	**7.50**	2.97	0.46
[28]	propan-2-ol	**2.70**	4-methylpyridine	**7.70**	3.23	0.46

[28]	propan-2-ol	**2.70**	2-Chloropyridine	**5.40**	0.24	-2.38
[28]	propan-2-ol	**2.70**	2-Fluoropyridine	**5.20**	-0.02	-2.51
[28]	propan-2-ol	**2.70**	3-Chloropyridine	**6.00**	1.02	-1.84
[28]	propan-2-ol	**2.70**	2,6-dimethylpyridine	**7.80**	3.36	0.54
[28]	propan-2-ol	**2.70**	3,5-Dimethylpyridine	**8.00**	3.62	0.59
[28]	propan-2-ol	**2.70**	2,4,6-trimethylpyridine	**8.10**	3.75	0.92
[28]	4-fluorophenol	**3.90**	Cyclopropylamine	**6.90**	9.75	9.37
[28]	3-fluorophenol	**4.10**	N,N-dimethylacetamide	**8.50**	15.33	14.64
[16]	4-methylphenol	**3.70**	triethylamine	**7.50**	9.87	9.72
[28]	4-methylphenol	**3.70**	N,N-dimethylacetamide	**8.50**	12.17	10.88
[16]	4-methylphenol	**3.70**	pyridine	**7.20**	9.18	8.80
[16]	4-methylphenol	**3.70**	aniline	**4.50**	2.97	2.48
[28]	4-methylphenol	**3.70**	benzene	**2.00**	-2.78	-3.26
[28]	4-methylphenol	**3.70**	toluene	**2.20**	-2.32	-2.89
[28]	4-methylphenol	**3.70**	m-xylene	**2.40**	-1.86	-2.26
[28]	4-methylphenol	**3.70**	o-xylene	**2.40**	-1.86	-2.22
[28]	4-methylphenol	**3.70**	p-xylene	**2.40**	-1.86	-2.05
[28]	4-methylphenol	**3.70**	mesitylene	**2.70**	-1.17	-1.63
[28]	3-nitrophenol	**4.60**	N,N-dimethylacetamide	**8.50**	19.28	18.41
[28]	2-Methylphenol	**3.50**	benzene	**2.00**	-3.06	-3.39
[28]	2-Methylphenol	**3.50**	mesitylene	**2.70**	-1.59	-1.72
[28]	1-naphthol	**3.80**	p-xylene	**2.40**	-1.68	-1.68
[28]	2-tert-Butylphenol	**3.40**	N-Methylacetamide	**8.20**	9.20	9.62
[28]	2-tert-Butylphenol	**3.40**	tetrahydrofuran	**5.90**	4.60	5.02
[28]	4-chlorophenol	**4.10**	dibutyl ether	**5.00**	5.88	6.28
[28]	4-chlorophenol	**4.10**	benzophenone	**5.40**	6.96	6.69
[28]	4-chlorophenol	**4.10**	dibutyl sulphide	**3.60**	2.10	2.51
[28]	4-Methoxyphenol	**3.70**	benzophenone	**5.40**	5.04	5.02
[28]	pentachlorophenol	**3.60**	N,N-Dimethylformamide	**7.70**	9.62	9.62
[28]	pentachlorophenol	**3.60**	N,N-diethylacetamide	**8.50**	11.38	11.72
[28]	pentachlorophenol	**3.60**	1-methyl-2-pyridone	**8.80**	12.04	12.13
[28]	pentachlorophenol	**3.60**	pyridine	**7.20**	8.52	11.30
[28]	pentachlorophenol	**3.60**	2,4,6-trimethylpyridine	**8.10**	10.50	12.97
[28]	pentachlorophenol	**3.60**	tetrahydropyran	**5.80**	5.44	4.60
[28]	pentachlorophenol	**3.60**	Ethyl ethanoate	**5.40**	4.56	3.77
[28]	pentachlorophenol	**3.60**	cyclohexanone	**6.20**	6.32	5.44
[28]	pentachlorophenol	**3.60**	Trimethylphosphine oxide	**10.70**	16.22	17.15
[28]	pentachlorophenol	**3.60**	Diethyl chloromethylphosphonate	**8.50**	11.38	11.30
[28]	pentachlorophenol	**3.60**	Trimethyl phosphate	**8.50**	11.38	11.30
[28]	pentachlorophenol	**3.60**	Diethyl ethylphosphonate	**9.20**	12.92	12.97
[47]	1-naphthol	**3.80**	Trimethylphosphine oxide	**10.70**	18.24	18.71
[28]	phenol	**3.80**	quinoline	**7.30**	10.08	9.85
[28]	phenol	**3.80**	tert-butylamine	**8.10**	12.00	11.24
[28]	Pentafluorophenol	**4.50**	triphenylphosphine oxide	**10.10**	23.45	21.55
[1]	3,4-dichlorophenol	**4.40**	triethylamine	**7.50**	14.70	14.16
[1]	4-cyanophenol	**4.60**	triethylamine	**7.50**	16.08	15.57

[28]	4-fluorophenol	**3.90**	Pyridazine	**6.70**	9.25	9.41
[38]	4-fluorophenol	**3.90**	Pyrazine	**5.10**	5.25	5.25
[36]	3-Isopropylphenol	**3.70**	1-methyl-2-pyrrolidone	**8.30**	11.71	11.81
[36]	Cyclohexanol	**2.60**	1-methyl-2-pyrrolidone	**8.30**	3.24	3.82
[39]	4-fluorophenol	**3.90**	N,N-Dimethylmethanesulfonamide	**5.95**	7.37	7.42
[36]	benzyl alcohol	**3.00**	1-methyl-2-pyrrolidone	**8.30**	6.32	5.88
[41]	4-fluorophenol	**3.90**	1,2-Dimethoxyethane	**5.30**	5.75	5.82
[42]	4-fluorophenol	**3.90**	piperidine	**8.30**	13.25	13.58
[42]	4-fluorophenol	**3.90**	N-methylpiperidine	**7.70**	11.75	12.04
[42]	4-fluorophenol	**3.90**	quinoline	**7.30**	10.75	10.78
[42]	4-fluorophenol	**3.90**	tert-butylamine	**8.10**	12.75	12.49
[34]	4-fluorophenol	**3.90**	N,N-Diethylformamide	**7.70**	11.75	11.87
[44]	4-fluorophenol	**3.90**	2,4-Dimethyl-3-pentanone	**5.50**	6.25	6.16
[44]	4-fluorophenol	**3.90**	3,3-Dimethyl-2-butanone	**5.70**	6.75	6.68
[48]	4-fluorophenol	**3.90**	3,4-Dimethylpyridine	**8.00**	12.50	12.78
[48]	4-fluorophenol	**3.90**	4-Methoxypyridine	**7.80**	12.00	12.15
[48]	4-fluorophenol	**3.90**	2-Methoxypyridine	**5.30**	5.75	5.65
[48]	4-fluorophenol	**3.90**	2-Cyanopyridine	**5.00**	5.00	4.85
[46]	4-fluorophenol	**3.90**	2-Propen-1-amine	**7.40**	11.00	11.07
[32]	4-fluorophenol	**3.90**	morpholine	**7.20**	10.50	10.61
[35]	Methanol	**2.90**	Dimethylamine	**8.10**	5.25	4.44
[31]	4-fluorophenol	**3.90**	c-hexylamine	**8.10**	12.75	13.22
[31]	4-fluorophenol	**3.90**	Dimethylamine	**8.10**	12.75	12.62
[31]	4-fluorophenol	**3.90**	N-Methylformamide	**7.40**	11.00	10.22
[28]	propan-2-ol	**2.70**	3,4-Dimethylpyridine	**8.00**	3.62	0.50
[28]	4-cyanophenol	**4.60**	N,N-dimethylacetamide	**8.50**	19.28	18.41
[49]	4-phenylazophenol	**4.30**	Tributylphosphine oxide	**10.70**	23.29	22.61
[49]	4-nitrophenol	**4.70**	Tributylphosphine oxide	**10.70**	27.33	28.49
[47]	1-naphthol	**3.80**	Tri-cyclohexylphosphine oxide	**11.30**	19.68	21.29
[47]	1-naphthol	**3.80**	Tri-n-octylphosphine oxide	**11.30**	19.68	19.84
[47]	1-naphthol	**3.80**	Tributylphosphine oxide	**10.70**	18.24	19.39
[47]	1-naphthol	**3.80**	triethyl phosphate	**8.80**	13.68	14.44
[47]	1-naphthol	**3.80**	dibutyl sulphoxide	**8.70**	13.44	14.05
[47]	1-naphthol	**3.80**	N,N-di-n-hexylacetamide	**8.40**	12.72	13.36
[3]	tert-Butyl alcohol	**2.70**	toluene	**2.20**	-3.92	-4.13
[3]	tert-Butyl alcohol	**2.70**	o-xylene	**2.40**	-3.66	-4.72
[3]	tert-Butyl alcohol	**2.70**	m-xylene	**2.40**	-3.66	-3.55
[3]	tert-Butyl alcohol	**2.70**	p-xylene	**2.40**	-3.66	-4.00
[3]	tert-Butyl alcohol	**2.70**	mesitylene	**2.70**	-3.27	-3.35
[3]	tert-Butyl alcohol	**2.70**	Hexamethylbenzene	**3.10**	-2.75	-2.47

f) Dichloromethane

Solvent		α_S	C_α	β_S	C_β
CH2Cl2		1.80	2.16	1.40	1.76

Figure S4f.1 Dichloromethane: All donors

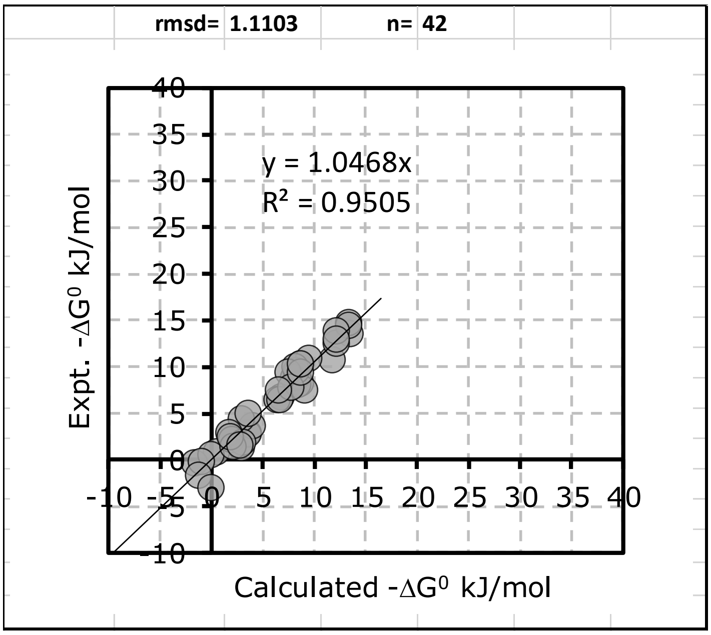

Free energy calculation for 1:1 Association

$$-\Delta G^0 = \alpha\beta - \alpha\beta_S - \alpha_S\beta - C_\alpha - C_\beta$$

Figure S4f.2 Dichloromethane: H-bond acceptors with 4-fluorophenol as acceptor

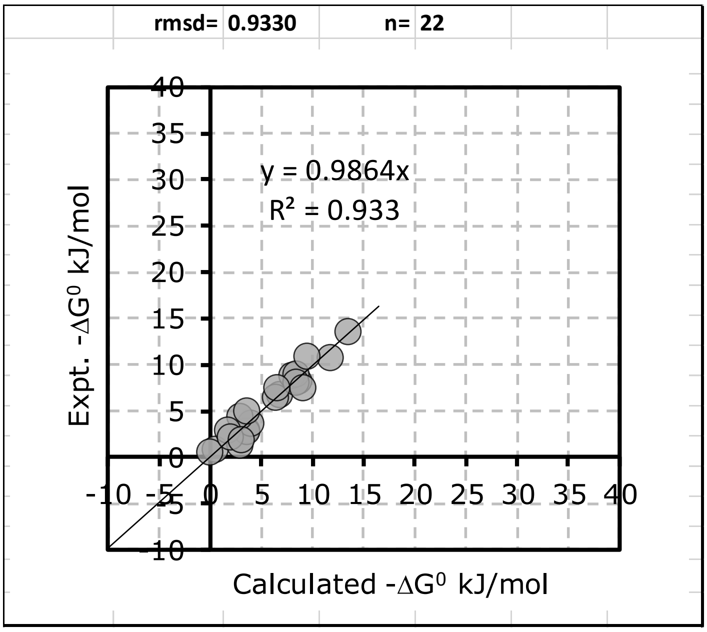

Free energy calculation for 1:1 Association

$$-\Delta G^0 = \alpha\beta - \alpha\beta_S - \alpha_S\beta - C_\alpha - C_\beta$$

Table S4f	Donor		Acceptor		Calc.	Expt.
Reference	DH	Alpha	A	Beta	$-\Delta G^0$	$-\Delta G^0$
[1]	4-fluorophenol	**3.90**	1,4-dioxane	**4.70**	0.49	0.83
[1]	phenol	**3.80**	pyridine N-oxide	**9.00**	8.76	10.27
[1]	4-fluorophenol	**3.90**	Hexamethylphosphoramide	**10.90**	13.51	13.52

[1]	4-fluorophenol	**3.90**	N,N-Dimethylformamide	**7.70**	6.79	6.71
[1]	4-fluorophenol	**3.90**	dimethyl sulphoxide	**8.60**	8.68	8.23
[1]	4-fluorophenol	**3.90**	cyclohexanone	**6.20**	3.64	2.88
[1]	4-fluorophenol	**3.90**	triphenylphosphine oxide	**10.10**	11.83	10.68
[1]	4-fluorophenol	**3.90**	diphenyl sulphoxide	**7.50**	6.37	6.36
[36]	2,2,2-trifluoroethanol	**3.70**	1-methyl-2-pyrrolidone	**8.30**	6.67	6.33
[36]	4-fluorophenol	**3.90**	1-methyl-2-pyrrolidone	**8.30**	8.05	8.84
[36]	Hexafluoropropan-2-ol	**4.50**	1-methyl-2-pyrrolidone	**8.30**	12.19	12.49
[36]	Butan-2-ol	**2.50**	1-methyl-2-pyrrolidone	**8.30**	-1.61	-0.34
[39]	4-fluorophenol	**3.90**	Diphenyl Sulfone	**5.90**	3.01	1.26
[39]	4-fluorophenol	**3.90**	Dimethyl Sulfone	**6.20**	3.64	2.68
[39]	4-fluorophenol	**3.90**	Dibutyl Sulfone	**6.40**	4.06	3.59
[29]	4-fluorophenol	**3.90**	tetrahydrofuran	**5.90**	3.01	4.29
[29]	4-fluorophenol	**3.90**	diethyl ether	**5.30**	1.75	2.83
[29]	4-fluorophenol	**3.90**	2,2,5,5-tetramethyltetrahydrofuran	**6.20**	3.64	4.97
[29]	4-fluorophenol	**3.90**	Ethyl formate	**4.50**	0.07	0.47
[29]	4-fluorophenol	**3.90**	Ethyl ethanoate	**5.40**	1.96	2.18
[29]	4-fluorophenol	**3.90**	N,N-dimethylacetamide	**8.50**	8.47	8.97
[29]	4-fluorophenol	**3.90**	pyridine N-oxide	**9.00**	9.52	10.87
[29]	4-fluorophenol	**3.90**	Trimethyl phosphate	**8.50**	8.47	7.98
[29]	4-fluorophenol	**3.90**	triethyl phosphate	**8.80**	9.10	7.51
[36]	Cyclohexanol	**2.60**	1-methyl-2-pyrrolidone	**8.30**	-0.92	-0.23
[39]	4-fluorophenol	**3.90**	N,N-Dimethylmethanesulfonamide	**5.95**	3.11	1.83
[18]	3,5,5-Trimethyl-hexanoic acid phenylamide	**2.90**	Diethyl ethylphosphonate	**9.20**	2.14	1.31
[50]	Pentafluorophenol	**4.50**	water	**4.50**	1.93	2.40
[47]	1-naphthol	**3.80**	Tri-cyclohexylphosphine oxide	**11.30**	13.36	14.65
[47]	1-naphthol	**3.80**	Tri-n-octylphosphine oxide	**11.30**	13.36	14.44
[47]	1-naphthol	**3.80**	Tributylphosphine oxide	**10.70**	12.16	13.78
[47]	1-naphthol	**3.80**	Trimethylphosphine oxide	**10.70**	12.16	12.87
[47]	1-naphthol	**3.80**	triethyl phosphate	**8.80**	8.36	9.54
[47]	1-naphthol	**3.80**	dibutyl sulphoxide	**8.70**	8.16	9.93
[47]	1-naphthol	**3.80**	N,N-di-n-hexylacetamide	**8.40**	7.56	9.32
[6]	1-naphthol	**3.80**	1,1,3,3-Tetramethylurea	**8.50**	7.76	7.70
[51]	Indole	**3.10**	N,N-diethylacetamide	**8.50**	2.79	1.49
[51]	Indole	**3.10**	Ethyl ethanoate	**5.40**	-1.24	-1.76
[51]	tert-Butyl alcohol	**2.70**	N,N-diethylacetamide	**8.50**	-0.05	-3.06
[52]	4-fluorophenol	**3.90**	N,N-Dicyclohexyl-2,2-dimethylpropionamide	**7.60**	6.58	7.48
[6]	1-naphthol	**3.80**	pyridine N-oxide	**9.00**	8.76	9.35
[28]	phenol	**3.80**	pyridine N-oxide	**9.00**	8.76	10.25

g) Chloroform

Solvent		α_S	C_α	β_S	C_β
**CHCl3**		2.10	1.78	1.30	2.11

Figure S4g

	rmsd=	1.2277	n=	13

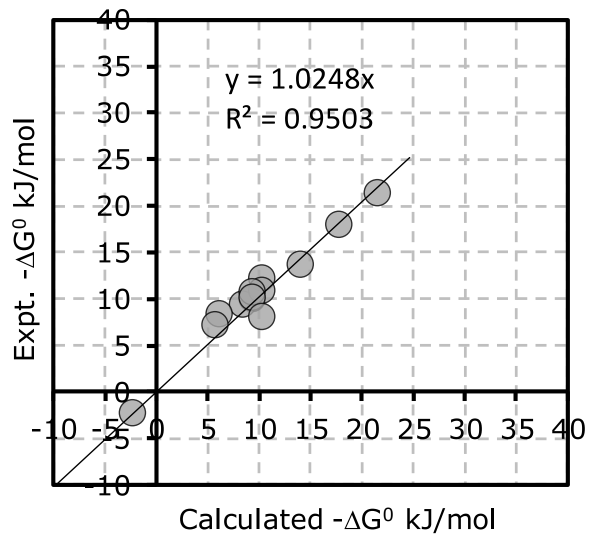

Free energy calculation for 1:1 Association

$$-\Delta G^0 = \alpha\beta - \alpha\beta_S - \alpha_S\beta - C_\alpha - C_\beta$$

**Table S4g**	**Donor**		**Acceptor**		**Calc.**	**Expt.**
**Reference**	**DH**	**Alpha**	**A**	**Beta**	**-ΔG⁰**	**-ΔG⁰**
[49]	4-phenylazophenol	**4.30**	Tributylphosphine oxide	**10.70**	14.06	13.58
[49]	4-nitrophenol	**4.70**	Tributylphosphine oxide	**10.70**	17.82	17.95
[49]	3-trifluoromethyl-4-nitrophenol	**5.10**	Tributylphosphine oxide	**10.70**	21.58	21.29
[53]	phenol	**3.80**	Tributylphosphine oxide	**10.70**	9.36	9.90
[53]	4-Methoxyphenol	**3.70**	Tributylphosphine oxide	**10.70**	8.42	9.30
[47]	1-naphthol	**3.80**	Tri-cyclohexylphosphine oxide	**11.30**	10.38	12.17
[47]	1-naphthol	**3.80**	Tri-n-octylphosphine oxide	**11.30**	10.38	10.89
[47]	1-naphthol	**3.80**	Tributylphosphine oxide	**10.70**	9.36	10.76
[47]	1-naphthol	**3.80**	Trimethylphosphine oxide	**10.70**	9.36	10.06
[47]	1-naphthol	**3.80**	triethyl phosphate	**8.80**	6.13	8.34
[9]	phenol	**3.80**	dimethyl sulphoxide	**8.60**	5.79	7.15
[51]	tert-Butyl alcohol	**2.70**	N,N-diethylacetamide	**8.50**	-2.30	-2.33
[6]	phenol	**3.80**	Tri-n-octylphosphine oxide	**11.30**	10.38	8.03

h) 1,2-Dichloroethane

Solvent	α_S	C_α	β_S	C_β
**CH2Cl2CH2Cl2**	1.70	2.23	1.60	1.41

Figure S4h

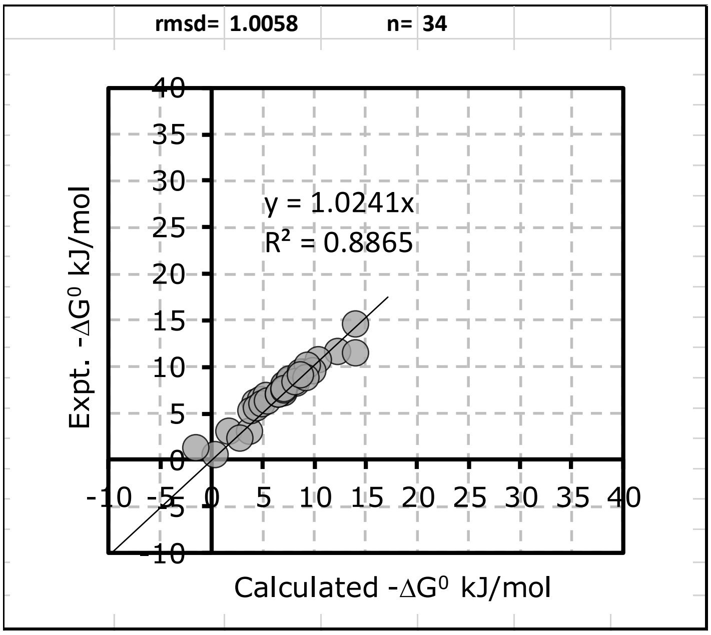

Free energy calculation for 1:1 Association

$$-\Delta G^0 = \alpha\beta - \alpha\beta_S - \alpha_S\beta - C_\alpha - C_\beta$$

Table S4h	Donor		Acceptor		Calc.	Expt.
**Reference**	**DH**	**Alpha**	**A**	**Beta**	**-ΔG⁰**	**-ΔG⁰**
[1]	4-fluorophenol	**3.90**	1,4-dioxane	**4.70**	0.46	0.45
[1]	4-fluorophenol	**3.90**	Hexamethylphosphoramide	**10.90**	14.10	14.55
[1]	4-fluorophenol	**3.90**	N,N-Dimethylformamide	**7.70**	7.06	7.30
[1]	3-fluorophenol	**4.10**	dibutyl ether	**5.00**	1.80	2.96
[1]	4-fluorophenol	**3.90**	dimethyl sulphoxide	**8.60**	9.04	9.43
[1]	4-fluorophenol	**3.90**	cyclohexanone	**6.20**	3.76	2.96
[1]	4-fluorophenol	**3.90**	triphenylphosphine oxide	**10.10**	12.34	11.65
[1]	3-fluorophenol	**4.10**	dimethyl sulphoxide	**8.60**	10.44	10.63
[1]	3-fluorophenol	**4.10**	Ethyl ethanoate	**5.40**	2.76	2.27
[9]	2-Methoxyphenol	**2.40**	dimethyl sulphoxide	**8.60**	-1.46	1.26
[9]	phenol	**3.80**	dimethyl sulphoxide	**8.60**	8.34	8.19
[54]	3,4-Dimethylphenol	**3.60**	3-Methyl-4-pyrimidone	**7.20**	4.28	6.12
[54]	4-Methoxyphenol	**3.70**	3-Methyl-4-pyrimidone	**7.20**	4.84	6.34
[54]	phenol	**3.80**	3-Methyl-4-pyrimidone	**7.20**	5.40	6.82
[54]	3-fluorophenol	**4.10**	3-Methyl-4-pyrimidone	**7.20**	7.08	7.09
[54]	4-chlorophenol	**4.10**	3-Methyl-4-pyrimidone	**7.20**	7.08	7.90
[54]	4-bromophenol	**4.10**	3-Methyl-4-pyrimidone	**7.20**	7.08	8.00
[54]	3-Chlorophenol	**4.20**	3-Methyl-4-pyrimidone	**7.20**	7.64	8.50
[54]	3-Bromophenol	**4.20**	3-Methyl-4-pyrimidone	**7.20**	7.64	8.61
[54]	3,4-dichlorophenol	**4.40**	3-Methyl-4-pyrimidone	**7.20**	8.76	9.42
[54]	3-nitrophenol	**4.60**	3-Methyl-4-pyrimidone	**7.20**	9.88	9.54
[54]	3,5-dichlorophenol	**4.50**	3-Methyl-4-pyrimidone	**7.20**	9.32	10.14

[54]	3,4-Dimethylphenol	**3.60**	1,3-Dimethyluracil	**7.00**	3.90	5.28
[54]	4-Methoxyphenol	**3.70**	1,3-Dimethyluracil	**7.00**	4.44	5.46
[54]	phenol	**3.80**	1,3-Dimethyluracil	**7.00**	4.98	5.91
[54]	4-fluorophenol	**3.90**	1,3-Dimethyluracil	**7.00**	5.52	6.22
[54]	4-chlorophenol	**4.10**	1,3-Dimethyluracil	**7.00**	6.60	6.95
[54]	4-bromophenol	**4.10**	1,3-Dimethyluracil	**7.00**	6.60	7.02
[54]	3-Chlorophenol	**4.20**	1,3-Dimethyluracil	**7.00**	7.14	7.40
[54]	3-Bromophenol	**4.20**	1,3-Dimethyluracil	**7.00**	7.14	7.54
[54]	3,4-dichlorophenol	**4.40**	1,3-Dimethyluracil	**7.00**	8.22	8.38
[54]	3-nitrophenol	**4.60**	1,3-Dimethyluracil	**7.00**	9.30	8.79
[54]	3,5-dichlorophenol	**4.50**	1,3-Dimethyluracil	**7.00**	8.76	9.04
[6]	phenol	**3.80**	Tri-n-octylphosphine oxide	**11.30**	14.01	11.45

i) Chlorobenzene

Solvent	α_S	C_α	β_S	C_β
Chlorobenzene	1.40	2.51	1.40	1.71

Figure S4i

rmsd= 1.5044 n= 22

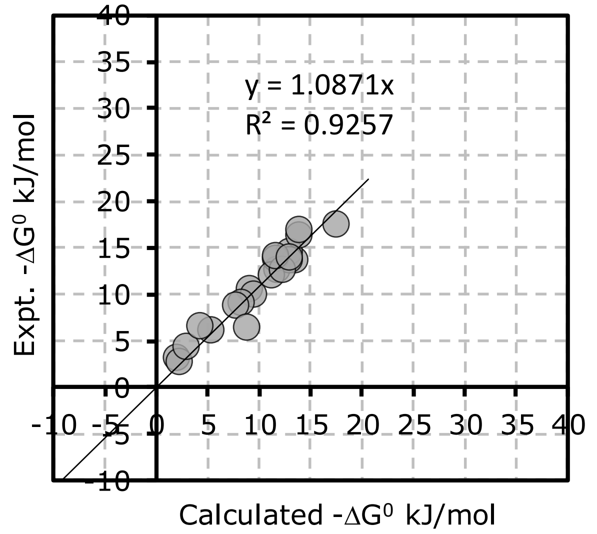

Free energy calculation for 1:1 Association

$$-\Delta G^0 = \alpha\beta - \alpha\beta_S - \alpha_S\beta - C_\alpha - C_\beta$$

Table S4i	Donor		Acceptor		Calc.	Expt.
Reference	DH	Alpha	A	Beta	$-\Delta G^0$	$-\Delta G^0$
[1]	4-fluorophenol	**3.90**	triethylamine	**7.50**	9.07	10.49
[1]	4-fluorophenol	**3.90**	1,4-dioxane	**4.70**	2.07	3.10
[1]	4-fluorophenol	**3.90**	Hexamethylphosphoramide	**10.90**	17.57	17.46
[1]	4-fluorophenol	**3.90**	N,N-Dimethylformamide	**7.70**	9.57	9.93
[1]	4-fluorophenol	**3.90**	benzonitrile	**4.80**	2.32	2.72
[1]	4-fluorophenol	**3.90**	4-N,N-dimethylaminopyridine	**9.30**	13.57	13.58
[1]	4-fluorophenol	**3.90**	pyridine	**7.20**	8.32	9.14
[1]	4-fluorophenol	**3.90**	3-bromopyridine	**6.00**	5.32	6.16
[1]	4-fluorophenol	**3.90**	dimethyl sulphoxide	**8.60**	11.82	12.57
[1]	Methanol	**2.90**	triethylamine	**7.50**	2.97	4.36
[5]	4-nitrophenol	**4.70**	Benzylamine	**7.20**	12.96	14.50
[5]	4-nitrophenol	**4.70**	pyridine	**7.20**	12.96	13.68
[5]	4-nitrophenol	**4.70**	tributylamine	**6.80**	11.64	13.81
[5]	4-nitrophenol	**4.70**	triethylamine	**7.50**	13.95	16.32
[6]	phenol	**3.80**	pyridine	**7.20**	7.74	8.84
[6]	3,4-dichlorophenol	**4.40**	pyridine	**7.20**	11.22	12.00
[6]	3-nitrophenol	**4.60**	pyridine	**7.20**	12.38	12.64
[6]	2-Chlorophenol	**4.00**	pyridine	**7.20**	8.90	6.36
[6]	2,6-Dichlorophenol	**3.20**	pyridine	**7.20**	4.26	6.56
[6]	4-nitrophenol-D	**4.70**	triethylamine	**7.50**	13.95	16.89
[6]	4-nitrophenol-D	**4.70**	tributylamine	**6.80**	11.64	14.09
[6]	4-nitrophenol-D	**4.70**	pyridine	**7.20**	12.96	13.97

j) Perfluorohexane

Solvent	α_S	C_α	β_S	C_β
**Perfluorohexane**	1.2	2.41	0.60	2.41

Figure S4j

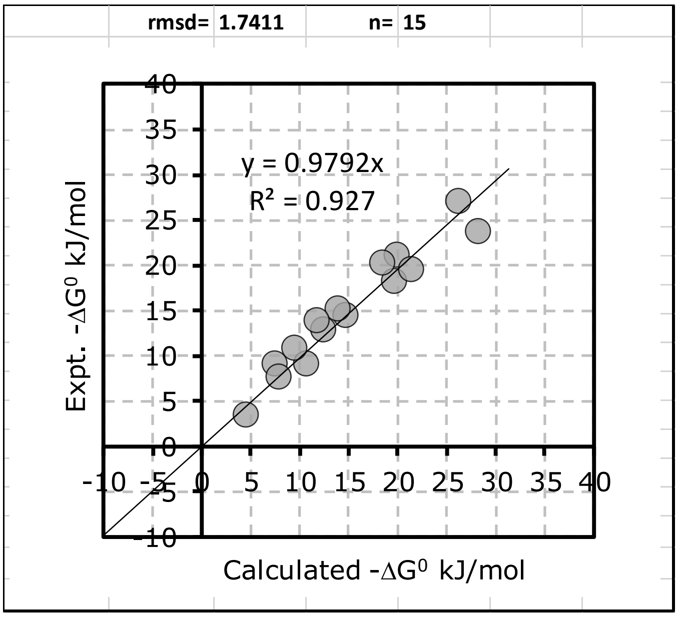

Free energy calculation for 1:1 Association

$$-\Delta G^0 = \alpha\beta - \alpha\beta_S - \alpha_S\beta - C_\alpha - C_\beta$$

Table S4j	Donor		Acceptor		Calc.	Expt.
**Reference**	**DH**	**Alpha**	**A**	**Beta**	**-ΔG⁰**	**-ΔG⁰**
**[1]**	2,2,2-trifluoroethanol	**3.70**	dimethyl sulphoxide	**8.60**	14.75	14.52
[1]	2,2,2-trifluoroethanol	**3.70**	Propan-2-one	**5.70**	7.50	9.05
[1]	2,2,2-trifluoroethanol	**3.70**	N,N-Dimethylformamide	**7.70**	12.50	12.83
[1]	2,2,2-trifluoroethanol	**3.70**	Tributylphosphine oxide	**10.70**	20.00	21.05
[1]	3-trifluoromethylphenol	**4.30**	Tributylphosphine oxide	**10.70**	26.24	26.97
[1]	3-trifluoromethylphenol	**4.30**	dimethyl sulphoxide	**8.60**	19.73	18.14
[1]	2,2,2-Trifluoro-1,1-bis(trifluoromethyl)ethanol	**4.90**	N,N-Dimethylformamide	**7.70**	21.38	19.55
[1]	Hexafluoropropan-2-ol	**4.50**	N,N-Dimethylformamide	**7.70**	18.42	20.29
[1]	2,2,2-Trifluoro-1,1-bis(trifluoromethyl)ethanol	**4.90**	Propan-2-one	**5.70**	13.98	15.15
[1]	Hexafluoropropan-2-ol	**4.50**	Propan-2-one	**5.70**	11.82	13.81
[1]	3-trifluoromethylphenol	**4.30**	Propan-2-one	**5.70**	10.74	9.11
[1]	2,2,2-Trifluoro-1,1-bis(trifluoromethyl)ethanol	**4.90**	Methyl formate	**4.50**	9.54	10.81
[1]	Hexafluoropropan-2-ol	**4.50**	Methyl formate	**4.50**	7.86	7.58
[1]	2,2,2-trifluoroethanol	**3.70**	Methyl formate	**4.50**	4.50	3.40
[55]	Hexafluoropropan-2-ol	**4.50**	Tributylphosphine oxide	**10.70**	28.32	23.74

References for Section 4

1. Cabot, R., C.A. Hunter, and L.M. Varley, *Hydrogen bonding properties of non-polar solvents.* Org Biomol Chem, 2010. **8**(6): p. 1455-62.
2. Cook, J.L., et al., *Solvent effects on hydrogen bonding.* Angew Chem Int Ed Engl, 2007. **46**(20): p. 3706-9.
3. Murthy, A.S.N. and C.N.R. Rao, *Spectroscopic Studies of the Hydrogen Bond.* Applied Spectroscopy Reviews, 1968. **2**(1): p. 69-191.
4. Góralski, P. and M. Tkaczyk, *Calorimetric investigations of association in ternary systems. Part 4.—The influence of solvation on enthalpy of complex formation in phenol-tetrahydrofuran and 2, 6-dimethylphenol–tetrahydrofuran systems.* Journal of the Chemical Society, Faraday Transactions 1: Physical Chemistry in Condensed Phases, 1987. **83**(9): p. 3083-3092.
5. Libuś, W., M. Mecik, and W. Sułek, *Solvent effect in hydrogen-bond formation between p-nitrophenol and amines.* Journal of Solution Chemistry, 1977. **6**(12): p. 865-879.
6. Grigorev, B., *Personal communication of data retrieved from HYBOT database (O. Raevsky, V. Grigorev, S. Trepalin, HYBOT program package, Registration by Russian State Patent Agency No. 990090 of 26.02.99).*
7. van Brabant-Govaerts, H. and P. Huyskens, *Comparative Study of Enthalpies of Hydrogen Bond Formation of Enamino and Amino Ketones.* Bulletin des Sociétés Chimiques Belges, 1981. **90**(10): p. 987-996.
8. Spencer, J., et al., *Solvation effects on the thermodynamics of hydrogen bonded systems. 3.* The Journal of Physical Chemistry, 1977. **81**(24): p. 2237-2240.
9. Spencer, J., R. Harner, and C. Penturelli, *Solvation effects on the thermodynamics of hydrogen bonding systems.* The Journal of Physical Chemistry, 1975. **79**(23): p. 2488-2493.
10. Spencer, J., et al., *Solvation effects on the thermodynamics of hydrogen bonded systems. II.* The Journal of Physical Chemistry, 1976. **80**(8): p. 811-814.
11. Spencer, J., et al., *Solvent effects on amine-n-butyl alcohol hydrogen-bonded complexes.* The Journal of Physical Chemistry, 1986. **90**(18): p. 4443-4447.
12. Spencer, J., et al., *Solvent effects on hydrogen-bond formation.* The Journal of Physical Chemistry, 1985. **89**(10): p. 1888-1891.
13. Codoñer, A., et al., *Determination of the complexes between dimethylphenols and pyridine by a dielectric constant method in non-polar solvents.* Journal of the Chemical Society, Perkin Transactions 2, 1986(4): p. 573-578.
14. Werner, R., J. Quinn, and J. Haken, *Intermolecular interaction in solution—IV. The influence of solvent on the association of proton donors and acceptors.* Spectrochimica Acta Part A: Molecular Spectroscopy, 1982. **38**(8): p. 887-897.
15. Prezhdo, V., O. Prezhdo, and E. Vaschenko, *Studies on the proton acceptor ability of phosphoryl compounds.* Journal of molecular structure, 1996. **385**(2): p. 137-144.
16. Adams, H., et al., *Quantification of the effect of conformational restriction on supramolecular effective molarities.* J Am Chem Soc, 2013. **135**(5): p. 1853-63.
17. Henkel, S. and C.A. Hunter, *Private Communication.* 2021.
18. Chekmeneva, E., et al., *Evidence for partially bound states in cooperative molecular recognition interfaces.* J Am Chem Soc, 2008. **130**(52): p. 17718-25.
19. Chekmeneva, E., et al., *Steric desolvation enhances the effective molarities of intramolecular H-bonding interactions.* Org Biomol Chem, 2012. **10**(30): p. 6022-31.
20. Hunter, C.A., M.C. Misuraca, and S.M. Turega, *Comparative analysis of the influence of H-bond strength and solvent on chelate cooperativity in H-bonded supramolecular complexes.* Chemical Science, 2012. **3**(8): p. 2462-2469.
21. Sun, H., et al., *Relationship between chemical structure and supramolecular effective molarity for formation of intramolecular H-bonds.* J Am Chem Soc, 2013. **135**(35): p. 13129-41.

22. Henkel, S., et al., *Enhanced Chelate Cooperativity in Polar Solvents.* Journal of the American Chemical Society, 2017. **139**(19): p. 6675-6681.
23. Robertson, C.C., et al., *Hydrogen bonding vs. halogen bonding: the solvent decides.* Chemical Science, 2017. **8**(8): p. 5392-5398.
24. Góralski, P. and M. Tkaczyk, *Calorimetric investigations of association in ternary systems. Part 4.—The influence of solvation on enthalpy of complex formation in phenol-tetrahydrofuran and 2,6-dimethylphenol–tetrahydrofuran systems.* Journal of the Chemical Society, Faraday Transactions 1: Physical Chemistry in Condensed Phases, 1987. **83**(9): p. 3083-3092.
25. Brandstädt, E., U. George, and A. Kolbe, *Hydrogen bonding between pyrrole and amines.* Journal of molecular liquids, 1985. **31**(2): p. 107-114.
26. Mendel, J., A. Mögel, and A. Kolbe, *H/D isotopic effect on the hydrogen bond between tertiary amines and alcohols.* Journal of molecular liquids, 1984. **29**(2): p. 127-134.
27. Cole, A.R.H., L.H. Little, and A.J. Michell, *Solvent effects in infra-red spectra. O ・E and S ・H stretching vibrations.* Spectrochimica Acta, 1965. **21**(7): p. 1169-1182.
28. Joesten, M.D. and L.J. Schaad, *Hydrogen Bonding*. 1974: Marcel Dekker, New York.
29. Laurence, C., et al., *An enthalpic scale of hydrogen-bond basicity. 4. Carbon pi bases, oxygen bases, and miscellaneous second-row, third-row, and fourth-row bases and a survey of the 4-fluorophenol affinity scale.* J Org Chem, 2010. **75**(12): p. 4105-23.
30. Abraham, M.H., et al., *Hydrogen-bonding. Part 4. An analysis of solute hydrogen-bond basicity, in terms of complexation constants (log K), using F1 and F2 factors, the principal components of different kinds of basicity.* Journal of Physical Organic Chemistry, 1989. **2**(3): p. 243-254.
31. Abboud, J.L.M., et al., *Studies on amphiprotic compounds. 4. Application of the .alpha.H2 hydrogen-bonding acidity scale to complexation between pyridine N-oxide and monomeric hydrogen-bond donors in cyclohexane.* The Journal of Organic Chemistry, 1990. **55**(7): p. 2230-2232.
32. Laurence, C. and M. Berthelot, *Observations on the strength of hydrogen bonding.* Perspectives in Drug Discovery and Design, 2000. **18**(1): p. 39-60.
33. Pimental, G.C. and A.L. McClellan, *The Hydrogen Bond*. 1960, San Francisco: W. H. Freeman.
34. Le Questel, J.-Y., M. Berthelot, and C. Laurence, *Can semi-empirical calculations yield reasonable estimates of hydrogen-bonding basicity? The case of nitriles.* Journal of the Chemical Society, Perkin Transactions 2, 1997(12): p. 2711-2718.
35. Koné, M., et al., *Can Quantum-Mechanical Calculations Yield Reasonable Estimates of Hydrogen-Bonding Acceptor Strength? The Case of Hydrogen-Bonded Complexes of Methanol.* The Journal of Physical Chemistry A, 2011. **115**(47): p. 13975-13985.
36. Graton, J., et al., *Hydrogen-Bond Acidity of OH Groups in Various Molecular Environments (Phenols, Alcohols, Steroid Derivatives, and Amino Acids Structures): Experimental Measurements and Density Functional Theory Calculations.* J Phys Chem A, 2013. **117**(49): p. 13184-93.
37. Ouvrard, C., Berthelot, M, and Laurence, C, *The first basicity scale of fluoro-, chloro-, bromo- and iodo-alkanes: some cross-comparisons with simple alkyl derivatives of other elements.* J Chem Soc [Perkin 2], 1999: p. 1357-1362.
38. Laurence, C., et al., *The pK(BHX) database: toward a better understanding of hydrogen-bond basicity for medicinal chemists.* J Med Chem, 2009. **52**(14): p. 4073-86.
39. Chardin, A., et al., *Hydrogen-bond basicity of the sulfonyl group. The case of strongly basic sulfonamidates RSO2NNMe3.* Journal of the Chemical Society, Perkin Transactions 2, 1996(6): p. 1047-1051.
40. Le Questel, J.-Y., et al., *Hydrogen-bond basicity of secondary and tertiary amides, carbamates, ureas and lactams.* Journal of the Chemical Society, Perkin Transactions 2, 1992(12): p. 2091-2094.

41. Berthelot, M., F. Besseau, and C. Laurence, *The Hydrogen-Bond Basicity pKHB Scale of Peroxides and Ethers.* European Journal of Organic Chemistry, 1998. **1998**(5): p. 925-931.
42. Besseau, F., J. Graton, and M. Berthelot, *A Theoretical Evaluation of the pKHB and ΔH Hydrogen-Bond Scales of Nitrogen Bases.* Chemistry – A European Journal, 2008. **14**(34): p. 10656-10669.
43. Besseau, F., C. Laurence, and M. Berthelot, *Hydrogen-bond basicity of esters, lactones and carbonates.* Journal of the Chemical Society, Perkin Transactions 2, 1994(3): p. 485-489.
44. Besseau, F., Lucon, M, Laurence, C and Berthelot, M, *Hydrogen-bond basicity pKHB scale of aldehydes and ketones.* J Chem Soc [Perkin 2], 1998: p. 101-108.
45. Berthelot, M., et al., *Hydrogen-bond basicity pKHB scale of six-membered aromatic N-heterocycles.* Journal of the Chemical Society, Perkin Transactions 2, 1998(2): p. 283-290.
46. Graton, J., et al., *Hydrogen-bond basicity pKHB scale of aliphatic primary amines.* Journal of the Chemical Society, Perkin Transactions 2, 1999(5): p. 997-1002.
47. Pike, S.J. and C.A. Hunter, *Fluorescent and colorimetric molecular recognition probe for hydrogen bond acceptors.* Organic & Biomolecular Chemistry, 2017. **15**(45): p. 9603-9610.
48. Berthelot, M., Laurence, C, Safar, M and Besseau, F, *Hydrogen-bond basicity pKHB scale of six-membered aromatic N-heterocycles* J Chem Soc [Perkin 2], 1998: p. 283-90.
49. Pike, S.J., J.J. Hutchinson, and C.A. Hunter, *H-Bond Acceptor Parameters for Anions.* Journal of the American Chemical Society, 2017. **139**(19): p. 6700-6706.
50. Wolff, H. and W. Zeller, *Infrared study of water-pentafluorophenol and water-4-fluorophenol complexes.* The Journal of Physical Chemistry, 1982. **86**(26): p. 5243-5247.
51. Werner, R.L., J.M. Quinn, and J.K. Haken, *Intermolecular interaction in solution—IV. The influence of solvent on the association of proton donors and acceptors.* Spectrochimica Acta Part A: Molecular Spectroscopy, 1982. **38**(8): p. 887-897.
52. Chardin, A., et al., *Carbonyl oxygen as a hydrogen-bond super-base: The amidates.* Journal of Physical Organic Chemistry, 1994. **7**(12): p. 705-711.
53. Dominelli-Whiteley, N., et al., *Strong Short-Range Cooperativity in Hydrogen-Bond Chains.* Angewandte Chemie International Edition, 2017. **56**(26): p. 7658-7662.
54. Kasende, O. and T. Zeegers-Huyskens, *Infrared study of hydrogen-bonded complexes involving phenol derivatives and polyfunctional bases. 2. 3-Methyl-4-pyrimidone, 1-methyl-2-pyrimidone, 1, 4, 4-trimethylcytosine, and 1, 3-dimethyluracil.* The Journal of Physical Chemistry, 1984. **88**(12): p. 2636-2641.
55. Hunter, C.A., *Corrected experimental value.* 2021.

Section 5:
Individually optimised solvent constants

Table S5:

Individually optimised constants for polar organic solvents and comparison of calculated transfer free energies with experimental data

	Conc. of polar atom. X=O or N	Solvent descriptors									solvent/water partition	
**Solvent**	**RTln[X] kJ mol^{-1}**	α_{S1}	$C_{\alpha 1}$	β_{S1}	$C_{\beta 1}$	α_{S2}	$C_{\alpha 2}$	β_{S2}	$C_{\beta 2}$	C_0	**N**	**rmsd kJ mol^{-1}**
Tetrahydrofuran	6.22	1.20	***2.62***	0.60	***2.78***			5.30	***-3.46***	***3.26***	49	1.6
Diethyl Ether	5.61	1.20	***2.54***	0.60	***3.07***			5.30	***-3.62***	***2.17***	25	1.1
Di-n-butyl ether	4.4	1.2	***2.63***	0.6	***3.09***			5.30	***-4.58***	***1.18***	35	1.0
Acetonitrile	7.31	1.20	***-0.78***	0.60	***2.56***	1.50	***2.68***	5.15	***-4.26***	***6.01***	24	1.7
Propionitrile	6.54	1.20	***-0.81***	0.60	***2.63***	1.50	***2.68***	5.15	***-3.45***	***3.80***	21	1.6
Butyronitrile	6.05	1.20	***-0.79***	0.60	***2.72***	1.50	***2.69***	5.15	***-3.10***	***1.41***	31	0.9
Acetone	6.45	1.20	***-0.60***	0.60	***2.77***	1.50	***2.71***	5.80	***-4.12***	***2.32***	46	1.4
Butanone	5.98	1.20	***-0.86***	0.60	***2.86***	1.50	***2.71***	5.80	***-4.12***	***1.82***	42	1.4
Cyclohexanone	5.62	1.20	***-0.90***	0.60	***2.82***	1.50	***2.72***	5.80	***-4.05***	***1.52***	30	1.3
Methanol	7.95	1.20	***2.77***	0.60	***2.65***	3.50	***-5.03***	6.90	***-6.49***	***2.69***	64	1.2
Ethanol	7.04	1.20	***2.77***	0.60	***2.85***	3.50	***-5.62***	6.90	***-6.39***	***0.91***	44	1.4
Propan-1-ol	6.42	1.20	***2.76***	0.60	***2.89***	3.50	***-5.97***	6.90	***-6.52***	***0.66***	48	1.3
Propan-2-ol	6.37	1.20	***2.73***	0.60	***2.99***	3.50	***-5.80***	6.90	***-6.35***	***-0.21***	62	1.5
Butan-1-ol	5.93	1.20	***2.68***	0.60	***2.98***	3.50	***-6.91***	6.90	***-6.26***	***0.73***	57	1.3
Butan-2-ol	5.92	1.20	***2.69***	0.60	***2.89***	3.50	***-6.39***	6.90	***-6.38***	***1.28***	29	1.3
2-Methylpropan-1-ol	5.90	1.20	***2.73***	0.60	***3.00***	3.50	***-5.94***	6.90	***-6.67***	***0.69***	38	1.1
2-Methylpropan-2-ol	5.82	1.20	***2.72***	0.60	***2.97***	3.50	***-5.93***	6.90	***-6.82***	***1.06***	44	1.4
Pentan-1-ol	5.50	1.20	***2.75***	0.60	***2.91***	3.50	***-6.31***	6.90	***-6.51***	***0.95***	44	1.1
3-Methylbutan-1-ol	5.50	1.20	***2.77***	0.60	***3.00***	3.50	***-5.68***	6.90	***-6.82***	***0.39***	22	0.9
Hexan-1-ol	5.16	1.20	***2.84***	0.60	***2.94***	3.50	***-5.90***	6.90	***-6.88***	***-0.38***	30	1.0
Heptan-1-ol	4.84	1.20	***2.76***	0.60	***3.02***	3.50	***-6.19***	6.90	***-6.30***	***-0.08***	21	0.8
Octan-1-ol	4.57	1.20	***2.76***	0.60	***3.03***	3.50	***-7.03***	6.90	***-6.66***	***-0.88***	74	1.2
Decan-1-ol	4.10	1.20	***2.83***	0.60	***2.95***	3.50	***-6.22***	6.90	***-6.49***	***-1.29***	27	0.8

Footnote to table S5.

The H-bond parameters α_S and β_S were previously derived using Equation (1) (see Section 6). The constants in italic bold were optimised in order to minimise the rmsd between calculated and experimental free energies in the solvent/water partition models.

Section 6:
Solvent H-bond parameters α_S and β_S

$$\Delta G° \text{ / kJ mol}^{-1} = -(\alpha - \alpha_S)(\beta - \beta_S) + 6 \qquad (1)$$

Table S6a

Values of α_S and β_S used to model partition into non-polar organic solvents as compared with published values derived from equation (1).

	Partition model	Association (eqn. 1)	Partition model	Association (eqn. 1)	
	α_S	Published range	β_S	Published range	References
**Simple solvents**					
Alkanes	**1.20**	0.4-1.2	**0.60**	0.3-0.6	[1-4]
Carbon tetrachloride	**1.40**		**0.60**		[1, 4-6]
Dichloromethane	**1.80**	1.7-1.9	**1.40**	1.4-2.0	[2, 4, 7, 8]
Chloroform	**2.10**	2.1-2.4	**1.30**	0.8-1.3	[1, 4-9]
1,2-Dichloroethane	**1.70**	1.7	**1.60**	1.6	[2]
Chlorobenzene	**1.40**	1.4	**1.40**	1.1-1.8	[2]
Benzene	**1.40**	1.0-1.3	**2.00**	1.6-2.2	[1, 2, 4]
Toluene	**1.40**	1.0-1.1	**2.00**	1.6-2.2	[7, 10-15]

Table S6b

Values of α_S and β_S used to model partition into polar organic solvents compared with published values derived from equation (1) (N.B. To model partition of solutes, additional descriptors are required to model the alkane component of the solvent with α_S =1.2 and β_S =0.6)

	Partition model	Association (eqn. 1)	Partition model	Association (eqn. 1)	
	α_S	Published range	β_S	Published range	References
**Ethers**					
Diethyl Ether	**1.20**		**5.30**		
Di-n-octyl ether		0.9		5-5.3	[11]
Di-n-hexyl ether		1.0		5.3	[3]
Di-n-butyl ether	**1.20**		**5.30**		
Tetrahydrofuran	**1.20**	0.9	**5.90**	5.3-5.9	[1, 9]
**Nitriles**					
Acetonitrile	**1.50**	1.5-1.7	**5.15**	4.7-5.1	[1, 5, 6]
Propionitrile	**1.50**		**5.15**		
Butyronitrile	**1.50**		**5.15**		
n-butyl cyanide		1.7		5.2	[11]
**Ketones**					
Acetone	**1.50**	1.2-1.5	**5.80**	5.7-5.8	[1, 4, 6, 7]
Butanone	**1.50**		**5.80**		
Cyclohexanone	**1.50**	1.5	**5.80**	5.8	[12]
2-Heptanone	**1.50**	1.5	**5.80**	5.8	[11]

References for Section 6

1. Cook, J.L., et al., *Solvent effects on hydrogen bonding.* Angew Chem Int Ed Engl, 2007. **46**(20): p. 3706-9.
2. Cabot, R., C.A. Hunter, and L.M. Varley, *Hydrogen bonding properties of non-polar solvents.* Org Biomol Chem, 2010. **8**(6): p. 1455-62.
3. Amenta, V., et al., *Molecular recognition probes of solvation thermodynamics in solvent mixtures.* Org Biomol Chem, 2011. **9**(21): p. 7571-8.
4. Cabot, R. and C.A. Hunter, *A thermodynamic study of selective solvation in solvent mixtures.* Org Biomol Chem, 2010. **8**(8): p. 1943-50.
5. Pike, S.J., J.J. Hutchinson, and C.A. Hunter, *H-Bond Acceptor Parameters for Anions.* Journal of the American Chemical Society, 2017. **139**(19): p. 6700-6706.
6. Pike, S.J., et al., *H-Bond donor parameters for cations.* Chemical Science, 2019. **10**(23): p. 5943-5951.
7. Chekmeneva, E., et al., *Evidence for partially bound states in cooperative molecular recognition interfaces.* J Am Chem Soc, 2008. **130**(52): p. 17718-25.
8. Pike, S.J. and C.A. Hunter, *Fluorescent and colorimetric molecular recognition probe for hydrogen bond acceptors.* Organic & Biomolecular Chemistry, 2017. **15**(45): p. 9603-9610.
9. Cook, J.L., et al., *Preferential solvation and hydrogen bonding in mixed solvents.* Angew Chem Int Ed Engl, 2008. **47**(33): p. 6275-7.
10. Hunter, C.A., M.C. Misuraca, and S.M. Turega, *Dissection of complex molecular recognition interfaces.* J Am Chem Soc, 2011. **133**(3): p. 582-94.
11. Amenta, V., et al., *Influence of solvent polarity on preferential solvation of molecular recognition probes in solvent mixtures.* J Phys Chem B, 2012. **116**(49): p. 14433-40.
12. Chekmeneva, E., et al., *Steric desolvation enhances the effective molarities of intramolecular H-bonding interactions.* Org Biomol Chem, 2012. **10**(30): p. 6022-31.
13. Adams, H., et al., *Quantification of the effect of conformational restriction on supramolecular effective molarities.* J Am Chem Soc, 2013. **135**(5): p. 1853-63.
14. Sun, H., et al., *Relationship between chemical structure and supramolecular effective molarity for formation of intramolecular H-bonds.* J Am Chem Soc, 2013. **135**(35): p. 13129-41.
15. Henkel, S., et al., *Enhanced Chelate Cooperativity in Polar Solvents.* Journal of the American Chemical Society, 2017. **139**(19): p. 6675-6681.

Section 7:
Calculated and experimental free energies of transfer in workbook Excel 1.xlsx

Free energies of transfer are expressed as $-\Delta G^0$ in kJ mol^{-1} and therefore increasing values of free energy correspond to increasing values of partition coefficient. Experimental and calculated free energies of transfer are listed and compared in the five worksheets of the workbook Excel 1.xlsx:

1 Training set expt. v calc.
This sheet contains a list of the 219 solutes that comprise the training set for the model. For each solute are listed the name and SMILES string with experimental and calculated free energy of transfer ($-\Delta G^0$) from water to 35 different solvents. A summary table (B2:D39) lists the number of data points and rmsd between calculated and experimental values for each solvent and a graph displays calculated (y axis) versus experimental (x axis) values.

2 Validation set expt. v calc.
This sheet contains a list of 84 solutes that had not been used for training the model. For each solute are listed the name and SMILES string with experimental and calculated free energy of transfer from water to hexadecane and water to wet octanol. The rmsd between calculated and experimental values for each solvent is summarised in a table (B2:D4) and a graph displays calculated (y axis) versus experimental (x axis) values.

3 Expt. v calc. by SSIMPLE
This sheet contains a list of the same set of 219 solutes as listed in sheet 1 together with experimental free energy of transfer ($-\Delta G^0$) from water to 34 different solvents and results of calculations performed using the SSIMPLE approach described previously[1, 2]. The SSIP descriptions of the molecules were obtained using the in house footprinting code (version 6.0.0, commit ID 18b2ca65) which implements these methods. A summary table (B2:D39) lists the number of data points and rmsd between values calculated with SSIMPLE and experimental values for each solvent and a graph displays calculated (y axis) versus experimental (x axis) values.

4 Octanol_water comparison
This sheet contains a list of 189 solutes for which free energy of transfer ($-\Delta G^0$) from water to wet octanol was available. These values are compared with calculated values using three different methods:
a) Abraham solvation equation[3] using solvent coefficients for octanol taken from reference [4]. Calculated logP values are in column I and are converted to $-\Delta G^0$ in column K.
Rmsd between calculated and experimental 1.1 kJ mol^{-1}
b) cLogP calculated using Advanced Algorithm Builder software [5]. Calculated logP values are in column N and are converted to $-\Delta G^0$ in column P.
Rmsd between calculated and experimental 0.8 kJ mol^{-1}
c) $-\Delta G^0$ calculated by our new method are in column T.
Rmsd between calculated and experimental 1.6 kJ mol^{-1}

5 Expt. gas to solvent
This sheet contains a list of the 219 solutes that were used as the training set for the model. For each solute is listed the name and SMILES string and experimental free energy of transfer ($-\Delta G^0$) from gas to 35 different solvents.

Sources of Data

Experimental values of gas to solvent transfer free energies were obtained from literature sources as described below. These values were used to obtain water to solvent transfer free energies.

Acree and co-workers have published Abraham model correlations for describing logK, where *K* is the dimensionless gas-to-solvent partition constant (see Eq. (S7.1))

$$K = \frac{\text{molar concentration of solute in extraction solvent}}{\text{molar concentration of solute in the gas phase}} \qquad \text{(S7.1)}$$

Experimental values of logK (with concentrations in each phase defined in terms of mol litre^{-1}) have been reported for more than 50 common solvents. The values for logK were usually derived by conversion from other experimental measures such as:

- Raoult's law infinite dilution activity coefficients, γ^{∞}_{solute}

  $$\log K = \log\left(\frac{RT}{\gamma^{\infty}_{solute} V_{solvent} \left(VP\right)^{0}_{solute}}\right)$$

  $R =$ Gas constant; $T =$ Temperature; $V_{solvent} =$ Molar volume of the solvent

  $\left(VP\right)^{0}_{solute} =$ Vapour pressure of the solute at T

- Henry's law constants, K_{Henry}

  $$\log K = \log\left(\frac{RT}{K_{henry} V_{solvent}}\right)$$

  $V_{solvent} =$ Molar volume of the solvent

- Solubilities: where data was available for crystalline solutes dissolved in both the anhydrous solvent and water and where the solute gas-to-water partition coefficient, K_w, is known.

Further details can be found in publications by Abraham, Acree and co-workers[6-13]

Experimentally determined values of logK at 298 K were extracted from the appropriate references and converted into the free energy ($-\Delta G^0$ /kJ mol^{-1}) for transfer from gas phase to solvent by the usual formula i.e. eqn. (S7.2).

$$-\Delta G^{0}_{Gas->Solvent} = RT \ln K = 2.303RT \log K$$
$$R = \text{Gas constant} = 0.0083145 \text{ kJ mol}^{-1} \text{ K}^{-1} \qquad \text{(S7.2)}$$
$$T = \text{Temperature K}$$

A list of solvents and references to the published data can be found in Table S7. Some additional values for partition of water from gas to propan-2-ol, acetone and tetrahydrofuran were derived from published values of the infinite infinite dilution activity coefficients. [14]

Free energies of transfer ($-\Delta G^0$) from water to wet octanol were derived from $\log P_{octanol}$ values extracted from various commercially available databases. If several alternative $\log P_{octanol}$ values were available then an average value was used.

The values of $-\Delta G^{0}_{Solvent1->Solvent2}$ observed for transfer of a solute between two solvents were calculated from the experimentally determined gas-to-solvent values (eqn. (S7.3)}. Values calculated in this way for partition between water and an organic solvent refer to a hypothetical dry solvent:

$$-\Delta G^{0}_{Solvent1->Solvent2} = -\Delta G^{0}_{Gas->Solvent2} - (-\Delta G^{0}_{gas->Solvent1}) \qquad \text{(S7.3)}$$

In order to conduct the feasibility study it was desirable to limit the size of the initial data set. Compounds were only selected if they had measured gas-to-solvent logK values available for water and several other solvents. An easily manageable set of 219 compounds was chosen as an initial training set and included a variety of common functional groups. Another set of 84 similar compounds was identified for which $\log P_{octanol}$ values and experimental gas to hexadecane and gas to water logK were available. This set of 84 solutes was used for validation of the parameters derived by analysis of the training set. New descriptors will need to be

added in future in order to extend the model to include solutes that contain other functiomal groups and to deal with intramolecular interactions.

Table S7: Solvents and references to experimental logK data.

Hexadecane (i.e. Abraham L descriptor)[6-13]	3-Methylbutan-1-ol[8]
Hexane[15-17]	2-Methylpropan-2-ol[8]
Cyclohexane[15, 16]	2-Methylpropan-1-ol[8]
Water[6, 10, 11, 18]	Butan-2-ol[8]
Carbon tetrachloride[9, 15, 16]	Propan-2-ol[8]
Acetone[10]	Decan-1-ol[8]
Tetrahydrofuran[13, 15, 16]	Octan-1-ol[8]
Diethyl Ether[8, 13]	Heptan-1-ol[8]
Di-n-butyl ether[8, 15, 16, 19]	Hexan-1-ol[8]
Acetonitrile[15, 16, 18]	Pentan-1-ol[8]
Propionitrile[7, 15, 16]	Butan-1-ol[8]
Butyronitrile[7, 15, 16]	Propan-1-ol[8, 18]
Butanone[6, 10]	Ethanol[8, 15, 16]
Cyclohexanone[6, 10]	Methanol[8]
Dichloromethane[9]	Chlorobenzene[11, 15, 16]
Chloroform[9, 15, 16]	Benzene[15, 16]
1,2-Dichloroethane[12, 15, 16]	Toluene[15, 16]
Perfluoroalkane[20]	

References for Section 7

1. Hunter, C.A., *A surface site interaction model for the properties of liquids at equilibrium.* Chem. Sci., 2013. **4**: p. 1687-1700.
2. Calero, C.S., et al., *Footprinting molecular electrostatic potential surfaces for calculation of solvation energies.* Phys Chem Chem Phys, 2013. **15**(41): p. 18262-73.
3. Abraham, M.H., *Scales of solute hydrogen-bonding: their construction and application to physicochemical and biochemical processes.* Chemical Society Reviews, 1993. **22**(2): p. 73-83.
4. Zissimos, A.M., et al., *Calculation of Abraham descriptors from solvent–water partition coefficients in four different systems; evaluation of different methods of calculation.* Journal of the Chemical Society, Perkin Transactions 2, 2002(3): p. 470-477.
5. Japertas, P., R. Didziapetris, and A. Petrauskas, *Fragmental Methods in the Design of New Compounds. Applications of Advanced Algorithm Builder.* . Quant. Struct.-Act. Relat., 2002. **21**: p. 23-37.
6. Tong, X., et al., *Updated Abraham model correlations for correlating solute transfer into dry butanone and dry cyclohexanone solvents.* Physics and Chemistry of Liquids, 2018. **56**(5): p. 571-583.
7. Hart, E., et al., *Development of Abraham model correlations for describing the transfer of molecular solutes into propanenitrile and butanenitrile from water and from the gas phase.* Physics and Chemistry of Liquids, 2018. **56**(6): p. 821-833.
8. Grubbs, L.M., et al., *Mathematical correlations for describing solute transfer into functionalized alkane solvents containing hydroxyl, ether, ester or ketone solvents.* Fluid Phase Equilibria, 2010. **298**(1): p. 48-53.
9. Sprunger, L.M., et al., *Correlation and prediction of solute transfer to chloroalkanes from both water and the gas phase.* Fluid Phase Equilibria 2009. **281**: p. 144–162.
10. Abraham, M.H., et al., *The partition of compounds from water and from air into wet and dry ketones.* New Journal of Chemistry, 2009. **33**(3): p. 568-573.
11. Abraham, M.H., et al., *Partition of compounds from water and from air into the wet and dry monohalobenzenes.* New Journal of Chemistry, 2009. **33**(8): p. 1685-1692.
12. Sprunger, L.M., et al., *Correlation and prediction of partition coefficients for solute transfer to 1,2-dichloroethane from both water and from the gas phase.* Fluid Phase Equilibria, 2008. **273**(1–2): p. 78-86.
13. Abraham, M.H., A.M. Zissimos, and J.W.E. Acree, *Partition of solutes into wet and dry ethers; an LFER analysis.* New Journal of Chemistry, 2003. **27**(7): p. 1041-1044.

14. Bergmann, D.L. and C.A. Eckert, *Measurement of limiting activity coefficients for aqueous systems by differential ebulliometry.* Fluid Phase Equilibria, 1991. **63**(1): p. 141-150.
15. Katritzky, A.R., et al., *A general treatment of solubility. 1. The QSPR correlation of solvation free energies of single solutes in series of solvents.* J Chem Inf Comput Sci, 2003. **43**(6): p. 1794-805.
16. Katritzky, A.R., et al., *A general treatment of solubility. 2. QSPR prediction of free energies of solvation of specified solutes in ranges of solvents.* J Chem Inf Comput Sci, 2003. **43**(6): p. 1806-14.
17. Stephens, T.W., et al., *Correlation of solute transfer into alkane solvents from water and fromthe gas phase with updated Abraham model equations.* . Global J. Phys. Chem., 2012. **3**: p. 1-42.
18. Katritzky, A.R., et al., *A general treatment of solubility. 3. Principal component analysis (PCA) of the solubilities of diverse solutes in diverse solvents.* J Chem Inf Model, 2005. **45**(4): p. 913-23.
19. Abraham, M.H., A.M. Zissimos, and W.E. Acree Jr, *Partition of solutes from the gas phase and from water to wet and dry di-n-butyl ether: a linear free energy relationship analysis.* Physical Chemistry Chemical Physics, 2001. **3**(17): p. 3732-3736.
20. Abraham, M.H., W.E. Acree, and E. Matteoli, *(Combined and averaged values for partition into perfluorohexane, perfluoroheptane and perfluorooctane) The factors that influence solubility in perfluoroalkane solvents.* Fluid Phase Equilibria, 2016. **421**: p. 59-66.

Section 8:
Correlation between Molecular Surface Area and the number of Surface Site Interaction Points

The Van der Waals surface areas were determined using the 0.002 e bohr^{-3} isosurface calculated with NWChem (Density Functional Theory B3LYP/6-31G* basis set)[1]. The surface areas were calculated by summing the number of points on the isosurface, but scaling the contribution of each point by the local density of points within a radius of 0.5 Å.

Figure S8. Plot of number of SSIPs (x axis) v molecular surface area (Å^2) (y axis)

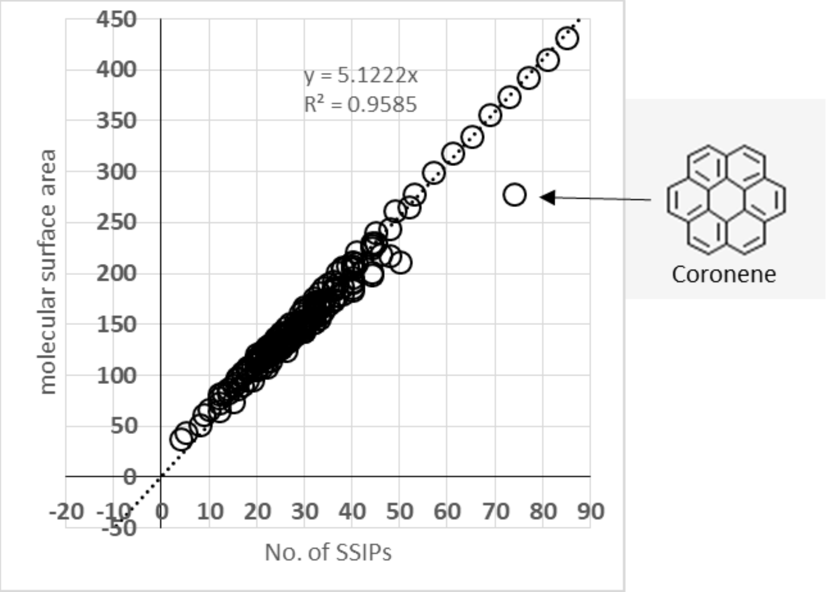

Table S8: Molecular Surface Areas and Numbers of SSIPs for 219 Compounds of the Training Set

SMILES	Solute name	Compound Class	Molecular Surface Area (Å^2)	No. of SSIPs
CCCCC	n-pentane	Alkane-Acyclic Linear	132.2	24
CCCCCC	n-hexane	Alkane-Acyclic Linear	152.8	28
CCCCCCC	n-heptane	Alkane-Acyclic Linear	172.4	32
CCCCCCCC	n-octane	Alkane-Acyclic Linear	191.9	36
CCCCCCCCC	n-nonane	Alkane-Acyclic Linear	211.6	40
CCCCCCCCCC	n-decane	Alkane-Acyclic Linear	230.7	44
CCC(C)C	isopentane	Alkane Acyclic Branched	128.5	24
CCCC(C)C	2-methylpentane	Alkane Acyclic Branched	148.2	28
CCC(C)CC	3-methylpentane	Alkane Acyclic Branched	145.7	28
CCCC(C)CC	3-methylhexane	Alkane Acyclic Branched	165.9	32
CCCC(C)(C)C	2,2-dimethylpentane	Alkane Acyclic Branched	160.9	32
CCCCC(C)CC	3-methylheptane	Alkane Acyclic Branched	185.7	36
CC(C)CCC(C)C	2,5-dimethylhexane	Alkane Acyclic Branched	183.3	36
CC(C)CC(C)(C)C	2,2,4-trimethylpentane	Alkane iso-octane	175.3	36
CC(C)C(C)C(C)C	2,3,4-trimethylpentane	Alkane iso-octane	173.2	36
CCCCCCC(C)C	2-methyloctane	Alkane Acyclic Branched	208.0	40
CC(C)CCC(C)(C)C	2,2,5-trimethylhexane	Alkane Acyclic Branched	195.9	40
CCC(CC)(CC)CC	3,3-diethylpentane	Alkane Acyclic Branched	185.6	40
CCCCCCCC(C)C	2-methylnonane	Alkane Acyclic Branched	227.1	44
C1CCCC1	cyclopentane	Alkane-Cyclic	115.2	20
C1CCCCC1	cyclohexane	Alkane-Cyclic	130.1	24
C1CCCCCCC1	cyclooctane	Alkane-Cyclic	162.7	32
CC1CCCC1	methylcyclopentane	Alkane-Cyclic Branched	133.6	24
CC1CCCCC1	methylcyclohexane	Alkane-Cyclic Branched	147.5	28
CCC1CCCCC1	ethylcyclohexane	Alkane-Cyclic Branched	165.2	32
C[C@@H]1([C@@H](CCCC1)(C))	cis-1,2-dimethylcyclohexane	Alkane-Cyclic Branched	162.7	32
CC1CCC(C)CC1	trans-1,4-dimethylcyclohexane	Alkane-Cyclic Branched	165.2	32

[Cl]C([Cl])([Cl])[Cl]	tetrachloromethane	Chlorosolvent-CCl4	121.1	20
c1ccccc1	benzene	Benzene	112.2	20
O([H])[H]	water	Water	37.5	4
CO	methanol	Alcohol-Methanol	61.4	9
CCO	ethanol	Alcohol-Primary	82.3	13
CCCO	propan-1-ol	Alcohol-Primary	102.5	17
CCCCO	butan-1-ol	Alcohol-Primary	122.0	21
CCCCCO	pentan-1-ol	Alcohol-Primary	142.0	25
CCCCCCO	hexan-1-ol	Alcohol-Primary	161.5	29
CCCCCCCO	heptan-1-ol	Alcohol-Primary	181.5	33
CCCCCCCCO	octan-1-ol	Alcohol-Primary	201.3	37
CCCCCCCCCO	nonan-1-ol	Alcohol-Primary	221.5	41
CCCCCCCCCCO	decan-1-ol	Alcohol-Primary	240.9	45
COC	dimethylether	Ether-Dimethyl	83.0	14
CCOCC	diethyl ether	Ether-Dialkyl	124.8	22
CCCOCCC	dipropylether	Ether-Dialkyl	164.4	30
CCCCOCCCC	dibutylether	Ether-Dialkyl	204.5	38
[Cl]c1ccccc1	chlorobenzene	Benzene-Chloro	128.9	24
[Cl]c1ccccc1[Cl]	1,2-dichlorobenzene	Benzene-Dichloro	144.6	28
[Cl]CC[Cl]	1,2-dichloroethane	Chlorosolvent-CH2ClCH2Cl	108.5	18
C	methane	Alkane-Methane	50.9	8
CC	ethane	Alkane-Acyclic Linear	72.8	12
CCC	propane	Alkane-Acyclic Linear	93.1	16
CCCC	butane	Alkane-Acyclic Linear	112.7	20
CN(C)C	trimethylamine	Amine-Tertiary	105.4	20
CCN(CC)CC	triethylamine	Amine-Tertiary	161.2	32
C1CCNCC1	piperidine	Amine-Secondary Cyclic	125.7	23
CCNCC	diethylamine	Amine-Secondary	127.9	23
CCCNCCC	dipropylamine	Amine-Secondary	167.5	31
CCCCNCCCC	dibutylamine	Amine-Secondary	207.2	39
CN	methylamine	Amine-Primary	66.9	10
CCN	ethylamine	Amine-Primary	87.2	14
CCCN	n-propylamine	Amine-Primary	107.3	18
CCCCN	n-butylamine	Amine-Primary	127.0	22
CCCCCN	pentylamine	Amine-Primary	146.6	26
CCCCCCN	hexylamine	Amine-Primary	166.6	30
CCCCCCCN	heptylamine	Amine-Primary	185.9	34
CCCCCCCCN	n-octylamine	Amine-Primary	205.9	38
NC1CCCCC1	cyclohexylamine	Amine-Primary	142.4	26
CCc1ccccc1	ethyl benzene	Benzene-Alkyl Substituted	150.9	29
Cc1ccccc1	toluene	Benzene-Methyl substituted	131.1	25
Cc1ccccc1C	o-xylene	Benzene-Methyl substituted	147.4	30
Cc1cccc(C)c1	m-xylene	Benzene-Methyl substituted	150.7	30
Cc1ccc(C)cc1	p-xylene	Benzene-Methyl substituted	150.8	30
Cc1cc(C)cc(C)c1	mesitylene	Benzene-Methyl substituted	170.3	35
Cc1cc(C)c(C)cc1C	1,2,4,5-tetramethylbenzene	Benzene-Methyl substituted	183.8	40
CC(C)=O	propanone	Ketone-Dialkyl	95.7	18
CCC(C)=O	butanone	Ketone-Dialkyl	114.6	22
CCCC(C)=O	pentan-2-one	Ketone-Dialkyl	134.2	26
CCC(=O)CC	pentan-3-one	Ketone-Dialkyl	133.7	26
CCCCC(C)=O	hexan-2-one	Ketone-Dialkyl	153.9	30
CCCC(=O)CC	hexan-3-one	Ketone-Dialkyl	153.4	30
CCCC(=O)CCC	heptan-4-one	Ketone-Dialkyl	173.3	34
CC(C)CC(C)=O	4-methylpentan-2-one	Ketone-Dialkyl	149.7	30
O=C1CCCC1	cyclopentanone	Ketone-Cycloalkyl	118.4	22
O=C1CCCCC1	cyclohexanone	Ketone-Cycloalkyl	133.3	26
O=C1CCCCCC1	cycloheptanone	Ketone-Cycloalkyl	148.5	30
O=C1CCCCCCC1	cyclooctanone	Ketone-Cycloalkyl	163.0	34
Oc1ccccc1	phenol	Phenol	120.3	23
Cc1cccc(O)c1	3-methylphenol	Phenol-methyl substituted	140.1	28
Cc1ccc(O)cc1	4-methylphenol	Phenol-methyl substituted	140.3	28
Cc1ccc(O)cc1C	3,4-dimethylphenol	Phenol-methyl substituted	156.3	33
Cc1cc(C)cc(O)c1	3,5-dimethylphenol	Phenol-methyl substituted	159.3	33
Oc1cccc([Cl])c1	3-chlorophenol	Phenol-Chloro substituted	138.1	27
Oc1ccc([Cl])cc1	4-chlorophenol	Phenol-Chloro substituted	138.4	27
Oc1ccc([Cl])c([Cl])c1	3,4-dichlorophenol	Phenol-Chloro substituted	153.6	31
Oc1cc([Cl])cc([Cl])c1	3,5-dichlorophenol	Phenol-Chloro substituted	155.4	31
[Cl]CC	chloroethane	Alkane-Monochloro	91.0	15
CCC[Cl]	1-chloropropane	Alkane-Monochloro	110.6	19

CCCC[Cl]	1-chlorobutane	Alkane-Monochloro	130.8	23
[Cl]CCC[Cl]	1,3-dichloropropane	Alkane-Dichloro	128.6	22
CCCCC[Cl]	1-chloropentane	Alkane-Monochloro	150.3	27
CCCCCCC[Cl]	1-chloroheptane	Alkane-Monochloro	190.3	35
c1ccc2ccccc2c1	naphthalene	Polycyclic aromatic	156.9	32
c1ccc2c(c1)ccc3ccccc23	phenanthrene	Polycyclic aromatic	198.5	44
c1ccc2cc3ccccc3cc2c1	anthracene	Polycyclic aromatic	200.7	44
c1cc2ccc3cccc4ccc(c1)c2c34	pyrene	Polycyclic aromatic	211.7	50
c1ccncc1	pyridine	Pyridine	107.5	20
CC#N	acetonitrile	Nitrile	73.9	15
CCC#N	propionitrile	Nitrile	94.9	19
CCCC#N	1-cyanopropane	Nitrile	114.7	23
CCCCC#N	1-cyanobutane	Nitrile	134.4	27
c1ccc(cc1)c1ccccc1	biphenyl	Benzene-Biphenyl	187.2	38
N	ammonia	Amine-NH3	44.3	5
CC(C)(C)[Cl]	t-butyl chloride	Alkane-Monochloro	124.7	23
C1CCOCC1	tetrahydropyran	Ether cyclic	122.0	22
C1CCOC1	tetrahydrofuran	Ether cyclic	106.8	18
C1CCSC1	tetrahydrothiophene	Sulfide-cycloalkyl	117.5	20
CCc1ccc(O)cc1	4-ethylphenol	Phenol-alkyl substituted	159.7	32
O=C1CCCCN1	d-valerolactam	Amide	128.8	25
CN1C(=O)CCCC1	N-methyl-delta-valerolactam	Amide	146.3	30
CN(C)C(C)=O	n,n-dimethylacetamide	Amide	125.0	26
CN1CCCCC1	n-methylpiperidine	Amine-Tertiary Cyclic	143.3	28
CC1CCCCN1	2-methylpiperidine	Amine-Secondary Cyclic	143.4	27
CC1CCCNC1	3-methylpiperidine	Amine-Secondary Cyclic	143.2	27
CC1CCNCC1	4-methylpiperidine	Amine-Secondary Cyclic	143.1	27
CN1CCCC1=O	n-methyl-2-pyrrolidinone	Amide	132.2	26
CSC	dimethylsulfide	Sulfide-Dialkyl	96.2	16
CCSCC	diethylsulfide	Sulfide-Dialkyl	136.1	24
CCCSCCC	dipropylsulfide	Sulfide-Dialkyl	175.7	32
CC(C)SC(C)C	diisopropylsulfide	Sulfide-Dialkyl	167.4	32
CSCC	methylethylsulfide	Sulfide-Dialkyl	116.4	20
C=C	ethene	Alkene-ethene	64.8	12
CC=C	propene	Alkene-methyl substituted	86.2	16
C=CCC	but-1-ene	Alkene-monoalkyl substituted	106.1	20
CCCC=C	pent-1-ene	Alkene-monoalkyl substituted	126.0	24
CCCCC=C	hex-1-ene	Alkene-monoalkyl substituted	145.9	28
CCCCCC=C	hept-1-ene	Alkene-monoalkyl substituted	165.9	32
CCCCCCC=C	oct-1-ene	Alkene-monoalkyl substituted	185.3	36
CCCCCCCC=C	non-1-ene	Alkene-monoalkyl substituted	205.4	40
CCCCCCCCC=C	dec-1-ene	Alkene-monoalkyl substituted	224.7	44
CCCCCCCCCC=C	undec-1-ene	Alkene-monoalkyl substituted	244.7	48
CCCCCCCCCCC=C	dodec-1-ene	Alkene-monoalkyl substituted	265.0	52
CNC=O	n-methylformamide	Amide	90.2	17
CN(C)C=O	n,n-dimethylformamide	Amide	108.2	22
CCCCN(C=O)CCCC	n,n-dibutylformamide	Amide	219.2	46
CNC(C)=O	n-methylacetamide	Amide	108.7	21
CCNC(C)=O	n-ethylacetamide	Amide	129.5	25
CCCCNC(C)=O	n-butylacetamide	Amide	168.5	33
CCOC	methylethylether	Ether-Dialkyl	103.8	18
COC1CCCCC1	methylcyclohexylether	Ether-Dialkyl	157.9	30
CCCCCCCCCCCO	undecan-1-ol	Alcohol-Primary	261.1	49
CCCCCCCCCCCCO	dodecan-1-ol	Alcohol-Primary	277.7	53
CCCCCCCCCCCCCO	tridecan-1-ol	Alcohol-Primary	299.3	57
CCCCCCCCCCCCCCO	tetradecan-1-ol	Alcohol-Primary	318.4	61
CCCCCCCCCCCCCCCO	pentadecan-1-ol	Alcohol-Primary	335.1	65
CCCCCCCCCCCCCCCCO	hexadecan-1-ol	Alcohol-Primary	356.0	69
CCCCCCCCCCCCCCCCCO	heptadecan-1-ol	Alcohol-Primary	373.6	73
CCCCCCCCCCCCCCCCCCO	octadecan-1-ol	Alcohol-Primary	392.5	77
CCCCCCCCCCCCCCCCCCCO	nonadecan-1-ol	Alcohol-Primary	409.8	81
CCCCCCCCCCCCCCCCCCCCO	eicosan-1-ol	Alcohol-Primary	432.0	85
OC12CC3CC(C1)CC(C2)C3	adamantan-1-ol	Alcohol-Tertiary	170.7	33
CC(C)O	propan-2-ol	Alcohol-Secondary	101.1	17
CCC(C)O	butan-2-ol	Alcohol-Secondary	119.9	21
CCCC(C)O	pentan-2-ol	Alcohol-Secondary	139.2	25
CCC(C)(C)O	2-methylbutan-2-ol	Alcohol-Tertiary	134.1	25
CC(C)(C)O	2-methylpropan-2-ol	Alcohol-Tertiary	117.3	21
CCCC(C)(C)O	2-methylpentan-2-ol	Alcohol-Tertiary	153.5	29

CCCCC(C)(C)O	2-methylhexan-2-ol	Alcohol-Tertiary	173.5	33
OC1CCCC1	cyclopentanol	Alcohol-Secondary	123.7	21
OC1CCCCC1	cyclohexanol	Alcohol-Secondary	137.8	25
OC1CCCCCC1	cycloheptanol	Alcohol-Secondary	152.6	29
OC1CCCCCCC1	cyclooctanol	Alcohol-Secondary	169.9	33
C1C2CC3CC1CC(C2)C3	adamantane	Alkane-Cyclic	164.1	32
CC(=O)C1CCCCC1	cyclohexylmethylketone	Ketone-Dialkyl	169.6	34
CC1CCCCC1=O	2-methylcyclohexanone	Ketone-Cycloalkyl	148.9	30
C1CC1	cyclopropane	Alkane-Cyclic	81.2	12
CCCC1CCCC1	propylcyclopentane	Alkane-Cyclic Branched	171.0	32
CCCCCC1CCCC1	pentylcyclopentane	Alkane-Cyclic Branched	210.7	40
CCCC1CCCCC1	propylcyclohexane	Alkane-Cyclic Branched	185.3	36
CCCCC1CCCCC1	butylcyclohexane	Alkane-Cyclic Branched	204.9	40
CC(C)(C)C1CCCCC1	t-butylcyclohexane	Alkane-Cyclic Branched	191.2	40
Cc1ccccn1	2-methylpyridine	Pyridine-Methyl substituted	127.4	25
Cc1cccnc1	3-methylpyridine	Pyridine-Methyl substituted	127.2	25
Cc1ccncc1	4-methylpyridine	Pyridine-Methyl substituted	127.1	25
Cc1cccnc1C	2,3-dimethylpyridine	Pyridine-Methyl substituted	143.9	30
Cc1ccnc(C)c1	2,4-dimethylpyridine	Pyridine-Methyl substituted	147.0	30
Cc1ccc(C)nc1	2,5-dimethylpyridine	Pyridine-Methyl substituted	147.0	30
Cc1cccc(C)n1	2,6-dimethylpyridine	Pyridine-Methyl substituted	147.1	30
Cc1cnccc1C	3,4-dimethylpyridine	Pyridine-Methyl substituted	143.5	30
Cc1cncc(C)c1	3,5-dimethylpyridine	Pyridine-Methyl substituted	146.4	30
c1ccc2ncccc2c1	quinoline	Pyridine-Quinoline	152.3	32
c1ccc2cnccc2c1	isoquinoline	Pyridine-Isoquinoline	151.7	32
CCCc1ccccc1	propylbenzene	Benzene-Alkyl Substituted	170.4	33
CCCCc1ccccc1	butylbenzene	Benzene-Alkyl Substituted	190.5	37
CCCCCc1ccccc1	pentylbenzene	Benzene-Alkyl Substituted	210.2	41
CCCCCCc1ccccc1	hexylbenzene	Benzene-Alkyl Substituted	229.8	45
CC(C)c1ccccc1	isopropylbenzene	Benzene-Alkyl Substituted	166.6	33
CC(C)(C)c1ccccc1	tert-butylbenzene	Benzene-Alkyl Substituted	178.6	37
[Cl]c1cccc([Cl])c1	1,3-dichlorobenzene	Benzene-Dichloro	146.7	28
[Cl]c1ccc([Cl])cc1	1,4-dichlorobenzene	Benzene-Dichloro	146.9	28
c1ccc2c(c1)c3cccc4cccc2c34	fluoranthene	Polycyclic aromatic	217.4	48
c1cc2ccc3ccc4ccc5ccc6ccc1c7c2c3c4c5c67	coronene	Polycyclic aromatic	278.0	74
C1Cc2cccc3cccc1c23	acenaphthene	Polycyclic aromatic	179.9	38
C1c2ccccc2c2ccccc12	fluorene	Benzene-Biphenyl	192.8	40
C1Cc2ccccc2C1	indane	Benzene-indane	155.0	30
CCF	fluoroethane	Alkane-Monofluoro	77.6	12
CCCF	1-fluoropropane	Alkane-Monofluoro	97.6	16
CC(C)F	2-fluoropropane	Alkane-Monofluoro	96.9	16
CCCCF	1-fluorobutane	Alkane-Monofluoro	117.5	20
CCCCCF	1-fluoropentane	Alkane-Monofluoro	137.0	24
Fc1ccccc1	fluorobenzene	Benzene-Monofluoro	116.3	21
Fc1ccccc1F	1,2-difluorobenzene	Benzene-Difluoro	121.1	22
Fc1ccccc1[Cl]	o-fluorochlorobenzene	Benzene-ChloroFluoro	133.5	25
Fc1cccc([Cl])c1	m-fluorochlorobenzene	Benzene-ChloroFluoro	134.2	25
Fc1ccc([Cl])cc1	p-fluorochlorobenzene	Benzene-ChloroFluoro	133.8	25
Oc1cccc(F)c1	3-fluorophenol	Phenol-Fluoro substituted	125.3	24
Oc1ccc(F)cc1	4-fluorophenol	Phenol-Fluoro substituted	125.3	24
CC=CC	cis-but-2-ene	Alkene-methyl substituted	107.2	20
CC=CC	trans-but-2-ene	Alkene-methyl substituted	107.2	20
CC=C(C)C	2-methylbut-2-ene	Alkene-methyl substituted	123.2	24
CC(C)(C)C	2,2-dimethylpropane	Alkane Acyclic Branched	125.8	24

Reference for Section 8

1. Aprà, E., et al., *NWChem: Past, present, and future.* The Journal of Chemical Physics, 2020. **152**(18): p. 184102.

Section 9:
Description of calculation procedure exemplified in workbook Excel 2.xlsx

The calculation procedure is exemplified for 266 solutes in four worksheets of the workbook Excel 2.xlscx, and the fifth worksheet lists the experimental partition data used in the development of the model. In the first two sheets, the calculated free energy contributions for transfer from the reference state to solvent are summed for each compound in the training set and used to compute free energies of transfer from water to organic solvent.

1 ΔG water->nonpolar
This sheet contains the parameters for water and a table of non-polar solvents and their associated parameters (blue cells). Entering a solvent ID from the table into the highlighted yellow cell (L4) pulls in data from the other sheets and calculates the free energies of transfer from water into the non-polar solvent selected. Plots are shown of the calculated (x axis) versus the experimental (y axis) free energies of transfer.

2 ΔG water->polar
This sheet contains a table of polar organic solvents and their associated parameters (blue cells). Entering a solvent ID from the table into the highlighted yellow cell (L4) pulls in data from the other sheets and calculates the free energies of transfer. Plots are shown of the calculated (x axis) versus the experimental (y axis) free energies of transfer.

3 Solute Functional Groups
This sheet contains a table of the functional group fragmentation of the training set of compounds (blue cells). Data are pulled in from the other sheets and used to calculate the solvation energies of each fragment in three different solvents (water, the non-polar solvent selected in cell L4 on the *ΔG water->nonpolar* sheet, and the polar solvent selected in cell L4 on the *ΔG water->nonpolar* sheet).

4 SSIP ΔGs
This sheet contains a table of the SSIP description of solute functional groups and the associated parameters (blue cells). Data are pulled in from the other sheets, and the solvation energies of the SSIPs are summed to obtain solvation energies for each functional group in three different solvents (water, the non-polar solvent selected in cell L4 on the *ΔG water->nonpolar* sheet, and the polar solvent selected in cell L4 on the *ΔG water->nonpolar* sheet).

5 Experimental Data
This sheet tabulates experimental free energies ($-\Delta G^o$) for solute transfer from water to organic solvents.
